# A Two-Dimensional Metallacycle Cross-Linked Switchable Polymer for Fast and Highly Efficient Phosphorylated Peptide Enrichment

**DOI:** 10.1021/jacs.0c12904

**Published:** 2021-05-27

**Authors:** Li-Jun Chen, Sean J. Humphrey, Jun-Long Zhu, Fan-Fan Zhu, Xu-Qing Wang, Xiang Wang, Jin Wen, Hai-Bo Yang, Philip A. Gale

**Affiliations:** † School of Chemistry, The University of Sydney, Sydney, NSW 2006, Australia; ‡ School of Life and Environmental Sciences, The University of Sydney, Sydney, NSW 2006, Australia; ∥ Shanghai Key Laboratory of Green Chemistry and Chemical Processes, Chang-Kung Chuang Institute, School of Chemistry and Molecular Engineering, East China Normal University, Shanghai 200062, China; □ State Key Laboratory for Modification of Chemical Fibers and Polymer Materials & College of Materials Science and Engineering, Donghua University, Shanghai 201620, China; ¶ Institute of Theoretical Chemistry, Faculty of Vienna, University of Vienna, Währinger Straße 17, A-1090 Vienna, Austria; § The University of Sydney Nano Institute (Sydney Nano), The University of Sydney, Sydney, NSW 2006, Australia

## Abstract

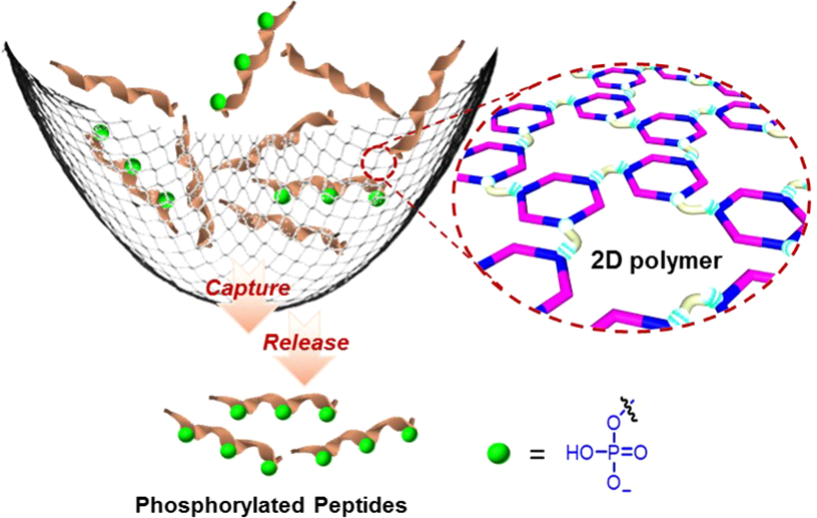

The selective and efficient capture of phosphopeptides is critical for comprehensive and in-depth phosphoproteome analysis. Here we report a new switchable two-dimensional (2D) supramolecular polymer that serves as an ideal platform for the enrichment of phosphopeptides. A well-defined, positively charged metallacycle incorporated into the polymer endows the resultant polymer with a high affinity for phosphopeptides. Importantly, the stimuli-responsive nature of the polymer facilitates switchable binding affinity of phosphopeptides, thus resulting in an excellent performance in phosphopeptide enrichment and separation from model proteins. The polymer has a high enrichment capacity (165 mg/g) and detection sensitivity (2 fmol), high enrichment recovery (88%), excellent specificity, and rapid enrichment and separation properties. Additionally, we have demonstrated the capture of phosphopeptides from the tryptic digest of real biosamples, thus illustrating the potential of this polymeric material in phosphoproteomic studies.

## Introduction

Anion recognition and extraction have received considerable attention during the last few decades since anionic species play many important roles in a number of areas including biology, pharmacy, industry, and environmental sciences.^1,2^ To date, a wide variety of functional groups and molecules have been exploited to selectively recognize, respond to, or sense anionic species.^3−7^ Recent results indicate that polymeric systems with *bona fide* anion recognition features can offer special advantages for the development of smart materials with applications in anion recognition and extraction.^8−11^ However, comprehensive analysis and identification of anionic biomolecules such as phosphorylated peptides (PPs) remain a challenge owing to anions’ high hydration and PPs’ low abundance as well as significant signal suppression by nonphosphorylated peptides.^12−14^ Anionic biomolecules such as PPs are closely associated with a number of human diseases, including Alzheimer’s disease and cancer.^15−17^ Thus, the development of supramolecular materials that can controllably bind and release PPs under aqueous conditions has numerous potential bioanalytical applications.

One of the most successful approaches to date for the recognition of phosphate derivate species in water is the use of coordination complexes, inspired by the fact that metal cations are commonly found in the binding sites of phosphate-binding proteins.^18,19^ Over the past few decades, discrete supramolecular coordination complexes (SCCs) with well-defined size, shape, and geometry have been widely constructed through coordination-driven self-assembly.^20−25^ These systems have found applications in molecular recognition, sensing, catalysis, biomedicines, and other areas.^26−30^ More recently, significant progress has been made on the functionalization of coordination metal complexes to construct stimuli-responsive supramolecular polymers which combine the advantages of well-defined supramolecular coordination complexes and polymeric materials, though either hierarchical self-assembly or postassembly polymerization.^31−39^ Motivated by our previous studies on discrete metallacycles^40^ and phosphate binding,^41,42^ we envisioned that a metallacyclic scaffold with well-defined shape and size may provide an ideal platform to construct polymers that can work as affinity reagents for the recognition of PPs. The abundance of reactive sites on polymeric scaffolds may enhance binding amount and provides multipoint attachment between the target and the matrix, which thus provides an opportunity to realize highly selective and tunable capture of PPs.

In this paper, we report a new kind of two-dimensional (2D) covalently linked metallacycle-cored polymer (polymer **5**) by combining coordination-driven self-assembly and postassembly reaction. The supramolecular polymer contains both H-bonding-based (urea) and electrostatic interaction-based (metallacycle) phosphate recognition units, facilitating strong and highly specific binding. Polymer **5** displayed a unique 2D morphology that has binding sites at both surfaces, permitting fast reactions with PPs, while nonphosphopeptides can be removed by washing with water. Moreover, the dynamic nature of metal–ligand bonds endows the polymer with excellent tunability and controllability over interactions with substrates. The unique stimuli-responsive properties facilitate rapid and complete separation of the affinity material. Using this 2D metallacycle-cored polymer **5** as an affinity material, we have quantitatively identified different synthetic standard peptides, demonstrating high enrichment capacity (up to 165 mg/g) and recoverability (>65% for all standard peptides). Additionally, the 2D metallacycle-cored polymer **5** is generally applicable to the analysis of PPs in complex protein mixtures, as well as in the tryptic digest of nonfat milk, demonstrating the validity of this enrichment approach and illustrating its potential use in proteomics applications.

## Results and Discussion

### Design, Synthesis, and Characterization of 2D Metallacycle-Cored Polymer

**Figure 1:**
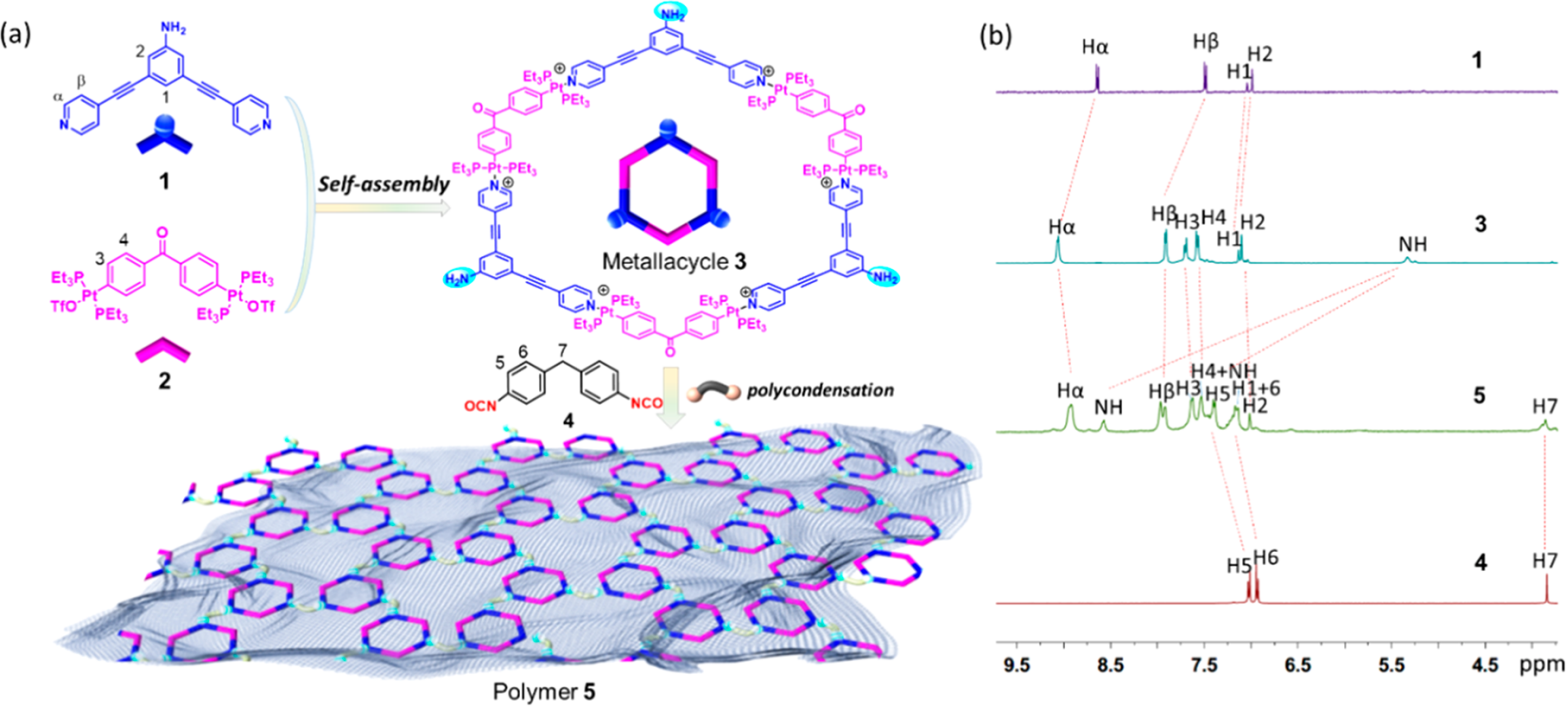
(a) Schematic representation of free-standing 2D polymer formation by combining coordination-driven self-assembly and postassembly reaction from simple organic ligands. (b) Partial ^1^H NMR spectra (in DMSO-*d*_6_, 400 MHz, 298 K) of 120° dipyridyl donor **1**, hexagonal metallacycle **3**, supramolecular polymer **5**, and methylene diphenyldiisocyanate **4**.

To accomplish the efficient construction of a metallacycle-cored supramolecular polymer, metallacycles with functional reactive sites should be both simple and stable, and the reaction used to link the supramolecular metallacycles should be mild and highly efficient. By examining several metallacycles and chemical reactions, a donor ligand **1**, containing two pyridyl units for metal coordination and an amino group for polymerization, was identified (Figure 1a). The hexagonal metallacycle **3** was prepared in quantitative yield by mixing the 120° dipyridyl donor **1** with 120° platinum acceptor **2** in a 1:1 ratio (Figure 1a and Scheme S1). The quantitative formation of amine-containing metallacycle **3** was confirmed by multinuclear (^1^H and ^31^P) NMR (Figures 1b and S1, S3, and S4), 2D COSY and ^1^H–^1^H NOESY NMR spectroscopy (Figures S5 and S6), and electrospray ionization time-of-flight mass spectrometry (ESI-TOF-MS) (Figure S7).

**Figure 2:**
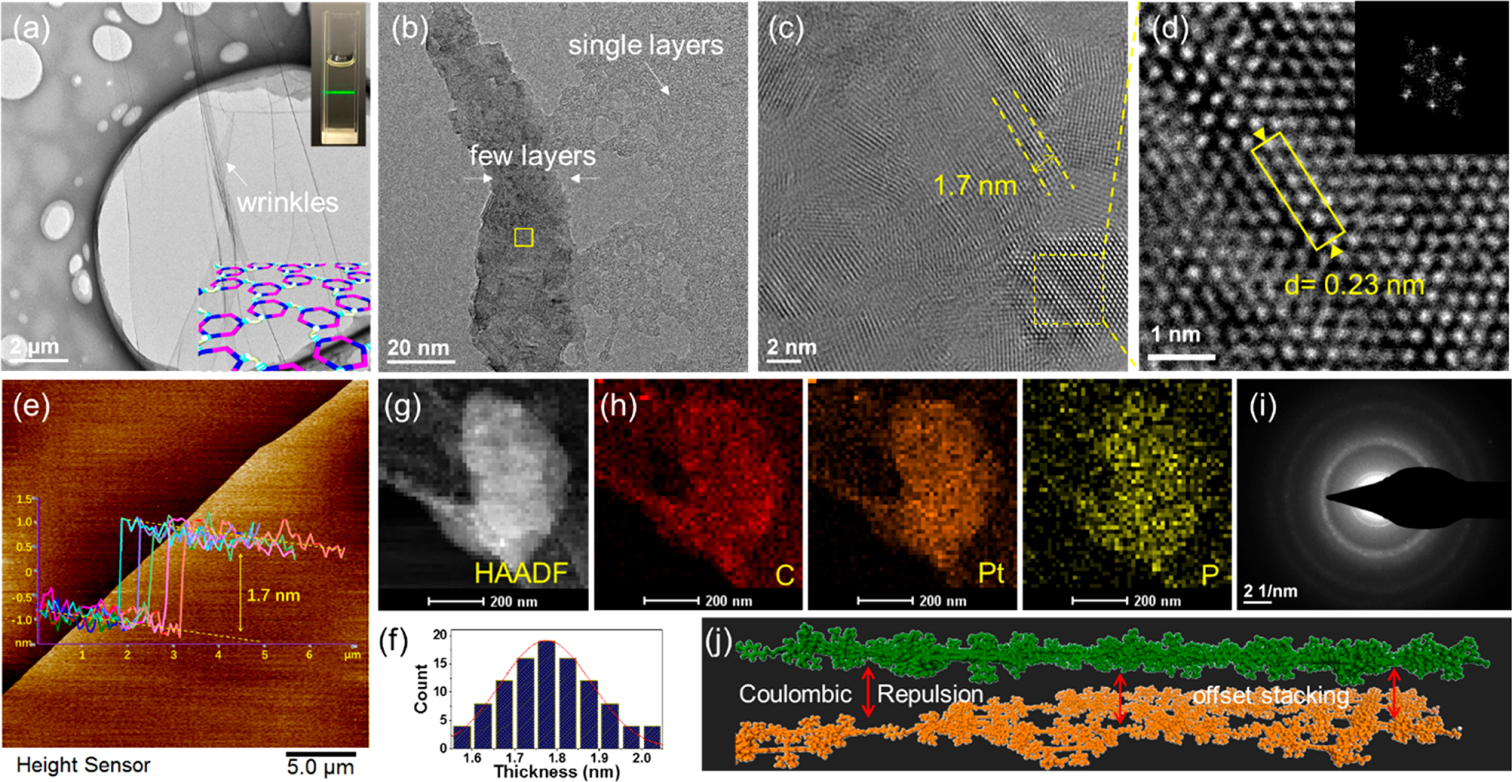
(a) TEM image of supramolecular polymer **5**, showing a 2D crumpled sheet image. The inset is a photograph of a polymer **5** dispersion in DMSO (∼0.1 mg mL^–1^), showing the Tyndall effect. (b) TEM image of polymer **5** showing the coexistence of film-like structures with different thickness. (c) Higher-magnification HR-TEM image of a polymer **5** sheet with moiré patterns. (d) Higher-magnification HR-TEM image with the corresponding FFT pattern, displaying a typical hexagonal atomic arrangement. (e) Perspective view of ScanAsyst mode AFM images by using the ScanAsyst-Fluid^+^ probe and corresponding height profiles of a sample prepared by drop coating a suspension of polymer **5** obtained after a prolong physical exfoliation by sonication in CH_2_Cl_2_ onto a mica sheet. (f) Histogram for statistics of the layer thickness for possible monolayers based on 103 different sites in AFM images. (g) HAADF-STEM image and corresponding EDX-mapping images (h) and SAED pattern (i) of polymer **5**. (j) Pictorial representation of layered films of polymer **5**, in which each metallacycle has positive charges on both ends. Units are shown in a stick mode with each layer in a different color and counterions omitted for clarity.

Stirring of a mixture of metallacycle **3** with methylene diphenyl diisocyanate **4** in tetrahydrofuran solution at room temperature, followed by precipitation with methanol, gave polymer **5** in 93% yield (Figure 1a and Scheme S2). Due to its highly cross-linked chemical structure, polymer **5** is insoluble in most solvents; however it can swell and finally dissolve in DMSO and DMF. An obvious Tyndall effect (Figure 2a inset) confirmed the dispersibility of polymer **5** in solution, which implies its excellent processability. Multiple peaks observed in both gel-permeation chromatography (GPC) and dynamic light scattering (DLS) analysis (Figure S8) provided evidence supporting a broad mass and size distribution of polymer **5** from tens up to hundreds of kDa. Typical absorption bands of the stretching vibration of C=O (1700 cm^–1^) and the hydrogen bonded N–H stretching vibration (broad bond at ∼3351 cm^–1^) were observed in the FTIR spectrum of polymer **5** (Figure S9), providing support for the formation of the polyurea material. The thermal decomposition temperature of polymer **5** was around 330 °C, indicating its high thermal stability (Figure S10). The ^31^P{^1^H} NMR spectrum of polymer **5** exhibits a broad singlet at 13.10 ppm, which was similar to that of hexagonal metallacycle **3** (Figures S2 and S11), implying the persistence of metallacyclic structures in supramolecular polymer **5**. In the ^1^H NMR spectra of **5**, the amine protons shifted from 5.33 ppm to 8.51 ppm and 7.41 ppm, respectively (Figures 1b and S2 and S12) because of the formation of polyurea. The signals corresponding to pyridyl moieties H_α_ and H_β_ and the aromatic protons H_1_, H_2_, H_3_, and H_4_ remained, indicating that the postassembly reaction does not perturb the hexagonal metallacycle scaffold. 2D COSY and ^1^H–^1^H NOESY NMR spectroscopy (Figures S13 and S14) also confirmed the entirety of the metallacycle scaffold in the polyurea polymer.

The morphology of supramolecular polymer **5** was investigated using transmission electron microscopy (TEM). By dripping the DMSO solutions of polymer **5** directly onto a TEM support, followed by freeze-drying under vacuum, a flexible film structure with overlays, roll-ups (Figures 2a and S15), or agglomerations (Figure S16) was formed.

Similar sheet-like structures were observed in atomic force microscopy (AFM) measurements (Figures 2e and S17) when spin coating DMSO solutions of polymer **5** (3 or 1 mg/mL) onto mica surfaces. The existence of multilayer structures with crossover and overlapping areas (Figures S18 and S19) or the coexistence of film-like structures with different transparencies (Figures 2b and S20) were also observed, thus supporting the existence of 2D layered structures in bulk solution. A scanning electron microscope (SEM) investigation further confirmed the sheet-like structures (Figure S21). We then employed energy dispersive X-ray spectroscopy (EDS) to characterize the elemental distribution. The elemental ratios for C, N, Pt, P, and S (Figure S22) were in accord with the composition expected for polymer **5**. The high-angle annular dark-field scanning transmission electron microscopy (HAADF-STEM) image further confirmed their membrane-like structure, as shown in Figure 2g. Most importantly, the elemental mapping images clearly disclosed a homogeneous distribution of C, Pt, P, etc., throughout the entire layer (Figures 2h and S22), verifying that these sheet structures were generated from supramolecular polymer **5**, other than adventitious impurities. Some sheets and films exhibit a quite low contrast against the background (Figures 2a and S18), suggesting that they are extremely thin, possibly a monolayer, although their thickness could not be estimated from TEM. Further evidence comes from the observation of nanosheets with a thickness around 1.7 nm from AFM (Figure S23), matching the thickness expected for a monolayer. Due to the high electron beam sensitivity, the transparent single sheets visibly degraded (Figure S24) during high-resolution TEM (HRTEM) measurements or selected area electron diffraction (SAED) analysis. This has been observed previously for ultrathin 2D materials.^43^ For the thicker multilayer films that can withstand bombardment by the electron beam, a layer-stacking morphology with atom-cloth-like patterns was observed under HRTEM imaging (Figures 2c and S25), and regular apparent light–dark lattice fringes with an average lattice spacing of ∼0.23 nm (Figures 2d and S26) were found in selected areas. This spacing matches well with that expected for Pt-containing structures. The hexagonal patterns as well as the hexagonal diffraction spots in the corresponding fast Fourier transform (FFT) patterns of a 6-fold symmetry which were different samples (Figures 2d and S26) indicate the existence of a hexagonal arrangement inside the structure. The presence of moiré fringes is evidence that the layered structure adopts slight interlayer offset stacking that has been observed with other layered 2D polymers and 2D COFs.^44^ SAED of these multilayers (Figures 2i and S27) was consistent with their polycrystalline, hexagonally ordered structure.

For comparative studies, samples were also prepared by means of the top-down sonication of the bulk solid of polymer **5**. Physical exfoliation of polymer **5** from the corresponding bulk solid was conducted by sonication in CH_2_Cl_2_. TEM images revealed unrolled and stacked polymer films with terraces appearing at the borders (Figure S28), indicating their multilayer structures. Sheet-like structures (Figure S30) were also found in a SEM investigation of polymer **5** exfoliation dispersions. EDS elemental mapping analysis associated with a TEM image (Figure S29) and SEM image (Figure S30), as well as surface composition investigated with X-ray photoelectron spectroscopy (XPS) (Figure S31), found the presence of elements that were the same as those of solution samples, suggesting again the formation of 2D sheet-like structures from polymer **5**. The SAED pattern of polymer **5** solids (Figure S34) displayed a series of polycrystalline rings with some discrete spots, in accordance with the results obtained from a polymer **5** solution sample. The wide-angle X-ray diffraction (WAXD) profile of polymer **5** showed one broad peak centered at approximately 2θ = 25° (Figure S36), suggesting its low crystallinity. This is due to the disordered random stacking nature of the freestanding sheets with few-layer numbers. The small-angle X-ray diffraction (SAXD) patterns of polymer **5** solids displayed diffraction peaks corresponding to about 3.6 nm (Figure S36), in agreement with the theoretical pore widths (Figure S38) calculated from the expected metallacycle units of the polymers. Small pores with a diameter of about 3.3 nm (Figure S35), which is characteristic of the metallacycle pores, were also observed in TEM measurements, confirming the formation of 2D polymers with the expected honeycomb-like backbones.

HRTEM images of supramolecular polymer **5** solid multilayer films from different areas showed fine lattice structures and moiré fringes (Figure S32). Moreover, periodic linear moiré patterns with a periodic distance of 1.7 nm were observed (Figures 2c and S33), in agreement with periodic distance along one direction of layered stacking, suggesting the retention of some vertical stacking. The formation of extremely thin layers by physical exfoliation was observable in AFM measurements (Figures 2e and S37). Cross-sectional analysis indicated that the films were very flat and uniform. A statistical study of the monolayer thickness suggests a mean average height of about 1.7 nm (Figure 2f), which is similar to that predicted by calculation.

The design and synthesis of 2D polymers is a challenging task,^45^ especially for preparing 2D polymers in homogeneous solution.^46−49^ However, with this system, a free-standing, single-to few-layer 2D polymer film was prepared without any preorganization of building blocks on solid surfaces or interfaces. The semiempirical method PM7^50^ in the program MOPAC2016^51^ was used to optimize the structure of the positively charged metallacycle during the formation of the polymer. We calculated the binding energies of two metallacycle rings in simple stacking models (a, b, and c in Figure S38) to examine how the metallacycle assembles into the polymer. The simulation showed that the interactions between two metallacycle rings were negligible, especially when the interlayer distance was larger than 3 nm, while weak repulsions between them blocked the extension of the metallacycle along its vertical axis (Figure S38). Therefore, computational simulations suggested that the three reactive groups attached to the vertex of a hexagonal metallacycle may prefer growth as an extension of its network along the horizontal axes due to electrostatic repulsion. Figure 2j shows the simulated polymer in its 2D layered offset stacking, in which the positive charges on its skeleton keep each polymer sheet separated from one another (Figure S39). Based on the above findings, the positive charges on the metallacycle might be one of the most important factors controlling the formation of 2D structures.

### Stimuli-Responsive Properties of 2D Metallacycle-Cored Polymer **5**

The 2D metallacycle-cored polymer **5** was expected to display stimuli-responsive switchable properties due to the dynamic nature of metal–ligand bonds. As detected by both AFM (Figure S40) and TEM (Figure S41) measurements, the regular planar network of **5** dispersed upon addition of Bu_4_NCl, forming an irregular nanostructure. The diameter of the sample of the polymer decreased in the DLS experiment (Figure S42) upon addition of Bu_4_NCl, providing further evidence in support of the stimuli-responsiveness of this system.

**Figure 3:**
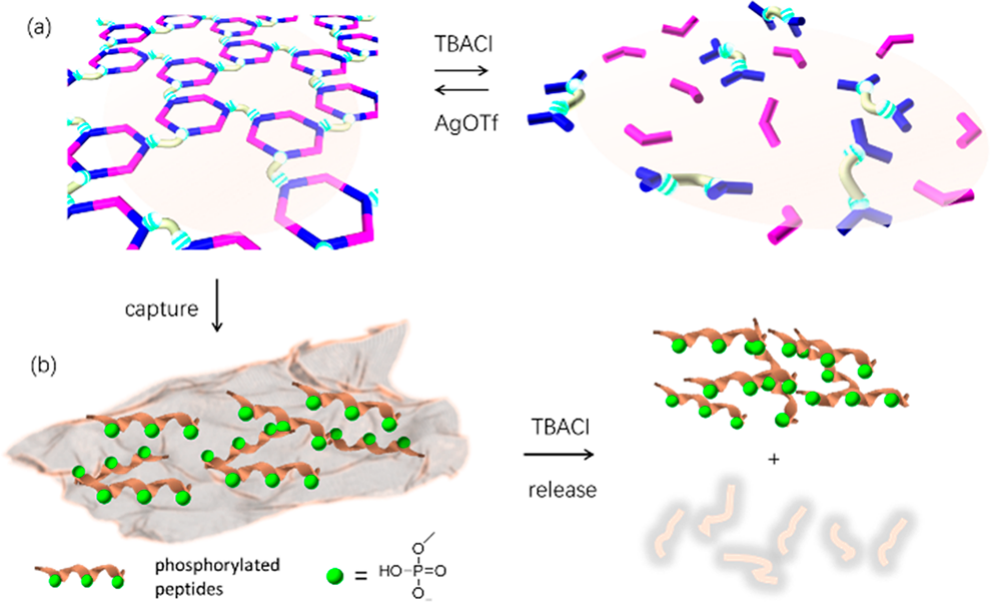
Stimuli-responsive properties of 2D metallacycle-cored polymer **5**. (a) Schematic representation of stimuli-responsive disassembly and reassembly of 2D supramolecular polymer **5** and (b) the corresponding binding/release toward PPs modulated by external stimuli.

*In situ* multinuclear NMR (^1^H and ^31^P) investigations of the polymer provided evidence that this stimuli-responsive phenomenon was a consequence of the halide-induced disassembly and reassembly of the hexagonal metallacycles (Figure S43). After the addition of Bu_4_NCl, the proton resonances of hexagonal metallacycles disappeared, along with an increase in resonances from free the ligand, indicating complete disassembly of the hexagonal metallacycle. The original resonances of **5** in the ^1^H and ^31^P NMR spectra were restored upon the addition of AgOTf to the mixture, demonstrating the quantitative reassembly of the polymer. The stimuli-responsive properties of polymer **5** offered flexibility in controlling the behavior of polymer **5** using external stimuli (Figure 3a), which would further determine differential binding toward anions and PPs (see below) and make it possible to modulate adsorption and release of PPs (Figure 3b).

### Binding and Affinity Properties of 2D Metallacycle-Cored Polymer **5** toward Anions and Standard PPs

**Figure 4:**
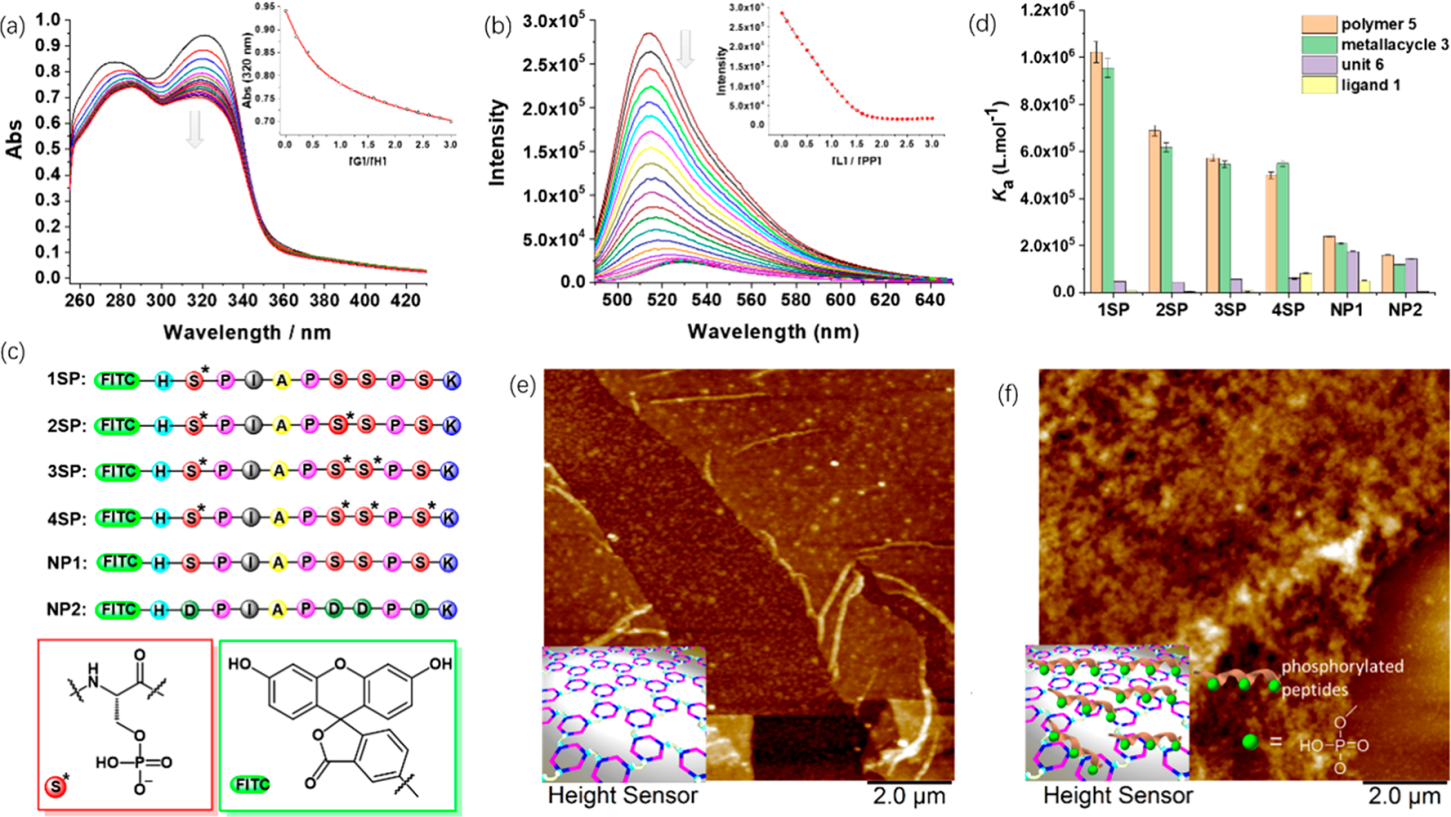
(a) UV–vis titration of polymer **5** (1.0 × 10^–5^ mol·L^–1^, based on the dipyridine units) with PO_4_^3–^ in DMSO solution at 20 °C with tetrabutylammonium (Bu_4_N^+^) as cations. The absorbance values from 320 nm were fitted globally to a 1:1 binding model (assuming one dipyridine unit of polymer **5** as one host, inset figure), giving an apparent binding constant of 4.4 × 10^5^ M^–1^. (b) Typical fluorescence spectra of N-terminal fluorescein-labeled serine phosphorylated peptide 3SP (1.0 × 10^–6^ mol·L^–1^) upon addition of different equivalents of polymer **5** (concentrations are calculated based on the dipyridine units) in aqueous solution at 20 °C and the fluorescent intensity change (inset figure) of peptide hosts upon the additions of guests. [L]/[PP] is an abbreviation of the molar ratio of guest to host. (c) Amino acid sequences of serine phosphorylated peptides 1SP–4SP and nonphosphorylated peptides NP1 and NP2 studied in this work. (d) Comparison of apparent *K*_a_ of polymer **5**, metallacycle **3**, unit **6**, and ligand **1** with various peptides. Data are from *n* = 3 independent experiments and are presented as mean ± s.e.m. (e) AFM images before and after (f) treatment with serine monophosphorylated peptides 1SP.

Given that polymer **5** is positively charged and contains urea hydrogen bond donors, we explored whether this system could bind anions in solution. We found that upon addition of one equivalent (concentration calculated relative to the stoichiometry of the dipyridine units) of TBAH_2_PO_4_, the polymer precipitated from solution and its ^1^H NMR resonances became undetectable (Figure S44). Meanwhile, chemical shifts were observed in both ^1^H and ^31^P NMR spectra of TBAH_2_PO_4_ with the addition of a small quantity of polymer **5** (Figure S45), indicating affinity between **5** and H_2_PO_4_^–^. To further investigate the anion binding properties of polymer **5**, UV titration experiments, a typical and widely adopted method for calculating the association constant (*K*_a_) in host–guest chemistry, were conducted to evaluate the apparent binding affinity of **5** with various anionic guests (i.e., H_2_PO_4_^–^, HPO_4_^2–^, PO_4_^3–^, HCO_3_^–^, and CH_3_COO^–^, Figures S46–S50). As shown in Figure 4a, upon slow addition of PO_4_^3–^ into a solution of polymer **5** (10 μM, calculated based on the concentration of dipyridine units within the polymer), a gradual decrease in absorption intensity at approximately 320 nm was observed. Polymer **5** was found to bind hydrogen phosphates and phosphate strongly and selectively. The apparent association constant of **5** with phosphate (*K*_a_ = 4.4 × 10^5^ M^–1^) was about 54 times higher than that with acetate (*K*_a_ = 8.1 × 10^3^ M^–1^) (Table S1), demonstrating the phosphate selectivity of the polymer. Anion complexation studies were also conducted with metallacycle **3** (Figures S51–S55). Interestingly, affinities for the tested anions with metallacycle **3** were observed comparable to those with polymer **5** (Figure S57 and Table S1), showing that the metallacycle skeleton is critical for binding anionic guests and that the electrostatic interactions may be the main driving force for anion complexation in these systems. To prove that the disassembly of the metallacycle backbone will result in a loss of binding affinity toward phosphate, the phosphate affinity of ligand **1** was also studied in a UV/vis titration experiment. As expected, negligible absorption change was observed upon the addition of H_2_PO_4_^–^ into the solution of ligand **1** (Figure S56), indicating that ligand **1** display no capacity for binding phosphate. The very different binding capacity of polymer **5** and ligand **1** toward phosphate makes it possible to modulate the affinity of polymer **5** using external stimuli to control polymer formation.

Four N-terminal fluorescein-labeled model peptides with identical amino acid sequences differing only in the number of phosphate groups (serine mono-, di-, tri-, and tetra-PPs, abbreviated as 1SP–4SP, Figure 4c) were prepared to study the binding affinity of polymer **5** for PPs. Fluorescence titration experiments, in which *K*_a_ values were elucidated according to intensity changes in the maximum emission peak, were conducted with the four model peptides and different compounds including polymer **5**, metallacycle **3**, and related compounds ligand **1** and unit **6** (Figure S40). It was found that the fluorescence of the model PPs was efficiently quenched upon addition of polymer **5** (as an example, the titrations with 3SP are shown in Figure 4b). In all cases, the calculated apparent association constants between polymer **5** and PPs were in the 10^5^–10^6^ M^–1^ range (Table S2), indicating that polymer **5** was an ideal affinity candidate for binding PPs from mono- to multiply charged PPs. For 1SP to 4SP, a similar turn-off response was observed with metallacycle **3**. By comparison, the fluorescence intensity of the PPs did not show a significant response toward ligand **1** and unit **6** (Figures S58–S61). These results suggested again that the electrostatic interaction is the main driving force in the interaction between polymer **5** and PPs, and there is a significant difference in PPs’ binding affinity between polymer **5** before and after external stimuli.

In complex samples such as cell lysates, several other protein functional groups could compete for affinity enrichment. Therefore, we performed control experiments in which nonphosphorylated peptide with identical amino acid sequences NP1 and another acidic peptide NP2 with Ser residues substituted by Asp were used (Figure 4c). Under the same conditions, for both NP1 and NP2, only slight decreases in fluorescence intensity were observed (Figures S62 and S63), with the calculated association constants being much lower than those of the PPs (Figure 4d and Table S2), thus indicating satisfactory discrimination ability of the polymer between PPs and NPs.

AFM measurements were then conducted to directly visualize the interaction between the PPs and polymer **5**. As shown in Figure 4e,f, upon adding a solution of 1SP followed by washing with water, the polymer film expanded with the morphology changing from thin film to thick cross-linked fiber. No 1SP was retained on the surface of the blank substrate (without the coating of polymer) after washing (Figure S64). Consistent morphological changes were observed when the polymer film was treated with other multiply charged PPs from 2SP to 4SP (Figures S65–S67). Under the same conditions, no obvious change of film morphology and thickness was observed upon interaction with nonphosphorylated peptides NP1 and NP2 (Figures S68 and S69). These results further confirmed satisfactory selectivity and high adsorption capacities of polymer **5** toward PPs.

Theoretical calculations of the possible binding modes and binding energies between polymer **5** (metallacycle **3** was considered as the monomer unit of polymer **5**) and different model peptides suggested that the skew-crossing mode and skew-clinging mode are the dominant modes of interaction (Figure S70 and Table S3). The calculated binding energy of 1SP was slightly larger than those of control model peptides Asp-P and Glu-P, in which the phosphorylated serine group was replaced by two other common negatively charged amino acid Asp or Glu groups, respectively (Figure S71 and Table S4). This result demonstrated the discrimination ability of the polymer between PPs and other low isoelectric point peptides. The binding energy increased with the number of phosphate groups from 1SP to 4SP (Figures S72–S74 and Table S5).

### Strategy for the Enrichment of PPs using 2D Polymer **5**

**Figure 5:**
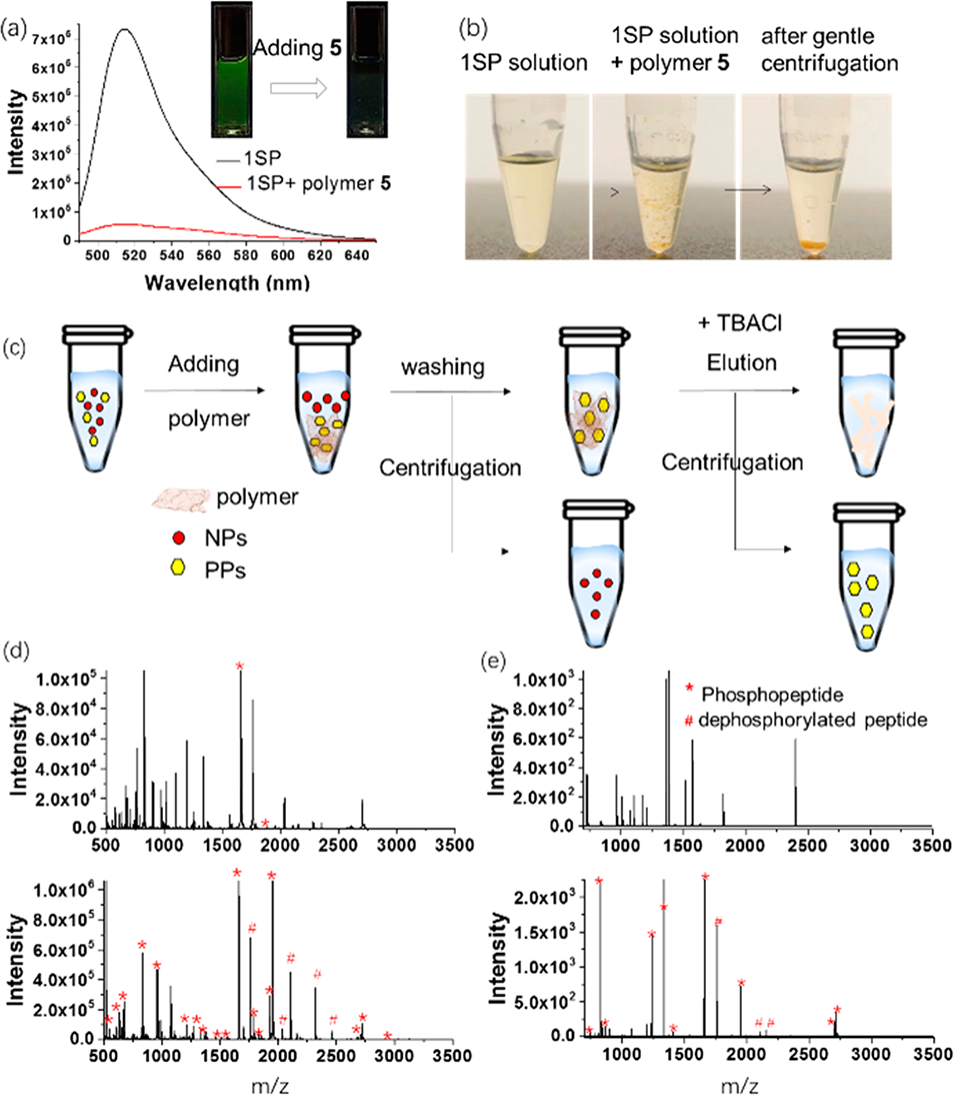
Enrichment and separation of PPs in model protein samples. (a) Emission intensity of 1SP before and after capture of polymer **5**. Inset is the photograph under UV. (b) Dispersity analysis of 1SP before (left) and after (middle) adding of polymer **5** and after gentle centrifugation (right). (c) Schematic illustration of PPs’ enrichment strategy using polymer **5**. (d) MALDI-TOF mass spectra of α-casein tryptic digest (1 pmol). Direct analysis (top) after enrichment by polymer **5** (bottom, *phosphopeptide; ^#^dephosphorylated peptide). Detailed information about peptide sequences and phosphorylation sites is shown in Supporting Information Table S6. (e) MALDI-TOF mass spectra of tryptic digests of α-casein and BSA at molar ratios of 1:10. Direct analysis (top) after enrichment by polymer **5** (bottom, *phosphopeptide; ^#^dephosphorylated peptide).

The above results illustrate the potential of the newly designed 2D supramolecular polymer **5** to capture PPs. To validate this point, we evaluated the PP enrichment and separation properties of polymer **5** using a phase extraction method with 1SP as a model phosphoprotein sample. After adding one equivalent of polymer **5** (Figure 5a), the green emission of the 1SP solution decreased (91% quench) and subsequently recovered (90% recovery) upon the addition of Bu_4_NCl (Figure S75), indicating the effective capture and release of 1SP. As shown in Figure 5b, the clear solution of 1SP immediately becomes a milky, light yellow suspension after addition of polymer **5** due to the interaction between polymer **5** and 1SP and the low solubility of polymer **5** in water. The yellow polymer precipitates with a bulky fibrous morphology (Figure S76) and can be easily collected by centrifugation. It is worth highlighting that 1SP cannot be precipitated out of solution by the interaction between 1SP and macrocycle **3**, although macrocycle **3** displayed affinity and selectivity toward phosphorylated peptides similar to polymer **5**, indicating the superiority of polymer **5** as a PP affinity material. The high binding affinity of polymer **5** with PPs makes it simple to remove NPs that interfere with the analysis by simple washing with water. The remarkable difference in binding affinity of polymer **5** and ligand **1** with PPs enables the efficient isolation of PPs and subsequent MS detection. Thus, as illustrated in Figure 5c, an efficient protocol via a two-step elution procedure under mild operating conditions was developed for the enrichment and isolation of PPs.

### PPs’ Enrichment from Model Protein Samples

We applied the strategy first to the isolation of PPs from the tryptic digest of a standard phosphoprotein (bovine α-casein). A direct matrix-assisted laser desorption ionization (MALDI) time-of-flight (TOF) mass spectrum of α-casein is illustrated in Figure 5d (top). Signals of nonphosphorylated peaks dominated the spectrum, and phosphopeptides signals were severely suppressed by those of nonphosphorylated peptides. Nevertheless, after the treatment by the polymer **5** as affinity material, most of the nonphosphorylated peptides had been removed and nine monophosphopeptides and 13 multiphosphopeptides (Figure 5d (bottom) and Table S6) were observed after enrichment. As a control, only one phosphopeptide signal was detected after treatment by metallacycle **3**, and no signal could be detected after treatment with unit **6** or ligand **1** (Figure S81). Moreover, fewer phosphopeptide signals were detected after enrichment when using commercially available titanium dioxide as an affinity material (Figure S77). The data from three independent experiments show the high degree of reproducibility of the polymer enrichment method (Figure S79).

To further simulate the complex physiological environment, semicomplex samples including tryptic digests of α-casein mixed with different molar ratios (i.e., 1:10, 1:20, 1:100, and 1:500) of bovine serum albumin (BSA) as a model interfering protein were tested. As shown in Figure 5e, when the molar ratio of α-casein and BSA was 1:10, five monophosphopeptides and five multiphosphopeptides were selectively enriched and could be clearly detected. The satisfactory separation capabilities of the polymer **5** toward PPs were still maintained when the molar ratio of casein to BSA was increased to 1:100 and even to 1:500 (Figure S78). As a comparison, dioxide showed a preference for monophosphopeptides, leading to less detectable multiphosphopeptides in the analysis of those semicomplex samples (Figure S80).

The concentrations of phosphopeptides bound to the affinity materials are crucial for the identification of diagnostic markers owing to their low abundance in the lysates of biosamples. Therefore, the detection sensitivity for PPs was measured by using low-concentration α-casein tryptic digest. For polymer **5**, two phosphopeptides were detected with an S/N ratio of 8 and 4 for 2 fmol of α-casein tryptic digests (Figure S82). The lower detection limit of polymer **5** may be attributed to the high amount of immobilized PPs and the strong binding interaction between phosphopeptides and polymer **5**. This result indicates that the polymer **5** has a high detection sensitivity for phosphopeptides.

**Figure 6:**
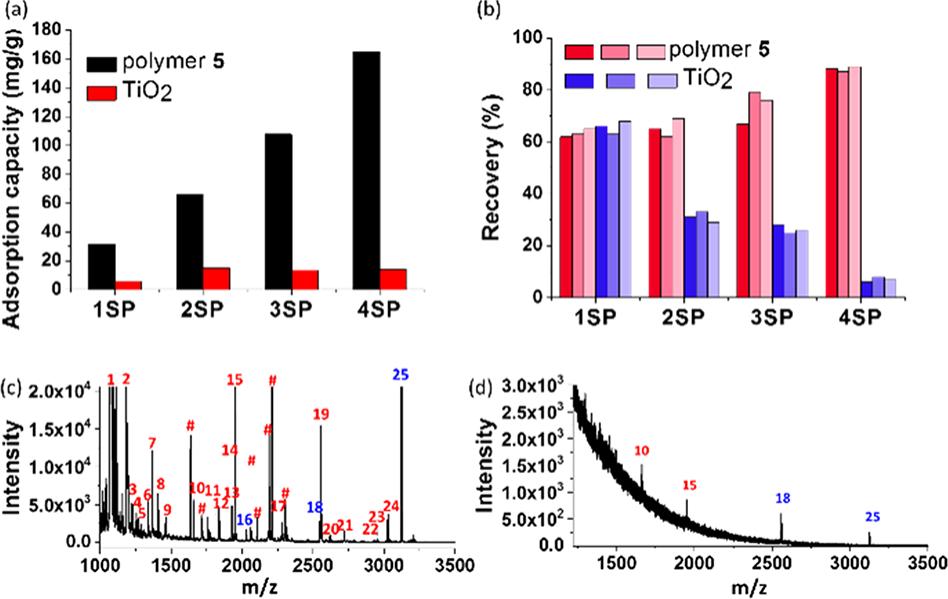
Adsorption capacities, recovery, and specific enrichment of PPs from nonfat milk. (a) Comparison of adsorption capacities of polymer **5** (black columns) and commercially available TiO_2_ (red columns) toward 1SP–4SP. (b) Comparison of recovery of polymer **5** and commercially available TiO_2_ based enrichment methods toward 1SP–4SP, obtained from three parallel MS measurements. (c) MALDI-TOF mass spectra of tryptic digests of the nonfat milk after enrichment by polymer **5** (red, α-casein; blue, β-casein, ^#^dephosphorylated peptide) and commercially available TiO_2_ (d).

The enrichment capacity of polymer **5** toward PPs was then investigated using standard peptides 1SP–4SP. Different amounts of SPs in a fixed volume were incubated with a fixed amount of polymer material until maximal loading was reached. As illustrated in Figures 6a and S83, the adsorption capacity of the material increased substantially with the number of phosphates in the SPs. In comparison, titanium dioxide displayed much weaker and unbiased adsorption capacities^52^ toward various SPs (Figure S84). For example, the enrichment capacity of polymer **5** toward 4SP was about 165 mg/g, nearly 10 times higher than that of titanium dioxide (17 mg/g).^52^ The improved adsorption capacities of polymer **5** may be attributed to the unrestricted mass transfer and improved accessibility of SPs toward the binding sites in the homogeneous reaction-based enrichment.

The enrichment recovery of phosphopeptides (defined as the ratio of released PPs to the total PPs, involved in both binding and releasing processes of the PPs) was investigated using nonphosphorylated peptides as a control. Fixed amounts of standard phosphopeptides 1SP to 4SP were treated with polymer **5**, and the eluate was mixed with the same amount of NP1 or NP2 (Figures S85–S86). As shown in Figure 6b, the enrichment recovery of monophosphopeptide 1SP from polymer **5** was about 65%. As for multiply charged phosphopeptides 2SP–4SP, the recoveries obtained using polymer **5** were increased to about 88% with the increase of charge number. The bound multiply charged phosphopeptides could be disassociated from the polymer surface relatively easily owing to the controllable binding behavior of polymer **5**, leading to high recoveries for PPs. Thus, a convenient separation procedure, satisfactory recovery, high adsorption capacities, and high detection sensitivity make polymer **5** suitable for enrichment and analysis of various phosphopeptides.

### Application in Highly Specific Enrichment of PPs from Nonfat Milk

Encouraged by the above results, to further examine the selectivity and effectiveness of polymer **5** in the capture of low-abundance phosphopeptides from complex samples, a tryptic digest mixture of nonfat milk, which contains abundant proteins, including phosphoproteins such as α-casein and β-casein, was prepared and tested. Nonphosphopeptide signals dominated the mass spectrum obtained from direct analysis of the tryptic digest of proteins in nonfat milk, and no MS signal intensity of phosphopeptide was detected (Figure S87). As shown in Figure 6c, after enrichment with polymer **5**, a total of 25 phosphopeptides including 16 monophosphopeptides and nine multiphosphopeptides, corresponding to phosphopeptides of α-casein and β-casein, could be identified, while fewer phosphopeptides could be detected after enrichment with dioxide (Figure 6d). Detailed information on the 25 phosphopeptides extracted from the tryptic digest of nonfat bovine milk is provided in Table S7. These results demonstrate the utility of this method and the potential of polymer **5** to be used in the analysis of biological samples in the future.

The potential of supramolecular polymers as affinity materials to specifically and comprehensively isolate PPs from a complex sample has never been evaluated previously. Although the use of supramolecular coordination complexes as sensors, catalysts, and biomedicines has been widely reported,^31−39^ this is the first report on the application of these systems in proteomics. The utility of soluble supramolecular polymers allows for fast homogeneous interaction with PPs under mild conditions, which may be beneficial to analysis of unstable PPs. In this study, the 2D supramolecular polymer **5** was used to capture PPs owing to its morphology and solubility, as well as controllable affinity upon external stimuli. Our two-step approach based on polymer **5** enriching PPs results in comparable performance to those of other reported two-dimensional materials (Table S8).^53,54^ It is worth highlighting that a number of TiO_2_- and IMAC-based protocols have been highly optimized over the years, enabling very high specificity and sensitivity for PP enrichment and MS analysis.^55,56^ In contrast, despite the relatively immature status of enrichment workflows employing our supramolecular polymeric materials, results obtained thus far are comparable with those from optimized methods employing TiO_2_ microcolumns with similar detection strategies (MALDI-TOF).^57^ This indicates the scope for high performance of our material, particularly with continued development of enrichment workflows. We envision that further optimization of enrichment protocols employing our polymeric material and the combination of these with high performance detection platforms will result in enhanced enrichment results. Considering the potential for mild elution conditions by decomposition of the polymer, we also envision a possible role for the material in the analysis of acid-labile noncanonical phosphorylation (e.g., histidine phosphorylation), which is particularly widespread in prokaryotes.^58^

## Conclusions

In summary, by linking the hexagonal metallacycle **3** via a reaction between amine and isocyanate, a free-standing, metallacycle cross-linked, single-monomer-thick 2D stimuli-responsive supramolecular polymer in solution was successfully prepared. The multiple positive charges on the metallacycle skeleton can keep each individual polymer sheet separated from one another by electrostatic repulsion during synthesis, resulting in a robust and readily transferable 2D metallacycle-cored supramolecular polymer **5**. Furthermore, the metallacycle backbone endows the resultant polymer with a high propensity for binding phosphate-containing anions and peptides. The different binding capacities of polymer **5** toward PPs before and after stimuli allow it to be used as a tunable and specific affinity material for the enrichment of phosphopeptides. High enrichment capacities, detection sensitivity, and excellent recovery are achieved with diverse PPs. Polymer **5** may also be used to identify low-abundance phosphopeptides from complex biological samples. We believe that this work provides the basis for the rational design of next-generation affinity materials for comprehensive phosphoproteome research based upon supramolecular structures.

## Supporting Information Available

Experimental section, synthetic overview, a full set of characterization data, and a complete set of binding and enrichment studies (PDF)
