# Supplementary material for: A Two-Dimensional Metallacycle Cross-Linked Switchable Polymer for Fast and Highly Efficient Phosphorylated Peptide Enrichment
Source: J Am Chem Soc. 2021 May 27;143(22):8295–304. doi: 10.1021/jacs.0c12904 (PMC8193630; doi:10.1021/jacs.0c12904)
Supplement: Supplementary file 1 — ja0c12904_si_001.pdf [file ja0c12904_si_001.pdf]

A Two-Dimensional Metallacycle Cross-Linked Switchable Polymer for Fast and Highly Efficient Phosphorylated Peptide Enrichment

Li-Jun Chen,[†] Sean J. Humphrey,[‡] Jun-Long Zhu,[‖] Fan-Fan Zhu,[‖] Xu-Qing Wang,[‖] Xiang Wang,[⊥] Jin Wen,[⊥,#,*] Hai-Bo Yang,[‖,*] and Philip A. Gale[†,§,*]

Supporting Information

Supporting Information....................1

Experimental Procedures....................2

1.1 General Information....................2

1.2 Synthesis of amino functionalized metallacycle **3**....................3

1.2 Synthesis and characterization of 2D supramolecular polymer **5**....................5

Results and Discussion....................7

2.1 Characterization of amino functionalized metallacycle **3**....................7

2.2 Characterization of 2D supramolecular polymer **5**....................10

2.3 Stimuli-responsive disassembly and reassembly of 2D supramolecular polymer **5**....................27

2.4 Binding and Affinity Properties of Polymer **5** towards anions and PPs....................29

2.4.1 Binding investigation of 2D supramolecular polymer **5** towards various anions....................29

2.4.2 Binding and affinity investigation of 2D supramolecular polymer **5** towards model PPs....................35

2.4.3 Theoretical calculation of the intermolecular binding energies between metallacycle **3** and different model PPs....................41

2.5. Application of polymer **5** in Selective Enrichment of Phosphopeptides....................45

2.5.1 Enrichment of phosphopeptides from model protein samples....................45

2.5.2 Evaluation of the detection sensitivity, enrichment capacity, and enrichment recovery of polymer **5** in phosphopeptide enrichment....................49

2.6 Application in highly specific enrichment of phosphopeptides from non-fat milk....................52

Experimental Procedures

1.1 General Information.

All reagents were of analytical purity and used without further treatment. TLC analyses were performed on silica-gel plates, and flash chromatography was conducted using silica-gel column packages. All solvents were dried according to standard procedures and all of them were degassed under N_2 for 30 minutes before use. All air-sensitive reactions were carried out under argon atmosphere. A series of N-terminus fluorescein-labelled mono-, di-, tri-, tetra-serine phosphorylated peptides (1SP—4SP), and two non-modified peptides (NP1 and NP2) were purchased from China-Peptides Corp. (Shanghai, China) with high purity (> 99.5%). ^1H NMR, ^{31}P NMR and ^{13}C NMR spectra were recorded on Bruker 400 MHz Spectrometer (^1H: 400 MHz; ^{31}P: 161.9 MHz; ^{13}C: 100 MHz) or Bruker 300 MHz Spectrometer (^1H: 300 MHz; ^{31}P: 121.4 MHz) at 298 K. The ^1H NMR chemical shifts are reported relative to residual solvent signals. Coupling constants (*J*) are denoted in Hz and chemical shifts (δ) in ppm. Multiplicities are denoted as follows: s = singlet, d = doublet, m = multiplet, br = broad. Electrospray ionization (ESI) mass spectra were recorded with a Waters Synapt G2 mass spectrometer. UV−vis spectra were recorded in a quartz cell (light path 10 mm) on a Cary 4000 UV-Visible spectrophotometer. Steady-state fluorescence spectra were recorded in a conventional quartz cell (light path 10 mm) on a HORIBA Scientific FluoroMax-4 spectrophotometer. Powder X-ray diffraction (PXRD) patterns were collected on a Rigaku Ultima IV diffractometer using Cu Kα radiation at 35 kV and 25 mA in the 2θ angle range of 0.5-8º using a step size of 0.02° and at a scanning speed of 10° min^{-1}. The samples were prepared by filling the holder with the dry powder. Scanning electron microscopy (SEM) images of the solutions were obtained using an S-4800 (Hitachi Ltd.) with an accelerating voltage of 3.0-10.0 kV. Energy dispersive X-ray spectroscopy (EDS) measurements were performed using an S-4800 (Hitachi Ltd.) with a voltage of 10.0 kV. Transmission electron microscopy (TEM) images, high resolution TEM (HRTEM) and selected area electron diffraction (SAED) images were recorded on a Tecnai G^2 F30 (FEI Ltd.). Samples were deposited on a holey carbon coated TEM-grid (300 mesh Cu grid, Pacific Grid Tech, USA). XPS spectra were recorded on a Thermo Fisher ESCALAB 250Xi system with Al Kα radiation, operated at 150 W. AFM images were obtained on a Dimension Fast Dimension icon MultiMode8 (Bruker), using ScanAsyst mode under ambient condition. Scanasyst-Fluid$^+$ probes were used for scan and samples were prepared by dropping solutions onto a mica sheet. Thermogravimetric analyses (TGA) were performed on a Netzsch STA 449 F3 instrument. The sample was heated from room temperature to 973 K with the heating rate 10 K·min^{-1} under a flow of nitrogen (10 mL·min^{-1}). Gel permeation chromatography equipped with a Shimadzu Prominence GPC System (Shimadzu Inc.) and a Shimadzu RID-20A refractive index detector. Two Shodex columns (Shimadzu Inc.) with effective molecular weight ranges were 400 – 7,000 and 500 – 400,000, respectively, were connected in series. DMF was used as an eluent at a flow rate of 1.0 mL/min at 40 ºC. Calibration was done with 10 monodisperse polystyrene standards in the range 1.15 kDa to 2.38 MDa. Polymer concentration in the solution (1.0 mg/mL or 3.0 mg/mL), injection volume 20 µL. DMF and sample solutions were filtered over a filter with a pore size of 0.25 µm or 0.45 µm (PTFE, Millex-HN 33 mm Syringes Filters, Titan, China).

Preparation of tryptic digests of standard proteins. For digestion standard proteins, 1 mg α-casein protein was dissolved with 1 mL 50 mM ammonium bicarbonate (NH_4HCO_3) solution. Then, trypsin was used to digest the protein with an enzyme to protein ratio of 1:30 at 37 °C for 16 h.

BSA (2 mg) was dissolved with 100 µL of 6 M urea in 50 mM NH_4HCO_3 solution. 2 µL of a 50 mM DL-dithiothreitol (DTT) was added to the BSA protein solution and the resulting solution was stored at 56 °C for 45 min to reduce the disulfide bonds of proteins. Subsequently, 5 µL of 50mM iodoacetamide (IAA) was added and the mixture was incubated in the dark for 30 min at room temperature. Finally, the mixture was diluted 10-fold with NH_4HCO_3 (50 mM) and incubated at 37 °C for 16 h with trypsin at the mass ratio of enzyme to protein of 1:30 (w/w). Semi-complex samples were tryptic digest of α-casein with different ratios (i.e., 1:10, 1:20, 1:100 and 1:500) of digest of BSA.

Preparation of tryptic digests of proteins extracted from non-fat milk. To analyze real samples, non-fat milk (30 µL) was dissolved in 1 mL of NH_4HCO_3 (50 mM), and this solution was centrifuged at 16 000 rpm for 10 min. The supernatant solution was then denatured at 100 °C for 10 min. A solution of 10 µL of the supernatant solution in 50 µL of 6 M urea was reduced with 2 µL of 50 mM DTT at 56 °C for 1 h and alkylated with 2 µL of 50 mM IAA in the dark for 30 min at 37 °C. Subsequently, the supernatant solution was digested at 37 °C for 16 h with trypsin at a 1:30 (w/w) enzyme/substrate ratio. Finally, the digest mixture was diluted 50-fold with 50 mM NH_4HCO_3 and stored at −20 °C before MS analysis.

Enrichment protocols of PPs with polymer 5 and TiO_2. For the case of enrichment of PPs with polymer **5**, tryptic digests of α-casein, the mixture with BSA, or proteins extracted from non-fat milk were dissolved in 30% ACN-H_2O (100 µL); then polymer **5** (2 mM in DMSO solution, 5 µL) was added, resulting in a precipitate. After removing the supernatant, the precipitate was washed three times with ultra-pure water (50 µL × 3) to remove the non-specifically adsorbed peptides. The captured phosphopeptides were eluted by adding Bu_4NCl (5 mM in 50% ACN-H_2O, 20 µL) with shaking and characterized with MALDI-TOF MS.

Enrichment of PPs with commercially available TiO_2 was based on previous reports[36] with minor modifications. Briefly, the TiO_2 material (2 mg) was washed with loading buffer (70% ACN-H_2O, and 5% TFA) and then suspended in corresponding loading buffer. The tryptic peptide mixture was dried, re-dissolved in 20 µL loading buffer and TiO_2 was then added, and the mixture was incubated at room temperature for 30 min. After removing the supernatant, the bound peptides were washed with 40 µL of loading buffer twice and eluted with 40 µL of 0.1% ammonium hydroxide (pH: 10.82). The elution fraction was immediately acidified with 1% formic acid.

Evaluation of the enrichment capacity towards PPs. To measure the enrichment capacity of polymer **5** toward PPs, serine mono-di-, tri- and tetra-PPs (1SP–4SP) at different concentrations were prepared and loaded onto 1mM polymer **5**, respectively. After the washing and eluting procedure, the eluted fraction was analyzed with MALDI-TOF MS. The adsorption capacity was measured as the

highest PP to adsorbing material ratio at which no PP signals were observed in the MS spectra. The experiments were repeated once. The detailed figures are shown in Supplementary Fig. S57.

For TiO_2, fixed concentrations of 1SP-4SP at different volumes were dissolved in loading buffer (70% ACN-H_2O, and 5% TFA) and loaded into 1 mg affinity materials, respectively. After the washing and eluting procedure, the eluted fraction was analyzed with MALDI-TOF MS. The adsorption capacity was measured as the highest PP to adsorbing material ratio at which no PP signals were observed in the MS spectra. The experiments were repeated once. The detailed figures are shown in Supplementary Fig. S58.

Determination of enrichment recovery of PPs. 1SP-4SP, NP1 and NP2 were used as model peptide samples to determine the enrichment recovery of both polymer **5** and the TiO_2-based methods. A certain amount of standard phosphopeptide (1SP-4SP, 0.5 pmol) was enriched with the capture materials according to the procedure above. The eluted solution was mixed with the same amount of nonphosphopeptide NP1 (0.5 pmol, for 1SP) or NP2 (0.5 pmol, for 2SP-4SP), and the mixed peptides were analyzed by MALDI-TOF MS. The recovery of standard phosphopeptide (1SP-4SP) was calculated by the MS intensity ratio of the peptide (1SP-4SP) divided by the nonphosphopeptide NP1 or NP2.

Mass Spectrometry Analysis. All MALDI-TOF MS experiments were performed in reflector positive mode on Autoflex^{TM} Speed MALDI-TOF/TOF mass spectrometer (Bruker Daltonics Inc) with a pulsed Nd/YAG laser at 355 nm. Matrix DHB was dissolved in 70% ACN-H_2O containing 1% H_3PO_4 (20 mg mL^{-1}). A 2.0 μL aliquot of the eluate and 2.0 μL of DHB matrix were mixed together before 0.5 μL sequentially dropped onto the MALDI plate for MS analysis.

1.2 Synthesis of amino functionalized metallacycle 3.

Scheme S1. Self-Assembly of Supramolecular [3 + 3] *tris*-amino Metallacycle **3** from Dipyridine Ligand **1** and Diplatinum Acceptor **2**.

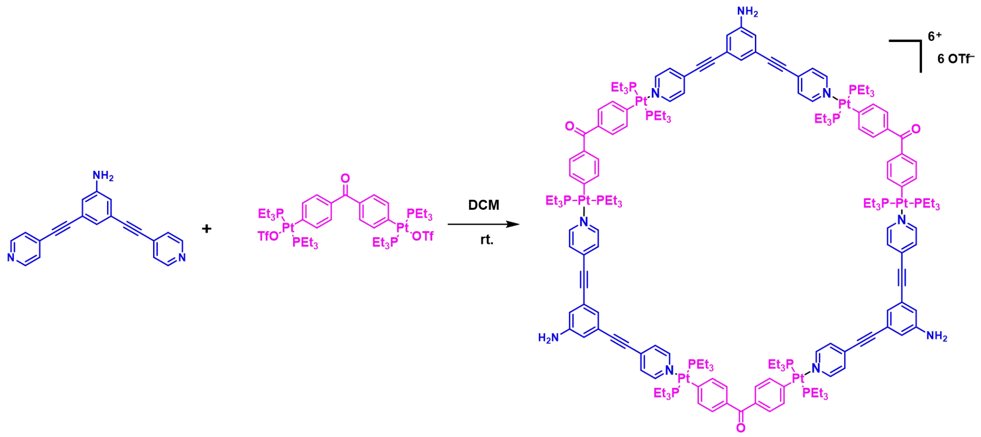

Synthesis of **3:** The dipyridyl donor ligand **1** (20.0 mg, 67.7 μmol) and the organoplatinum 120° acceptor **2** (90.8 mg, 67.7 μmol) were weighed accurately into a round bottomed flask. Methanol (20mL) was added and the reaction solution was then stirred at room temperature for 10 h to yield a homogeneous solution. The light-yellow product **3** (110.0 mg, > 99%) was collected after removing the solvent under vacuum. IR (neat): ν/cm^{-1} 2963, 2922, 2853, 2208, 1635, 1607, 1574, 1456, 1417, 1380, 1255, 1222, 1147, 1063, 1028, 924, 841, 758, 731, 675. ^{1}H NMR (acetone-d_6, 400 MHz): δ 9.07 (d, J = 8.0 Hz, 12H, H_α-Py), 7.91 (d, J = 8.0 Hz, 12H, H_β-Py), 7.70 (d, J = 12.0 Hz, 12H, H_3), 7.57 (d, J = 12.0 Hz, 12H, H_4), 7.13 (s, 3H, H_1), 7.10 (s, 6H, H_2), 5.33 (br, 2H, NH_2), 2.04-2.06 (m, 72H, -$CH_2$$CH_3$), 1.16-1.30 (m, 108H, -$CH_2$$CH_3$); ^{31}P{^{1}H} NMR (acetone-d_6, 161.9 MHz): δ 12.86 (s, $^1J_{Pt\text{-}P}$ = 2660.0 Hz). ^{13}C NMR (methanol-d_4, 100 MHz): δ 198.96, 153.67, 150.41, 137.58, 136.04, 134.55, 130.55, 130.44, 124.91, 123.65, 120.30, 119.64, 99.03, 85.89, 13.69, 13.52, 13.35, 7.90. ESI-TOF-MS: *m/z* calcd for $C_{173}H_{243}F_6N_9O_9P_{12}Pt_6S_2$ ([M − 4OTf$^-$]$^{4+}$): 1078.2, found: 1078.2; calcd for $C_{172}H_{243}F_3N_9O_6P_{12}Pt_6S$ ([M − 5OTf$^-$]$^{5+}$): 832.8, found: 832.8.

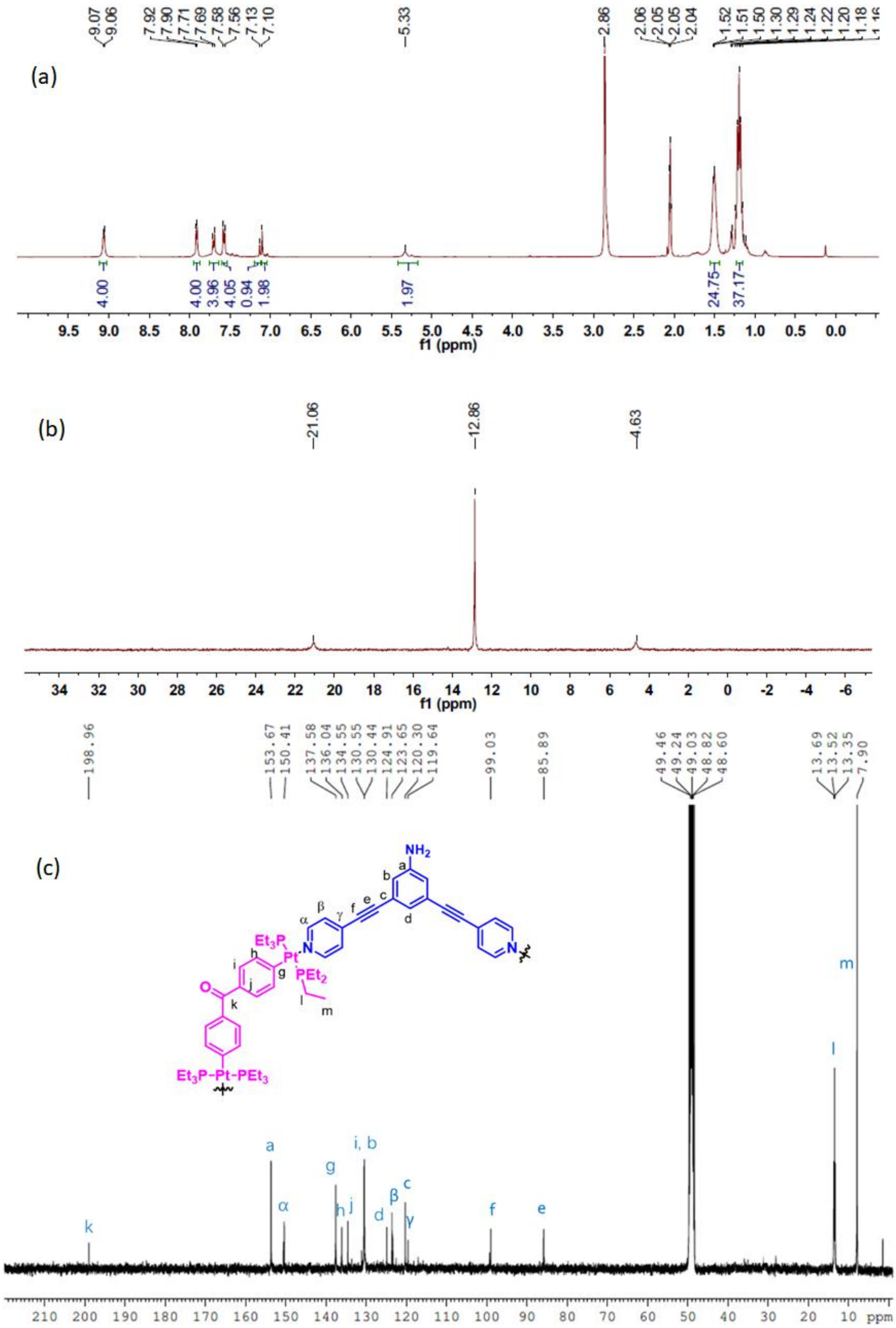

Figure S1. (a) ^{1}H NMR spectrum (400 MHz, acetone-*d*$_6$, 298 K), (b) ^{31}P NMR spectrum (161.9 MHz, acetone-*d*$_6$, 298 K), (c) ^{13}C NMR spectrum (100 MHz, methanol-*d*$_4$, 298 K) of metallacycle **3**.

1.2 Synthesis and characterization of 2D supramolecular polymer 5.

Scheme S2. Synthesis of metallacycle-cored supramolecular polymer through post-Assembly polycondensation of supramolecular [3 + 3] *tris*-amino Metallacycle **3** with methylene diphenyl diisocyanate **4**.

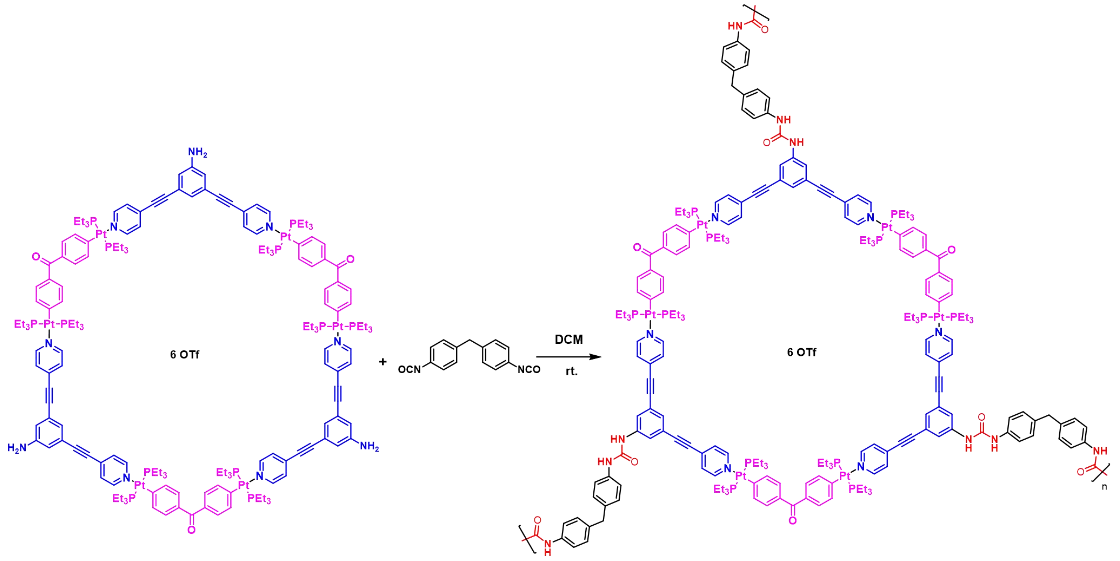

Synthesis of polymer **5:** Methylene diphenyl diisocyanate **4** (10 mg, 39.9 µmol) was added to amino metallacycle **3** (130.8 mg, 26.6 µmol) solution (dry THF, 200 mL) and the mixture was stirred for three hours at 50 °C under a nitrogen atmosphere. THF was then removed and the product was purified by multiple slow precipitation in methanol and dried *in vacuo*. The product was isolated as a light brown colored solid (12.76 g, 93% yield. IR (neat): v/cm^{-1} 3351, 2965, 2922, 2878, 2209, 1700, 1635, 1606, 1573, 1535, 1492, 1454, 1415, 1380, 1306, 1256, 1222, 1150, 1064, 1028, 954, 924, 839, 758, 731, 707, 675. ^{1}H NMR (400 MHz; DMSO-d_6) δ 8.86 (br, 4H, H_α-Py), 8.51 (br, 1H, H_8), 7.88 (br, 4H, H_β-Py), 7.56 (br, 4H, H_3), 7.47 (br, 4H, H_4), 7.41 (br, 1H, H_9), 7.33 (d, J=8.0 Hz, 4H, H_5), 7.17 (br, 1H, H_1), 7.09 (d, J=8.0 Hz, 4H, H_6), 6.95 (br, 2H, H_2), 1.33 (m, 24H, -CH_2CH$_3$), 1.06-1.10 (m, 36H, -CH$_2$$CH_3$); ^{13}C NMR (100 MHz; DMSO-d_6) δ 198.15, 195.15, 152.41, 150.05, 137.61, 135.94, 134.91, 132.37, 129.00, 128.86, 125.44, 122.29, 119.09, 118.87, 118.31, 115.01, 114.02, 97.20, 86.33, 34.40, 12.11, 11.95, 11.78, 7.30. ^{31}P NMR (161.9 MHz, DMSO-d_6): 13.10 (s, J_{Pt-P}= 2634.1 Hz).

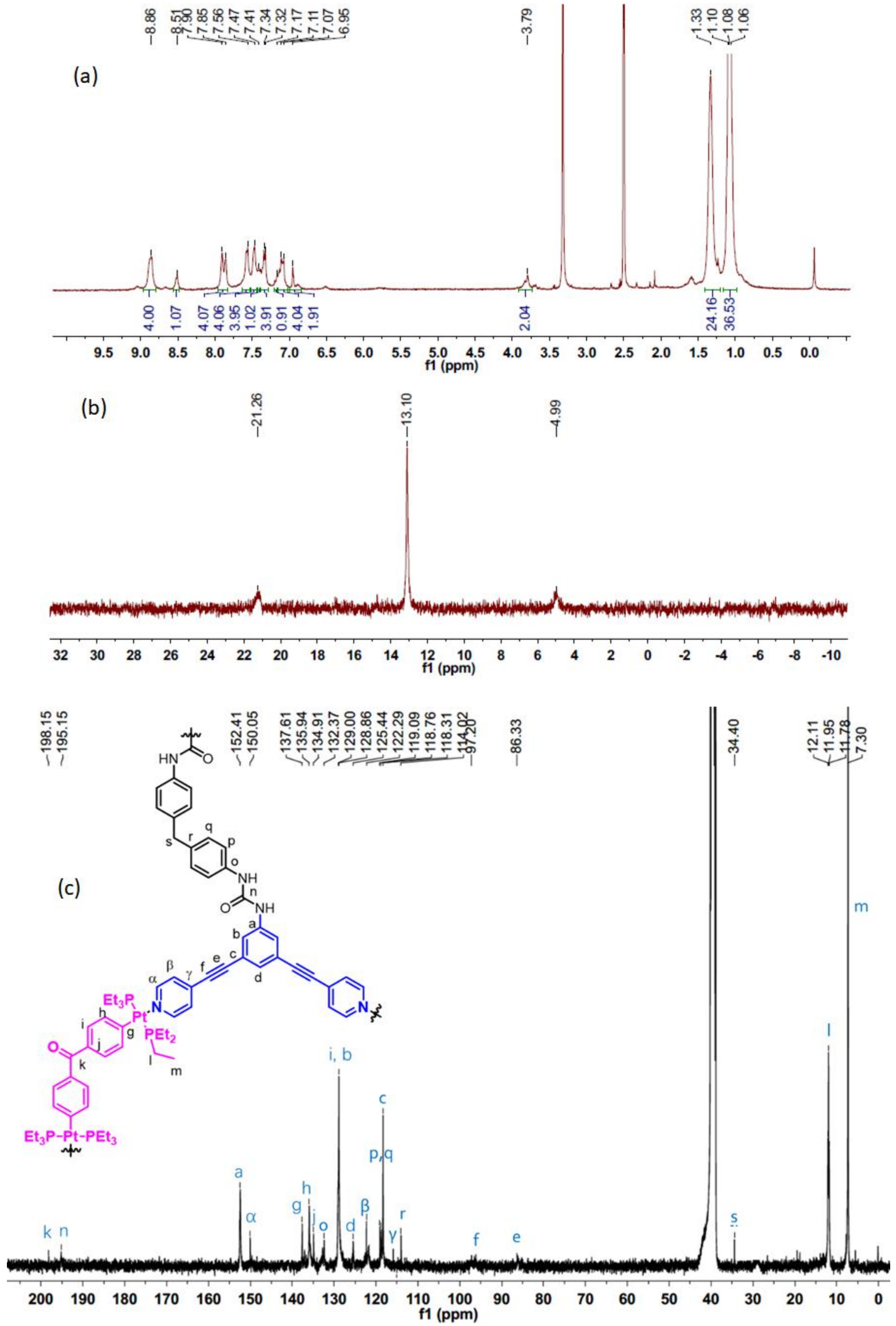

Figure S2. (a) ^{1}H NMR spectrum (400 MHz, DMSO-*d*6, 298 K), (b) ^{31}P NMR spectrum (161.9 MHz, DMSO-*d*6, 298 K), (c) 13C NMR spectrum (100 MHz, DMSO-*d*6, 298 K) of polymer **5**.

Results and Discussion

2.1 Characterization of amino functionalized metallacycle 3.

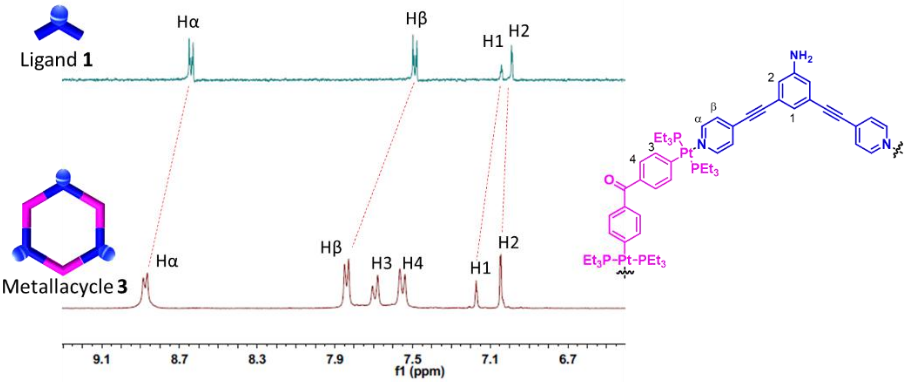

Figure S3. The partial ^{1}H NMR spectra (400 MHz, 298 K) of 120º donor ligand **1** (top) and self-assembled *tris*-amino metallacycle **3** (bottom).

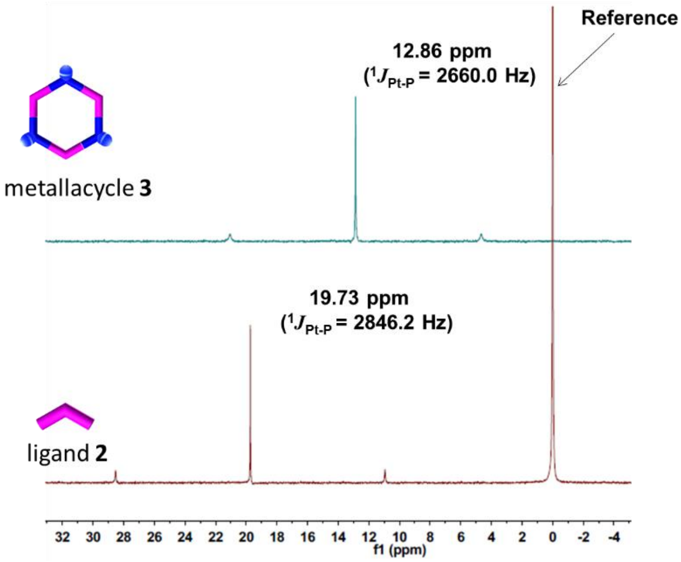

Figure S4. The ^{31}P NMR spectra (161.9 MHz, 298 K) of acceptor **2** (bottom), and self-assembled *tris*-amino metallacycle **3** (top) and ligand **2** (bottom).

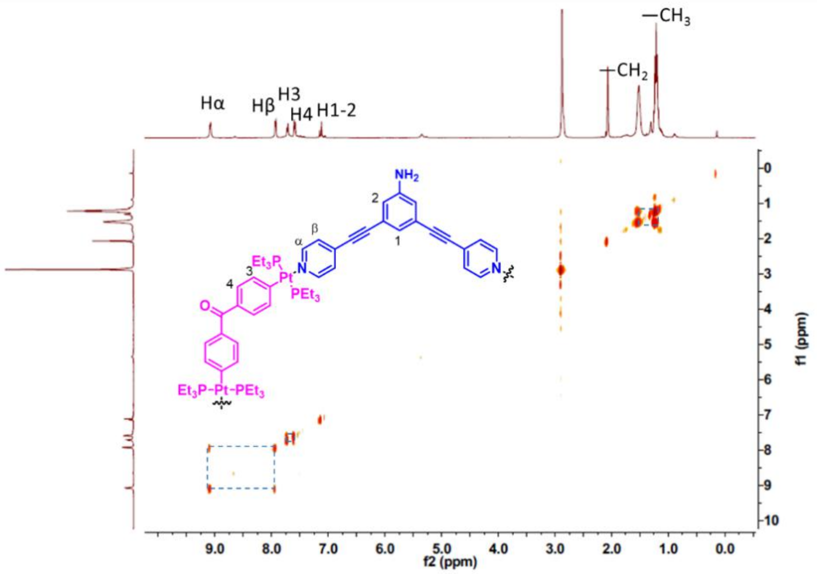

Figure S5. 2D COSY NMR (400 MHz, acetone-d_6, 298 K) spectrum of *tris*-amino metallacycle **3**.

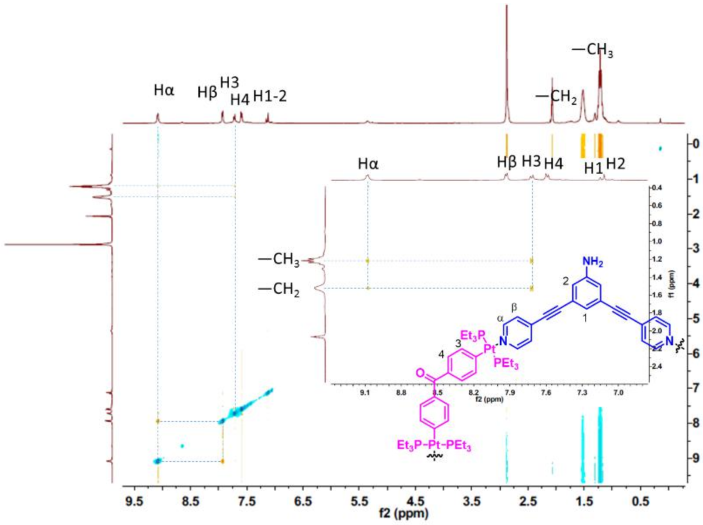

Figure S6. 2D NOESY NMR (500 MHz, acetone-d_6, 298 K) spectrum of *tris*-amino metallacycle **3**.

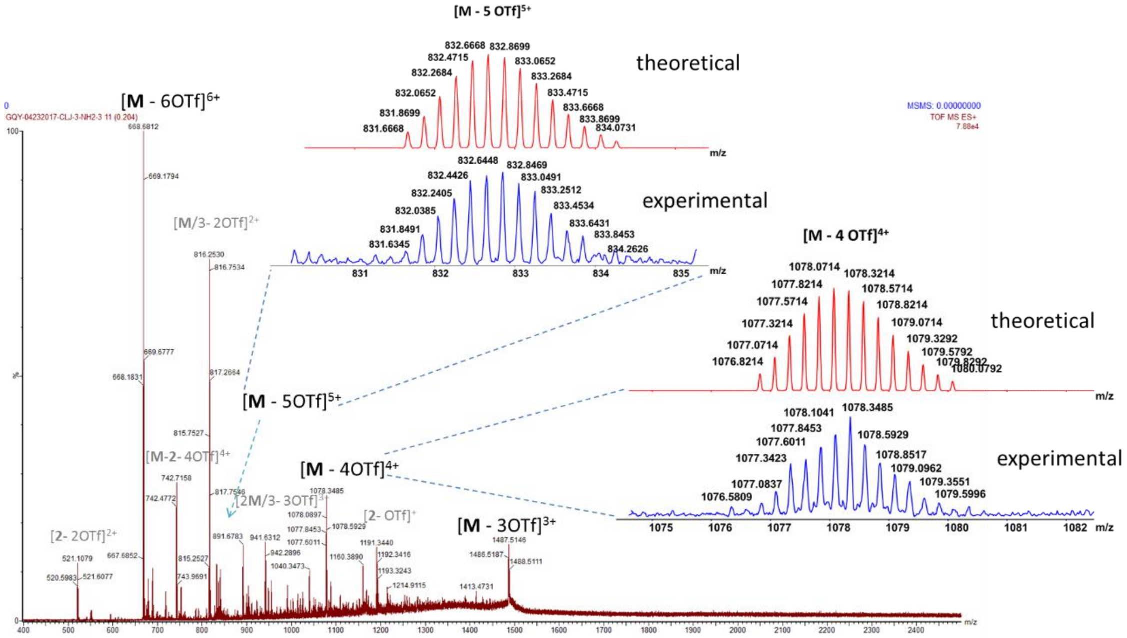

Figure S7. Full spectra of ESI-TOF-MS spectrum of amino metallacycle **3** and related theoretical and experimental isotopic distributions.

2.2 Characterization of 2D supramolecular polymer 5.

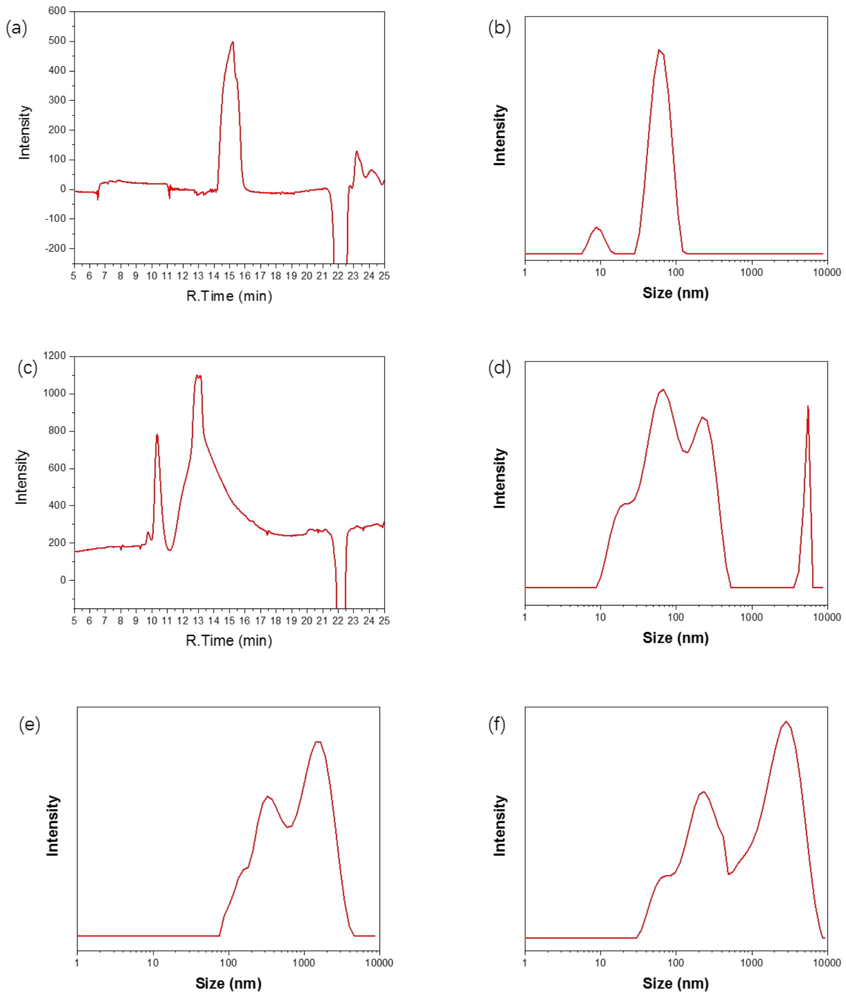

Figure S8. GPC trace (a) and DLS meaurment (b) of polymer **5** in DMF(1.0 mg/mL) after filtrating twice with a syringe filter (0.25 μm/33 mm), and GPC trace (c) and DLS meaurment (d) of polymer **5** in DMF(3.0 mg/mL) after filtrating once with a syringe filter (0.45 μm/33 mm), and DLS meaurment of polymer **5** in DMF(3.0 mg/mL, e) and in DMSO (3.0 mg/mL, f) without filtration. Although the measured molecular weight distribution and hydrodynamic diameter may not accurately represent the molar mass distribution and size due to the anisotropic shape of dispersed polymer **5** films, the measurement at least provides a reasonable estimate of the molar mass and size of polymer **5** films.

GPC analysis obtained after a filtration process (filtrating twice by using a syringe filter (0.25 μm/33 mm) before injection) (DMF, room temperature) revealed a material with M_n and M_w values of 22720 and 23071, respectively (Figure S6a). However, the appearance of molecular weight distribution measured herein might not be representative for the entire product since the filtration process may remove substance with high molecular weight. This was evidenced by the GPC result obtained by using sample with relatively higher concentration (3.0 mg/mL) through a filtration process by using a syringe filter with larger pore size (0.45 μm/33 mm). Under this condition, GPC chromatograms showed two peaks, including a peak at lower retention time with molecular weight distribution extends up to 225 kDa and an irregular peak at higher retention time with molecular weight around 20 kDa (Figure S6c). The obtained high molecular mass exceeded the resolution limit of GPC and therefore they are likely to be larger objects in solution. Note that the retention time also depends on molecular shape such as linear and branched chain. Thus, the appearance of molecular weight distribution measured herein might not the same as the actual molecular weight distribution, since the calibration was done with polystyrene standards which is linear, while polymer **5** is cross-linked film, as well as the fact that the actual concentration was impossible to be measured duo to the pretest filtration process. Base on above test results, although we could not estimate the actual molecular weight distribution of polymer **5**, it is true that the molecular weight has a broad distribution from 10 kDa to 230 kDa, indicating that the obtained polymer product may contains a mixture of crosslinked network with different extent of linkage. The broad size distribution of polymer **5** were also evidenced by DLS analysis of samples used in GPC measurements (Figure S6b, d), as well as samples without filtration (Figure S6e, f), in which multiple size distribution peaks were observed. Therefore, it might be difficult to provide an actual averaged molar mass of polymer **5** in our case.

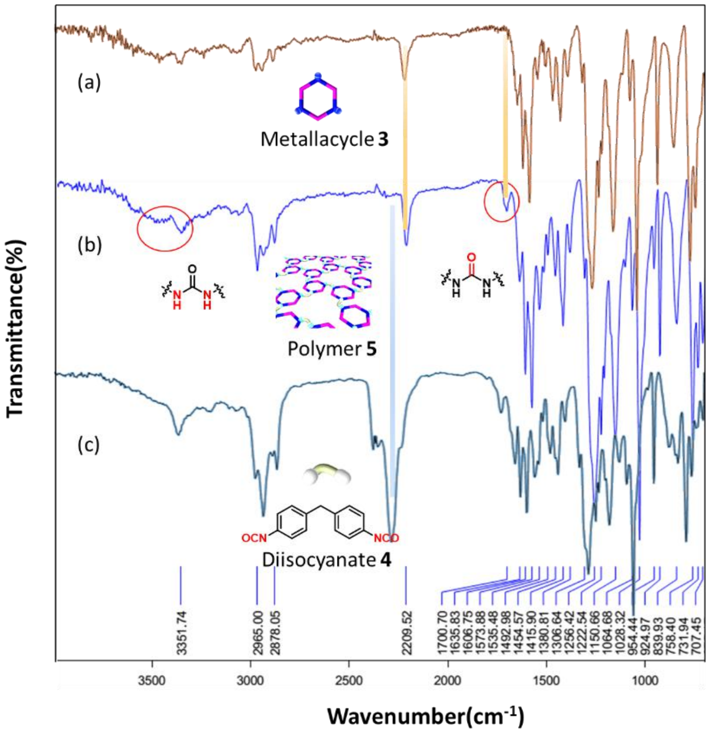

Figure S9. FT-IR spectra of metallacycle **3** (a), polymer **5** (b) and methylene diphenyl diisocyanate **4** (c).

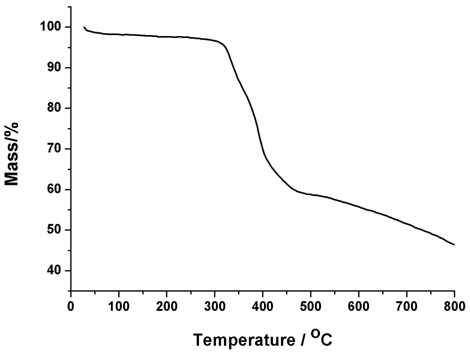

Figure S10. Thermogravimetric (TG) curves of supramolecular polymer **5**.

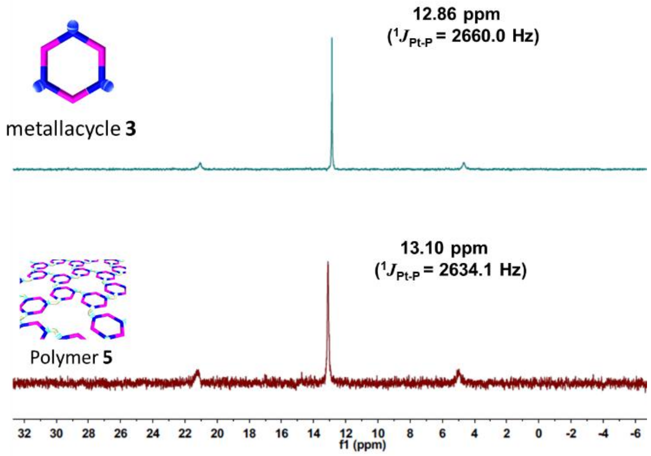

Figure S11. The ^{31}P NMR spectra (161.9 MHz, 298 K) of metallacycle **3** (top), polymer **5** (bottom).

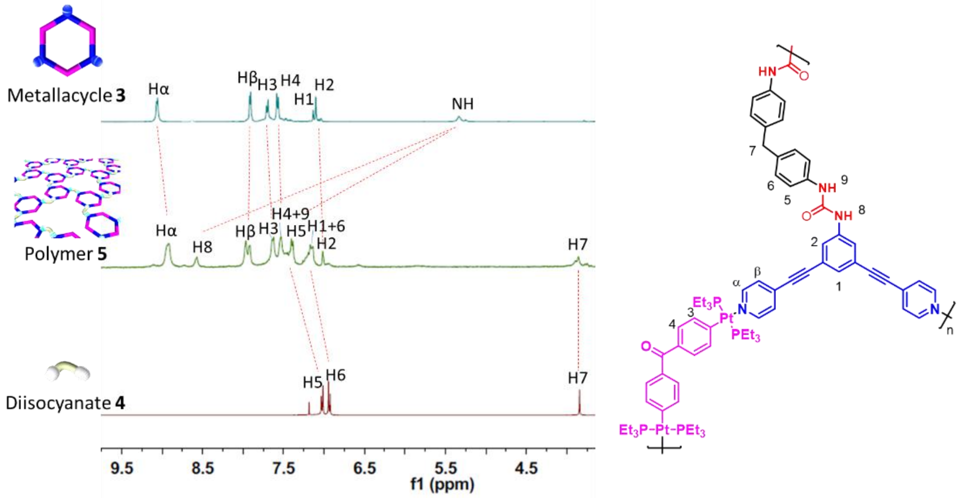

Figure S12. The partial ^{1}H NMR spectra (400 MHz, 298 K) of metallacycle **3** (top), polymer **5** (middle) and methylene diphenyl diisocyanate **4** (bottom).

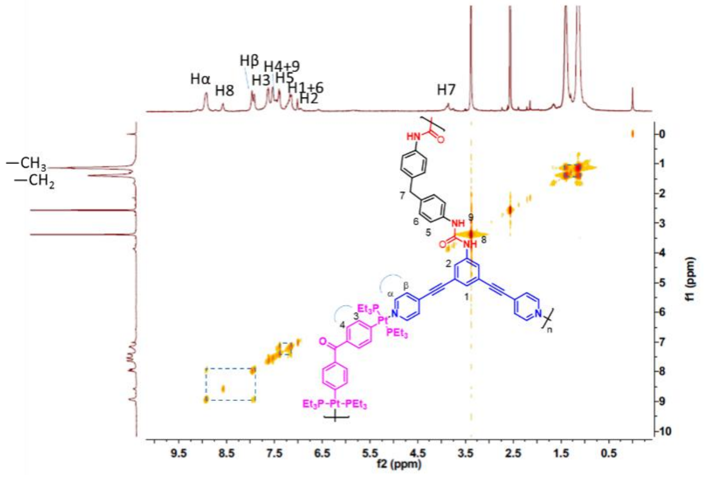

Figure S13. 2D COSY NMR (400 MHz, DMSO-d_6, 298 K) spectrum of supramolecular polymer **5**.

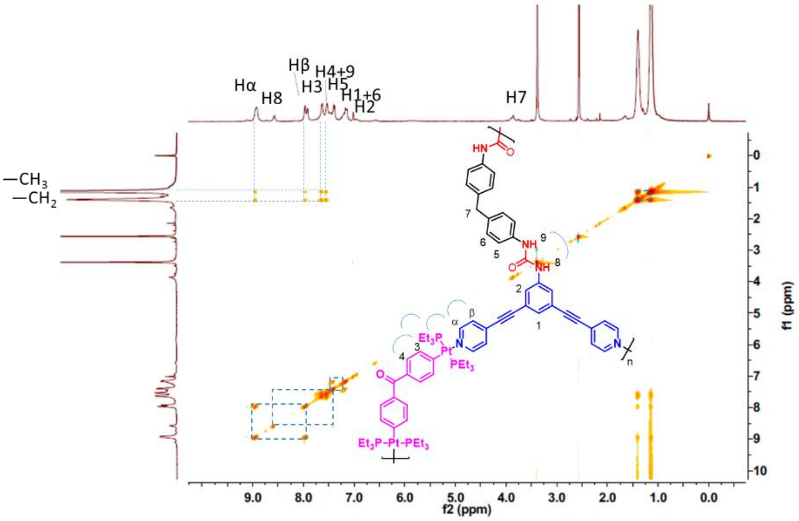

Figure S14. 2D NOESY NMR (400 MHz, DMSO-d_6, 298 K) spectrum of supramolecular polymer **5**.

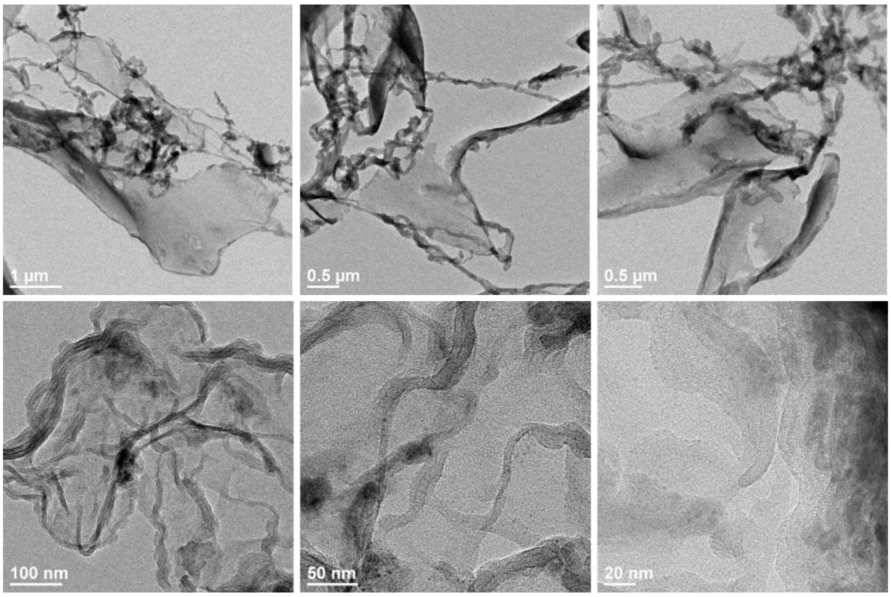

Figure S15. TEM images of supramolecular polymer **5** with different scale bars, showing flexible film structures with overlays, roll-ups. Samples were prepared by dropping DMSO solution of polymer **5** (3 mg/mL) directly unto the copper grid, followed by freeze drying under vacuum.

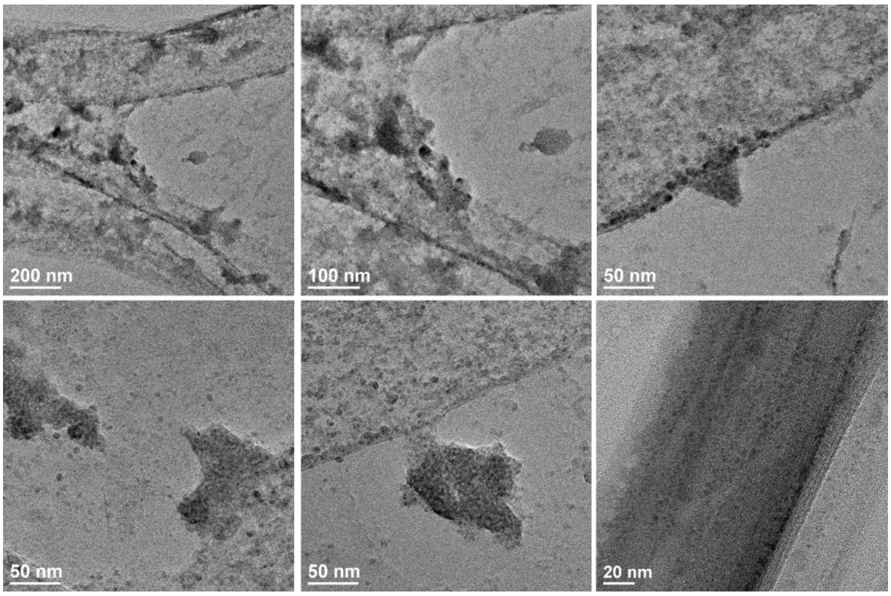

Figure S16. TEM images of supramolecular polymer **5** with different scale bars, showing film structures with agglomerations. Samples were prepared by dropping DMSO solution of polymer **5** (3 mg/mL) directly unto the copper grid, followed by frozen dry under vacuum.

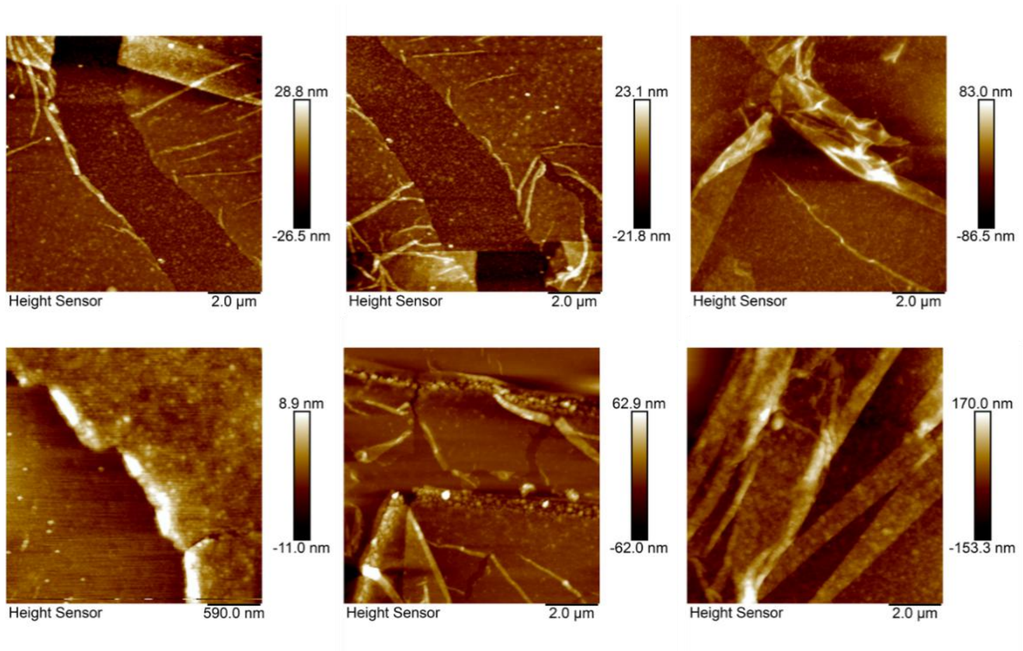

Figure S17. AFM images and height profiles of 2D polymer **5.** Perspective-view of ScanAsyst mode AFM images by using ScanAsyst-Fluid⁺ probe of samples prepared by spin coating DMSO solution of polymer **5** (3 mg/mL) onto a mica sheet. The thickness of those membrane like structures displayed a very wide range from ~4 nm to ~100 nm, suggesting sheet stacks rather than simple nanosheets under this condition.

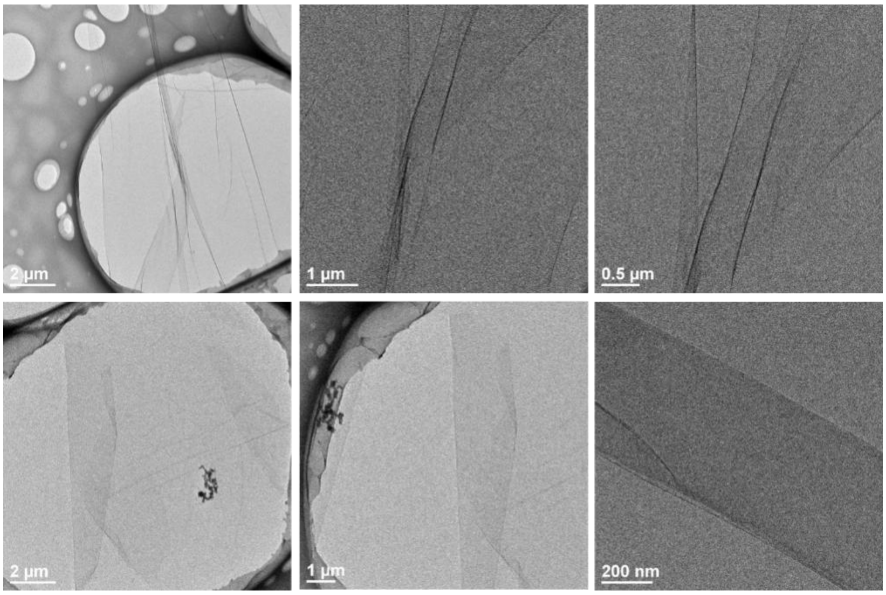

Figure S18. TEM images of supramolecular polymer **5** with different scale bars, showing 2D crumpled or scrolled sheet images. Samples were prepared by dropping DMSO solution of polymer **5** (1 mg/mL) directly unto the copper grid, followed by frozen dry under vacuum.

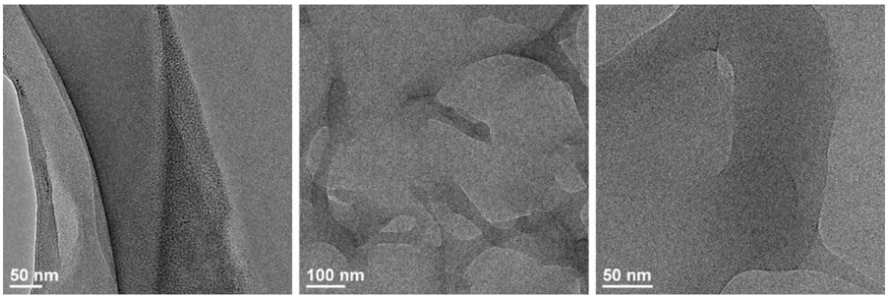

Figure S19. TEM images of supramolecular polymer **5** with different scale bars, showing multilayer structure with crossover and overlapping areas. Samples were prepared by dropping DMSO solution of polymer **5** (1 mg/mL) directly unto the copper grid, followed by frozen dry under vacuum.

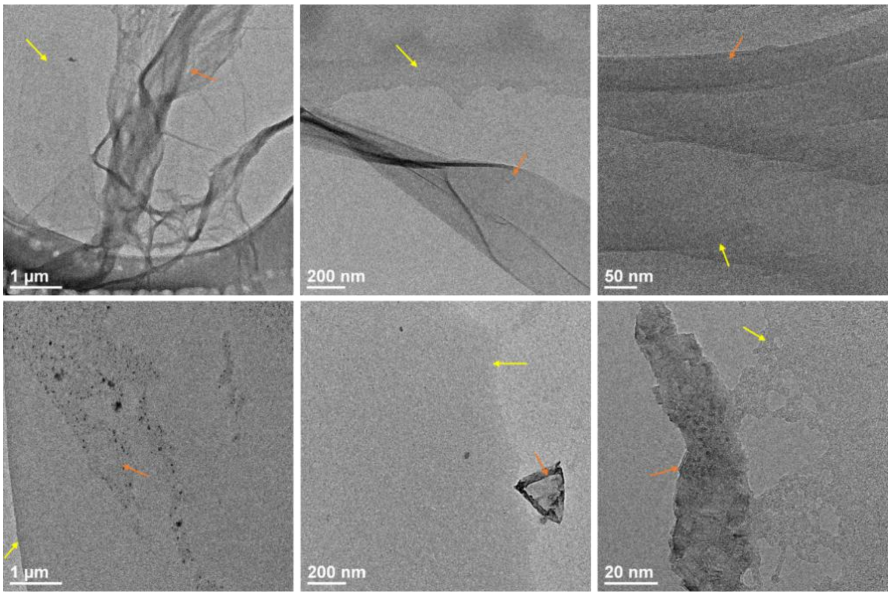

Figure S20. TEM images of supramolecular polymer **5** with different scale bars, showing coexistence of film-like structure with different transparency. Samples were prepared by dropping DMSO solution of polymer **5** (1 mg/mL) directly unto the copper grid, followed by frozen dry under vacuum.

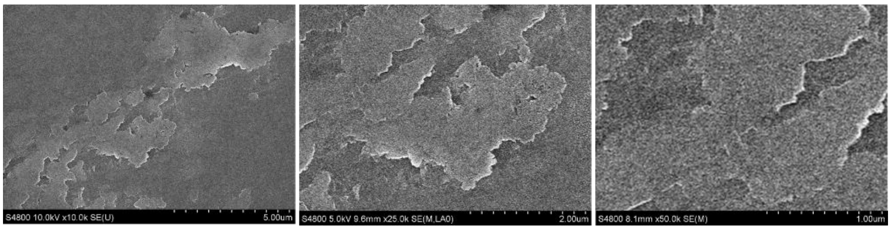

Figure S21. SEM images of supramolecular polymer **5**. Samples were prepared by spinning the DMSO solution of polymer **5** (1 mg/mL) on a silicon substrate, followed by frozen dry under vacuum.

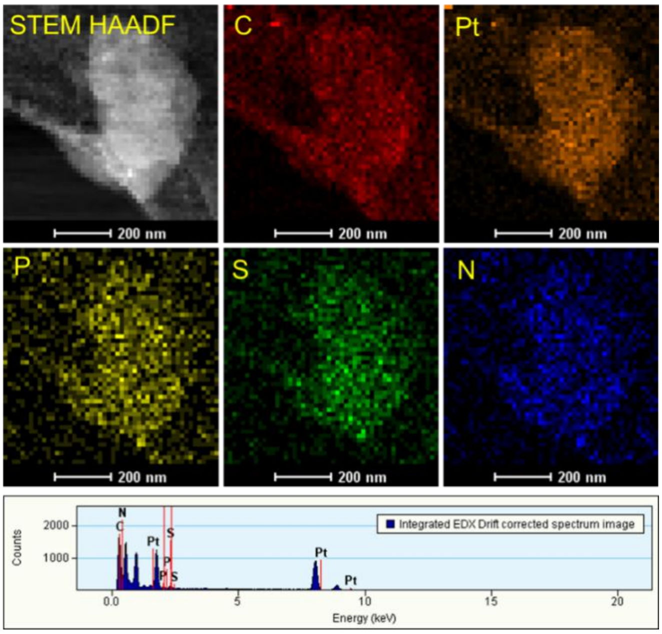

Figure S22. HAADF-STEM image and corresponding elemental mappings, as well as EDS spectrum of supramolecular polymer **5**. Samples were prepared by dropping DMSO solution of polymer **5** (1 mg/mL) directly unto the copper grid, followed by frozen dry under vacuum.

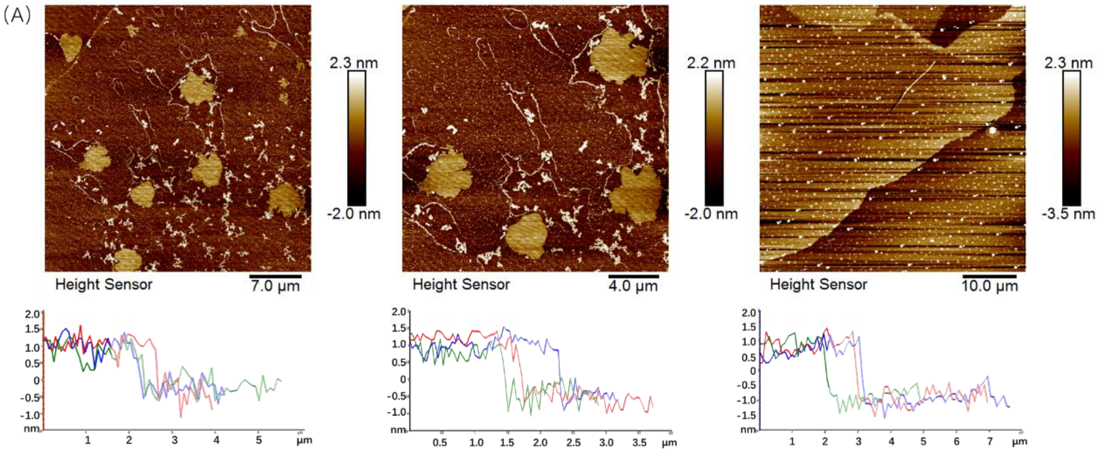

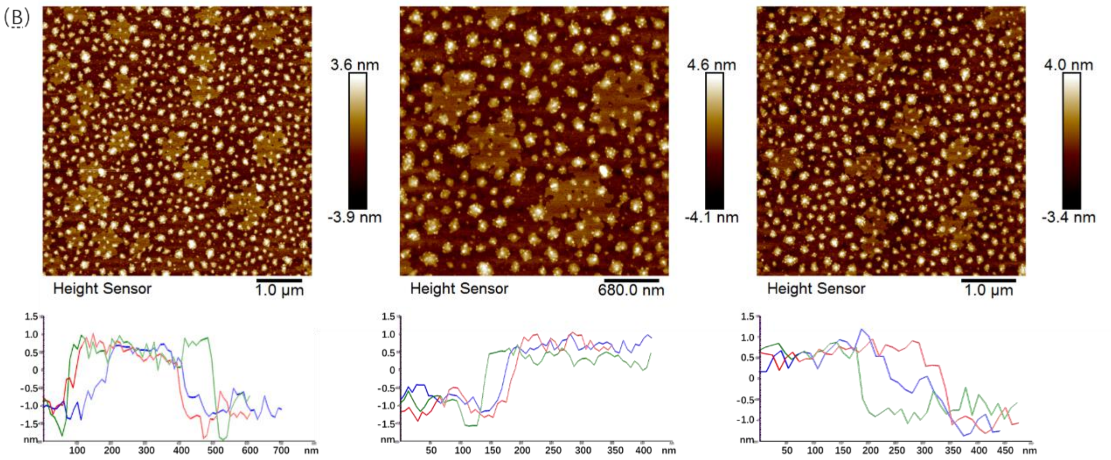

Figure S23. AFM images and height profiles of 2D polymer **5.** (A) Perspective-view of ScanAsyst mode AFM images by using ScanAsyst-Fluid+ probe, and corresponding height profiles of sample prepared by spin coating DMSO solution of polymer **5** (1 mg/mL) onto a mica sheet. (B) Perspective-view of ScanAsyst mode AFM images by using ScanAsyst-Fluid+ probe, and corresponding height profiles of sample prepared by spin coating DMF solution of polymer 5 (1 mg/mL, filtrate twice by using a syringe filter (0.25 μm/33 mm)) onto a mica sheet. The average heights of different regions is about 1.7 nm. Considering the presence of counter anion $SO_3CF_3^-$, the height of the layer regions closely match the calculated monolayer values.

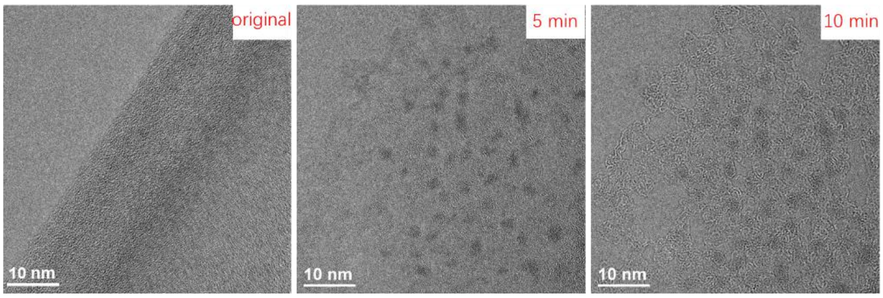

Figure S24. HRTEM images of supramolecular polymer **5** thin film after beam exposure for 10 minutes, showing the ultrathin thickness renders the film sensitive to the electron beam and some holes appeared as the exposure time increased, similar to prior reports on ultrathin nanosheets. Samples were prepared by dropping DMSO solution of polymer **5** (1 mg/mL) directly unto the copper grid, followed by frozen dry under vacuum.

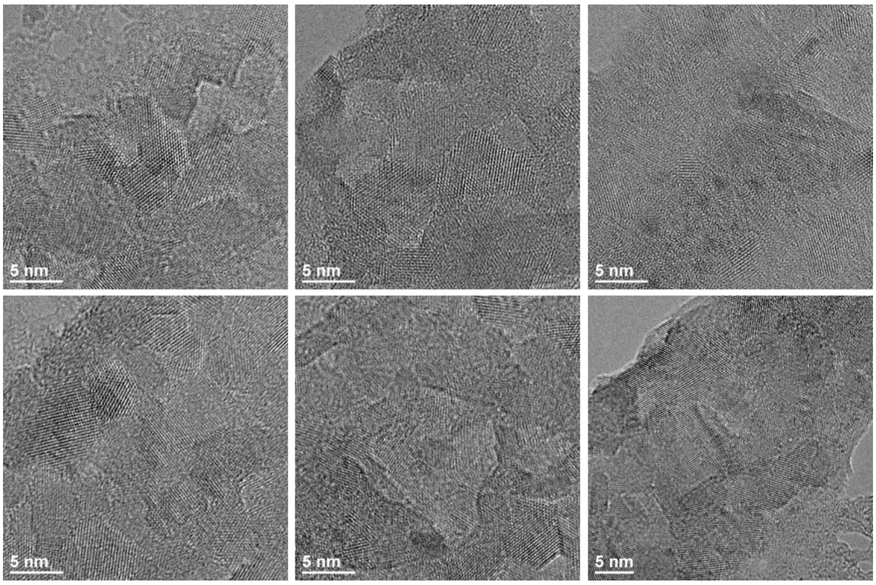

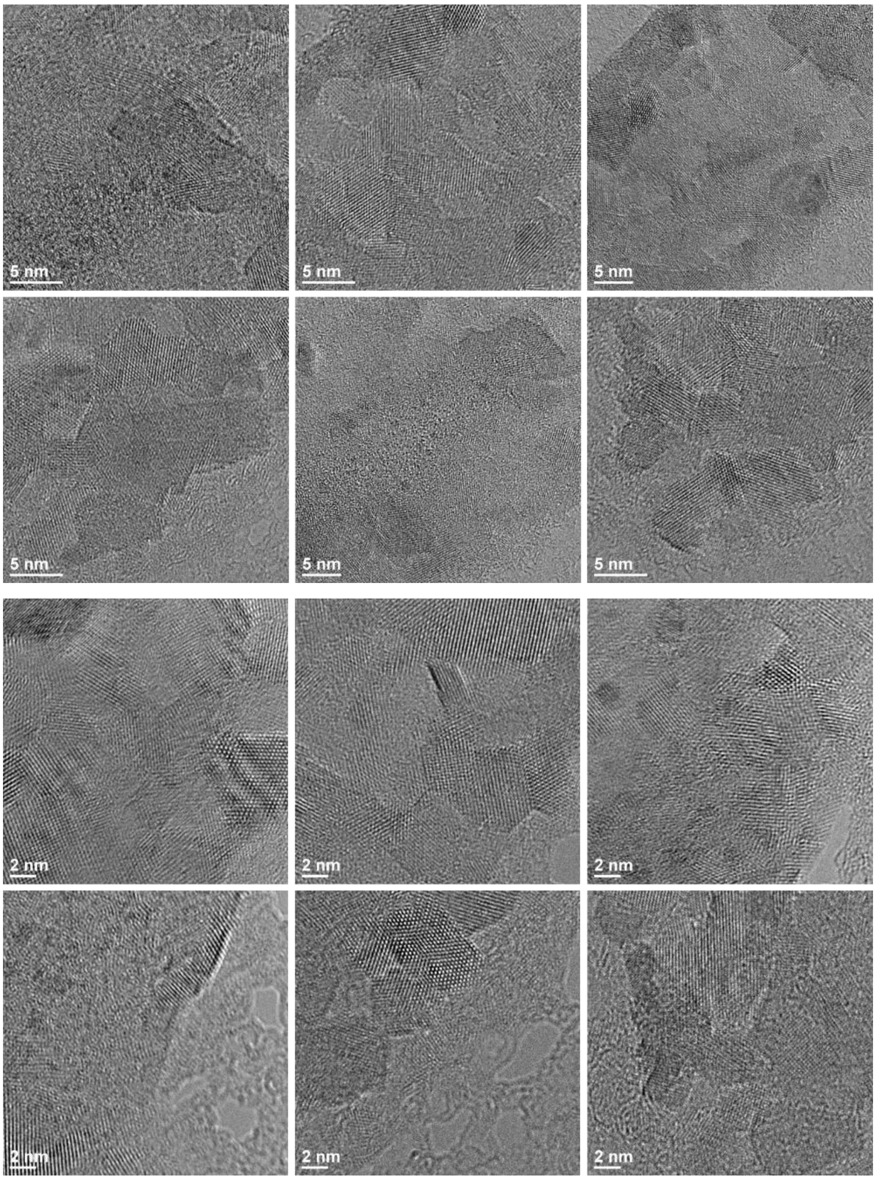

Figure S25. HRTEM images of supramolecular polymer **5** multilayer films from different areas with surface corrugations. Fine lattice structures can be seen in all images. Samples were prepared by dropping DMSO solution of polymer **5** (1 mg/mL) directly unto the copper grid, followed by frozen dry under vacuum.

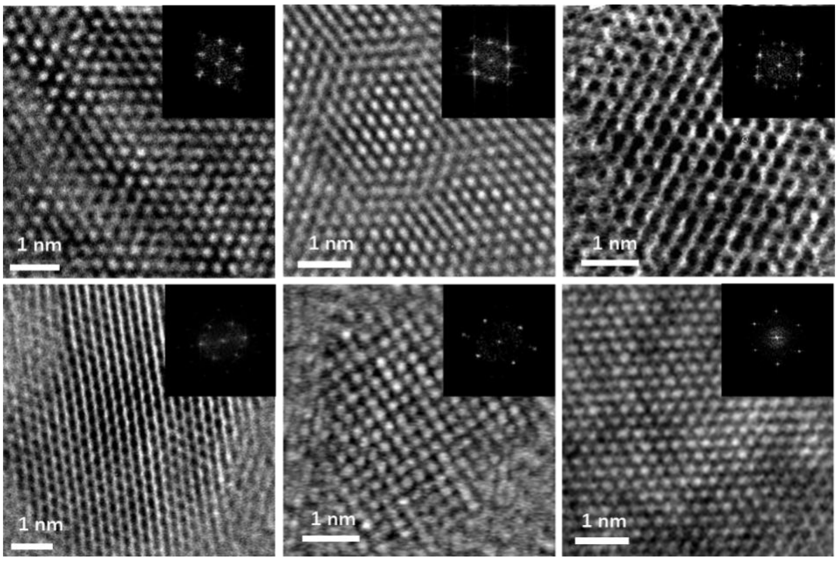

Figure S26. HRTEM images and (inset) the corresponding FFT patterns of supramolecular polymer **5** films from different areas showing crystal corrugations in films. Samples were prepared by dropping DMSO solution of polymer **5** (1 mg/mL) directly unto the copper grid, followed by frozen dry under vacuum.

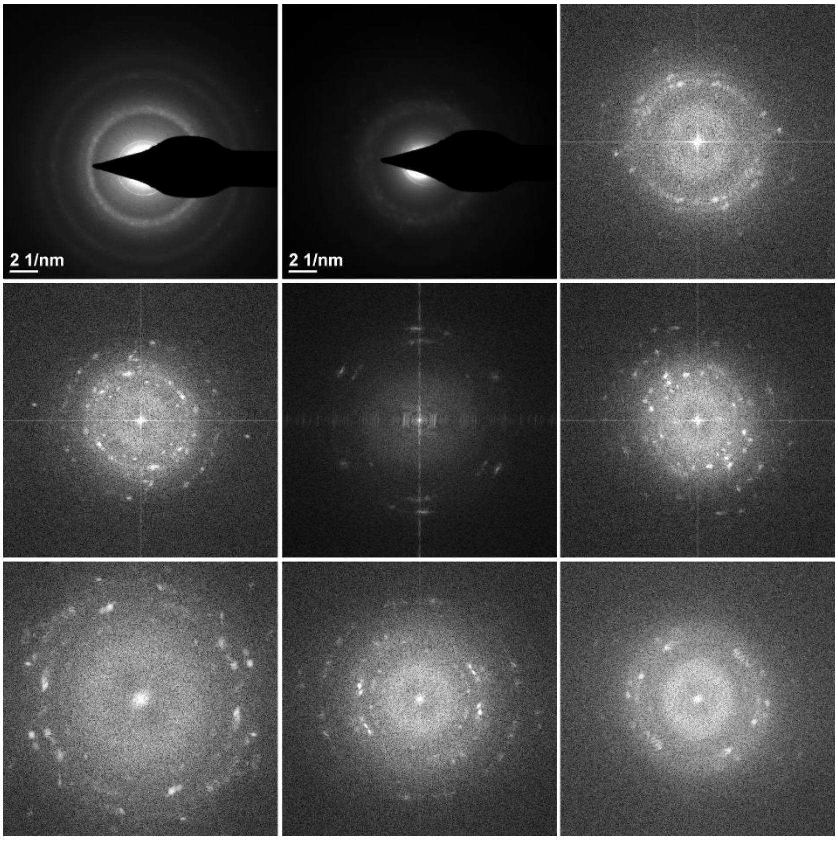

Figure S27. SAED patterns of polymer **5** on different regions, showing polycrystalline with clear boundaries. Samples were prepared by dropping DMSO solution of polymer **5** (1 mg/mL) directly unto the copper grid, followed by frozen dry under vacuum.

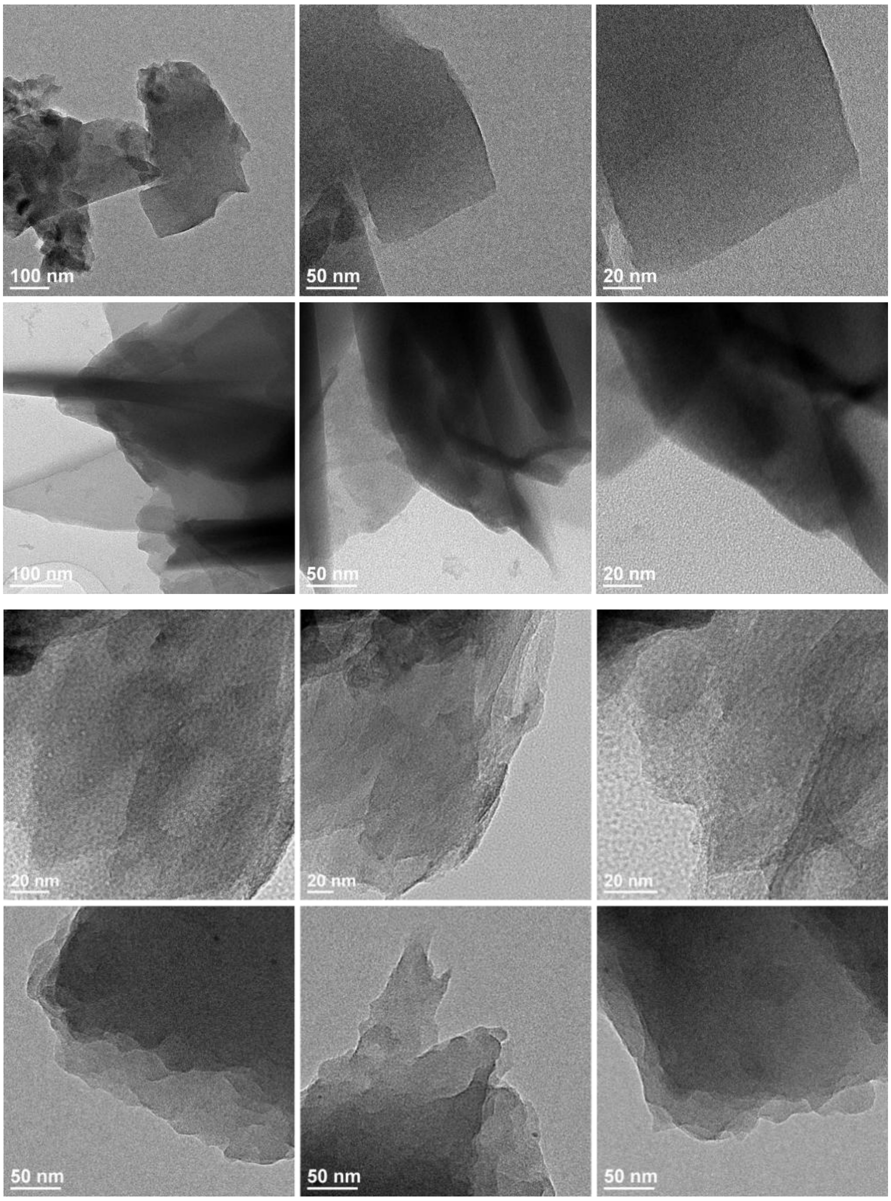

Figure S28. TEM images of supramolecular polymer **5** with different scale bars, showing unrolled and stacked polymer films with different transparency. Samples were prepared by dropping DCM suspensions of polymer **5** directly unto the copper grid.

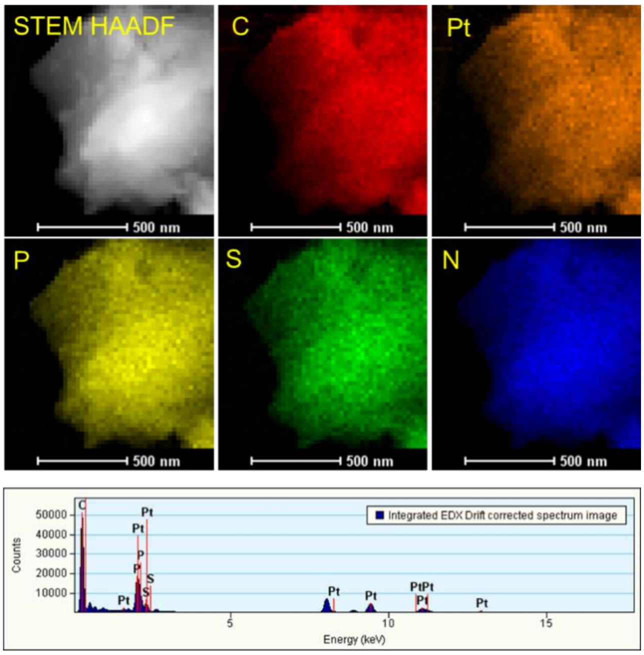

Figure S29. HAADF-STEM image and corresponding elemental mappings, as well as EDS spectrum of supramolecular polymer **5**. Samples were prepared by dropping dichloromethane suspensions of polymer **5** directly unto the copper grid.

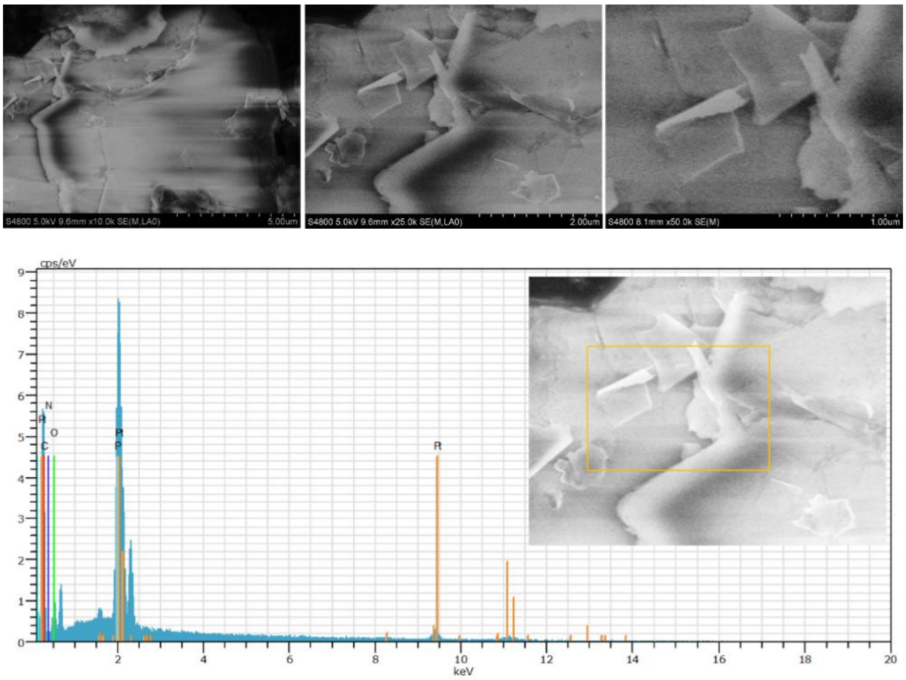

Figure S30. SEM images and related EDX results collected from the area marked by the yellow boxes of supramolecular polymer **5** solid. Samples were prepared by dropping dichloromethane suspensions of polymer **5** on a silicon substrate.

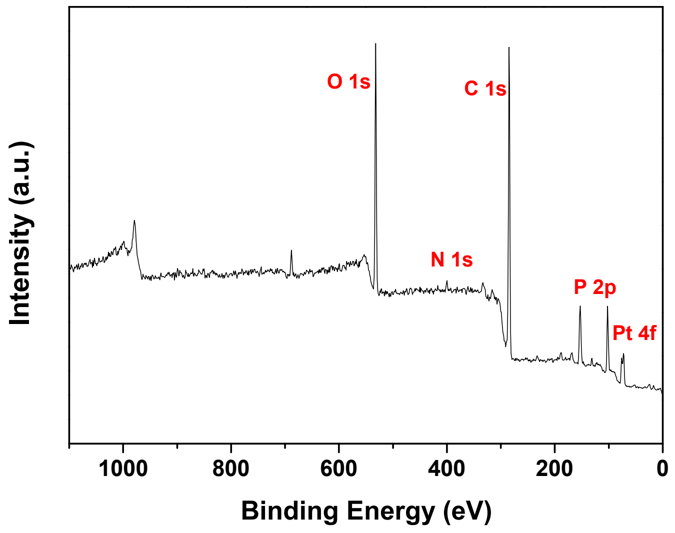

Figure S31. XPS spectrum of supramolecular polymer **5**.

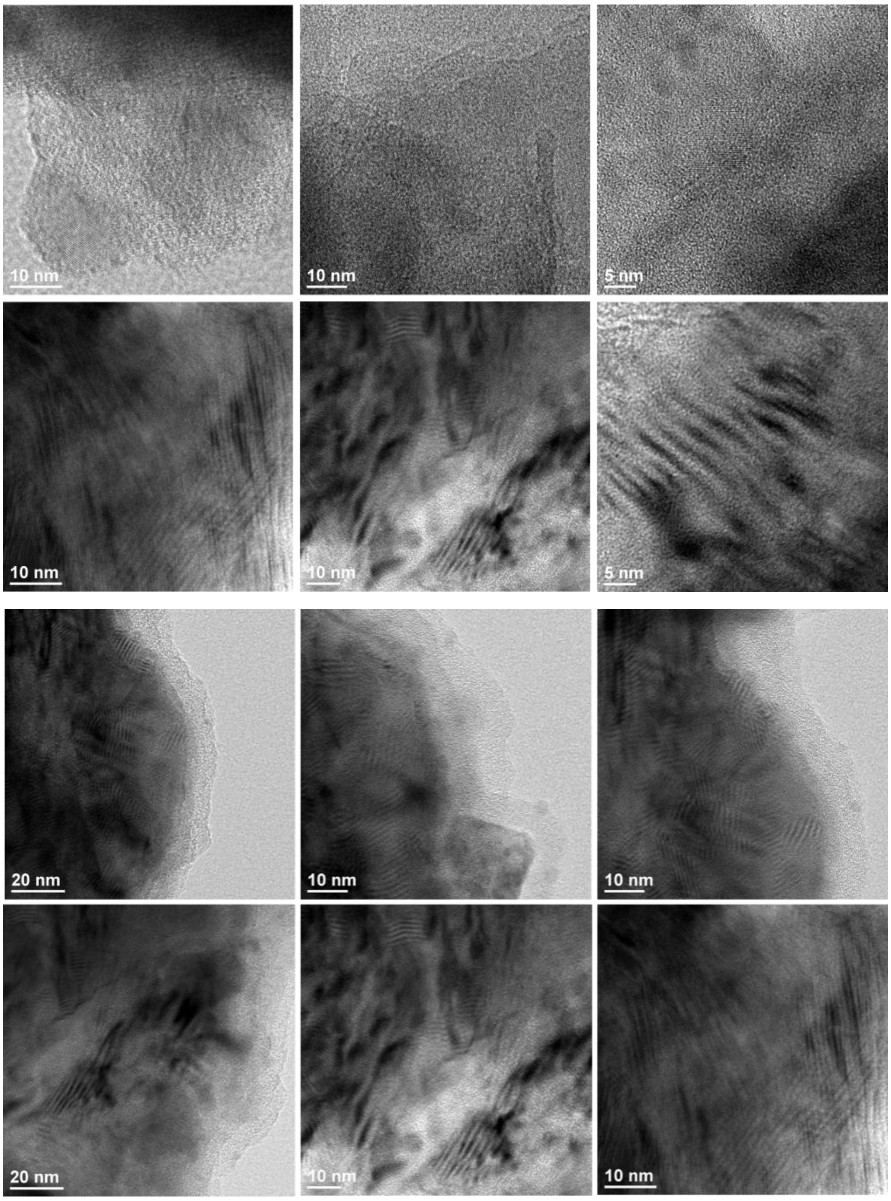

Figure S32. HRTEM images of supramolecular polymer **5** multilayer films from different areas, showing moiré fringes. Fine lattice structures can be seen in all images. Samples were prepared by dropping dichloromethane suspensions of polymer **5** directly unto the copper grid.

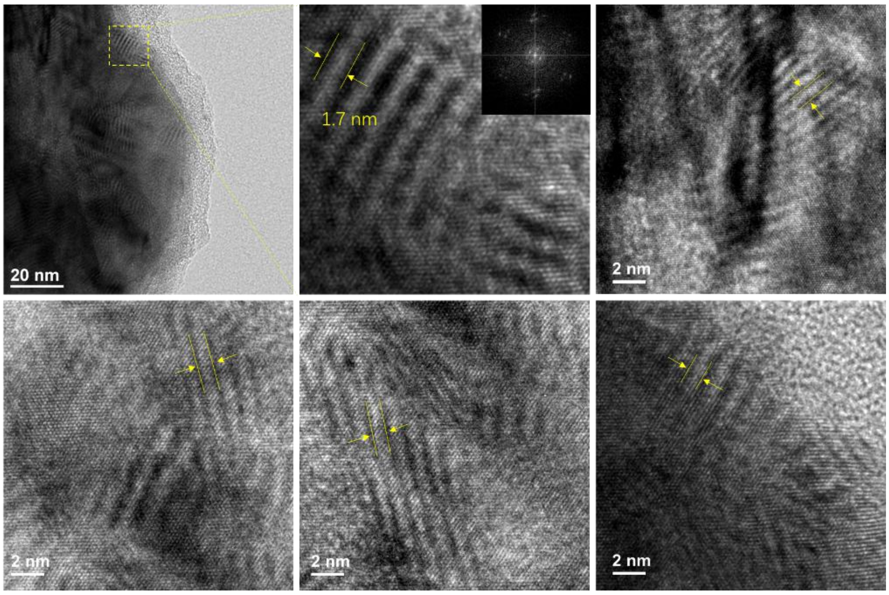

Figure S33. HRTEM images of supramolecular polymer **5**, showing periodic linear moiré patterns with a periodic distance of 1.7 nm, in agreement with periodic distance along one direction of a layered stacking.

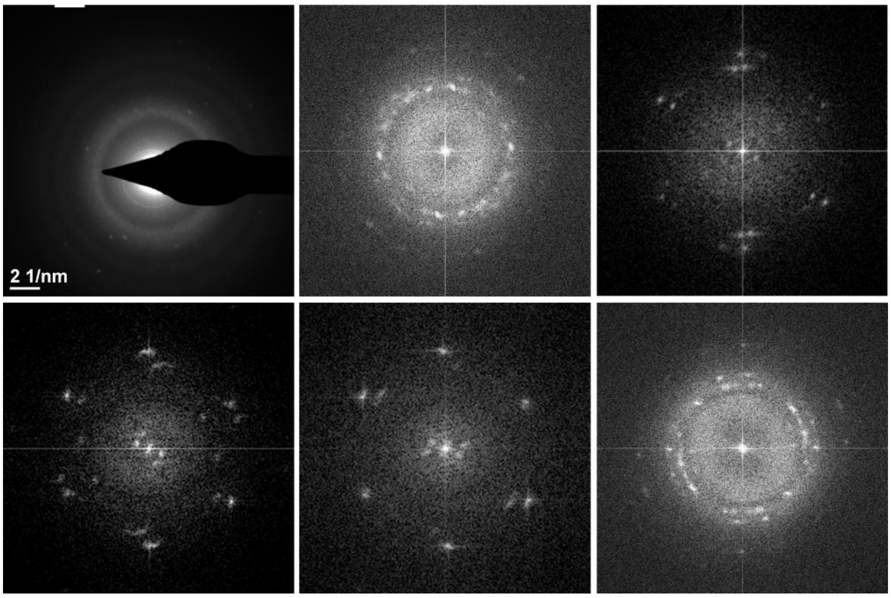

Figure S34. SAED pattern of the polymer **5**, showing a partially crystalline and overall amorphous structure. Samples were prepared by dropping dichloromethane suspensions of polymer **5** directly unto the copper grid.

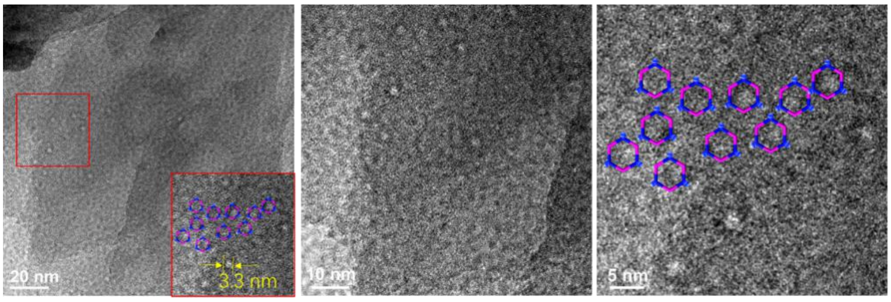

Figure S35. TEM images of supramolecular polymer **5** with different scale bars, showing terraces appearing at the borders, suggesting a multilayer structure, and small pores with a diameter about 3.3 nm at surface, characteristic of the metallacycle pores. Samples were prepared by dropping dichloromethane suspensions of polymer **5** directly unto the copper grid.

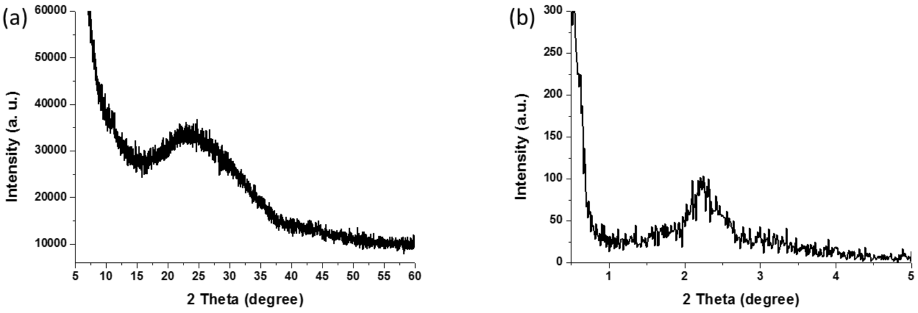

Figure S36. WXRD (a) and SXRD (b) patterns of supramolecular polymer **5**.

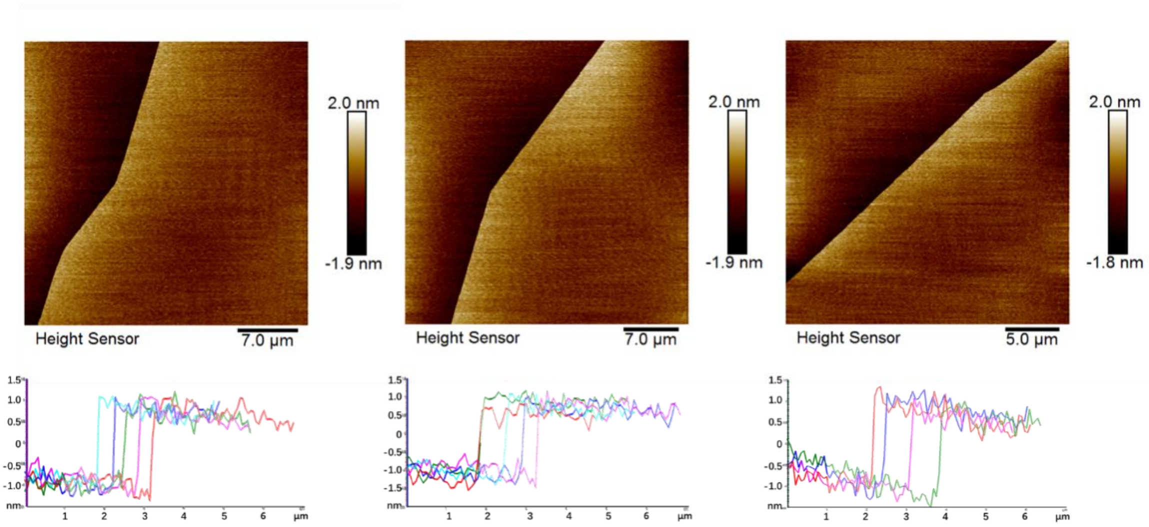

Figure S37. AFM images and height profiles of 2D polymer **5.** Perspective-view of ScanAsyst mode AFM images by using ScanAsyst-Fluid+ probe, and corresponding height profiles of sample prepared by drop coating suspension of polymer **5** obtained after a prolong physical exfoliation by sonication in CH_2Cl_2 onto a mica sheet. The average heights of different regions is about 1.7 nm. Considering the presence of counter anion $SO_3CF_3^-$, the height of the layer regions closely match the calculated monolayer values.

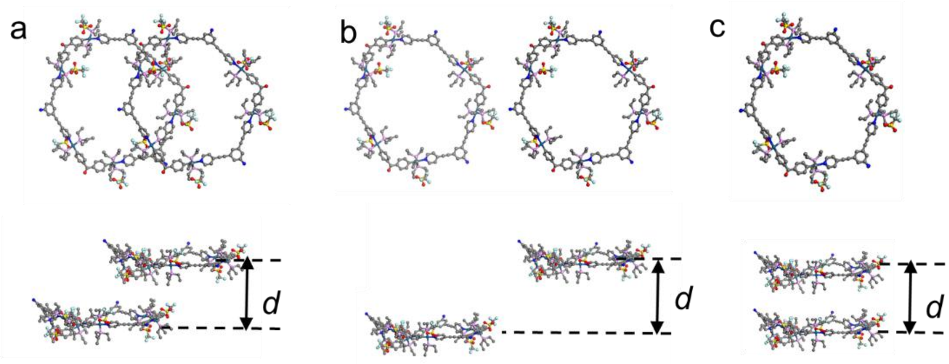

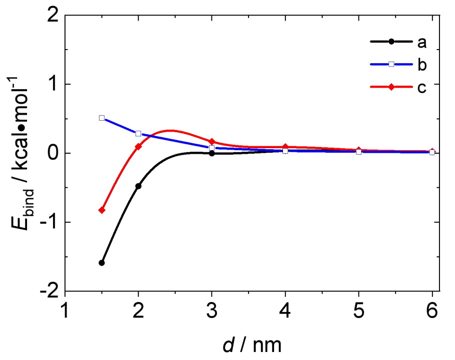

Figure S38. Binding energies calculated at PM7 level in models a, b, and c, where d is the distance between two polymer planes.

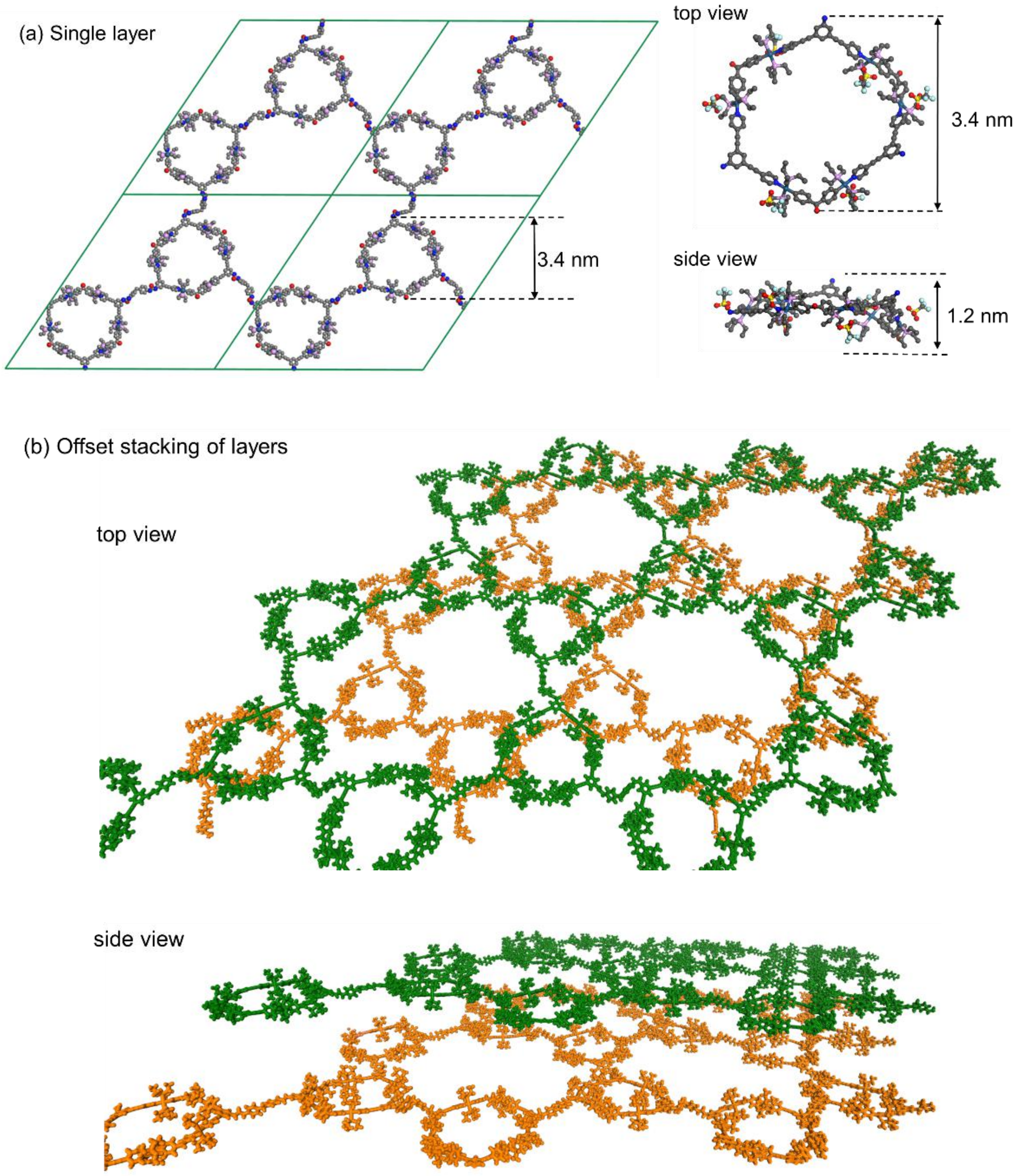

Figure S39. Pictorial representation of single-layer films (a) and offset stacked layers (b) of polymer **5** in which each metallacycle having positive charges on both ends. The dangling arms are omitted for clarity. Metallacycle units are shown in a stick mode with counter ions omitted for clarity.

2.3 Stimuli-responsive disassembly and reassembly of 2D supramolecular polymer 5

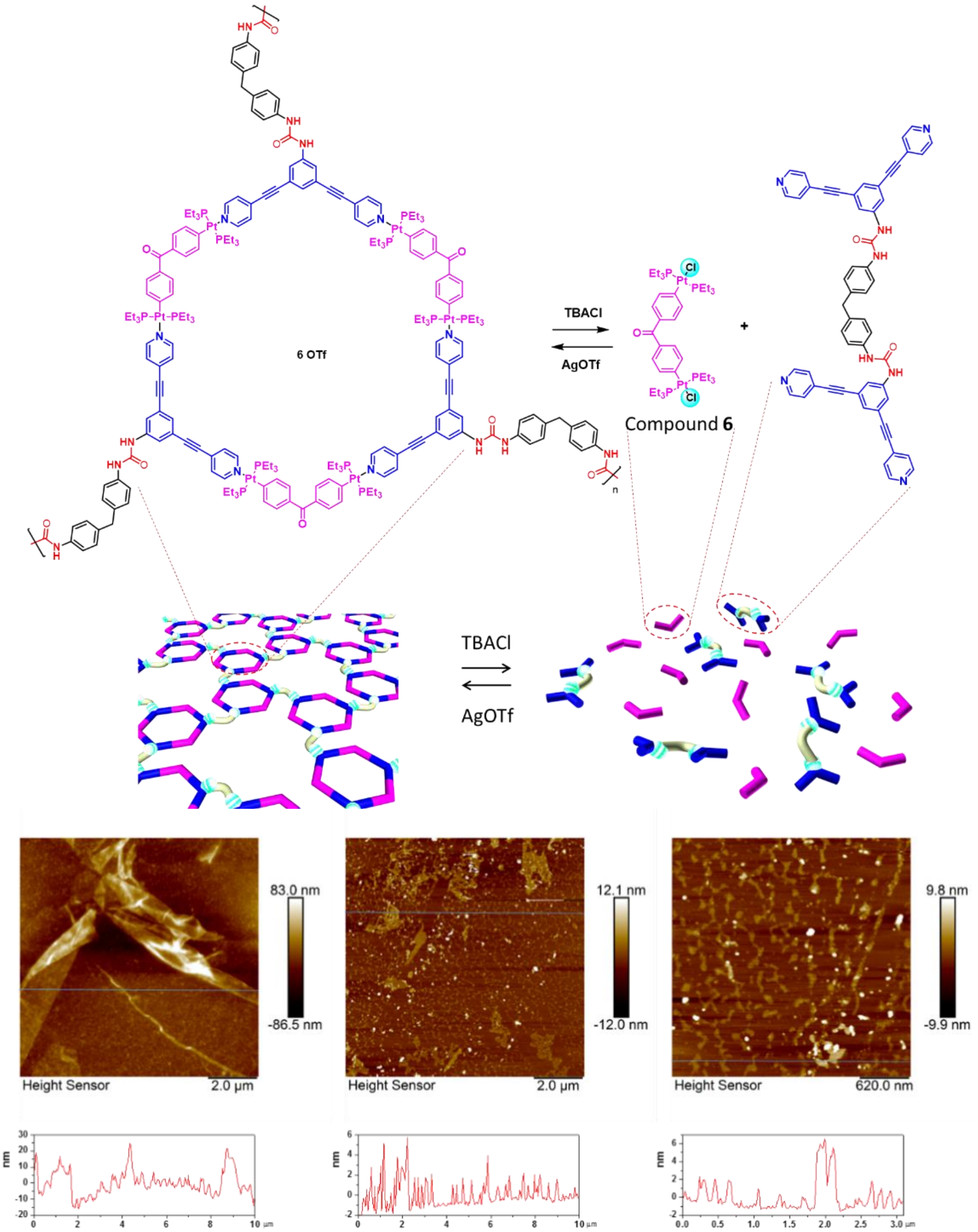

Figure S40. Schematic representation of stimuli-responsive disassembly and reassembly of 2D supramolecular polymer **5** and AFM images as well as the corresponding section profiles along the blue lines of supramolecular polymer **5** (left) and after addition of Bu_4NCl (middle and right).

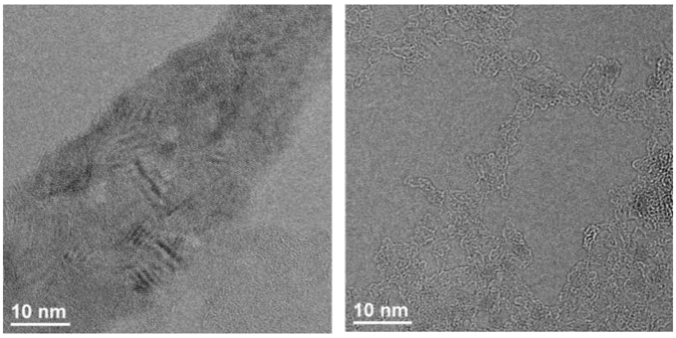

Figure S41. TEM images of supramolecular polymer **5** (left) and after addition of Bu_4NCl (right).

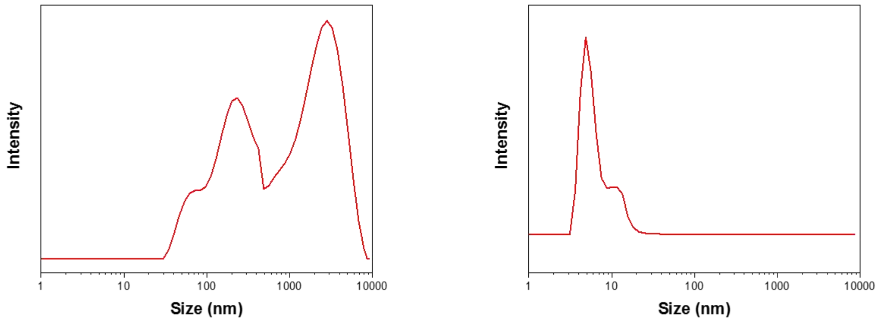

Figure S42. DLS result of size distribution of polymer **5** before (right) and after (right) the addition of Bu_4NCl.

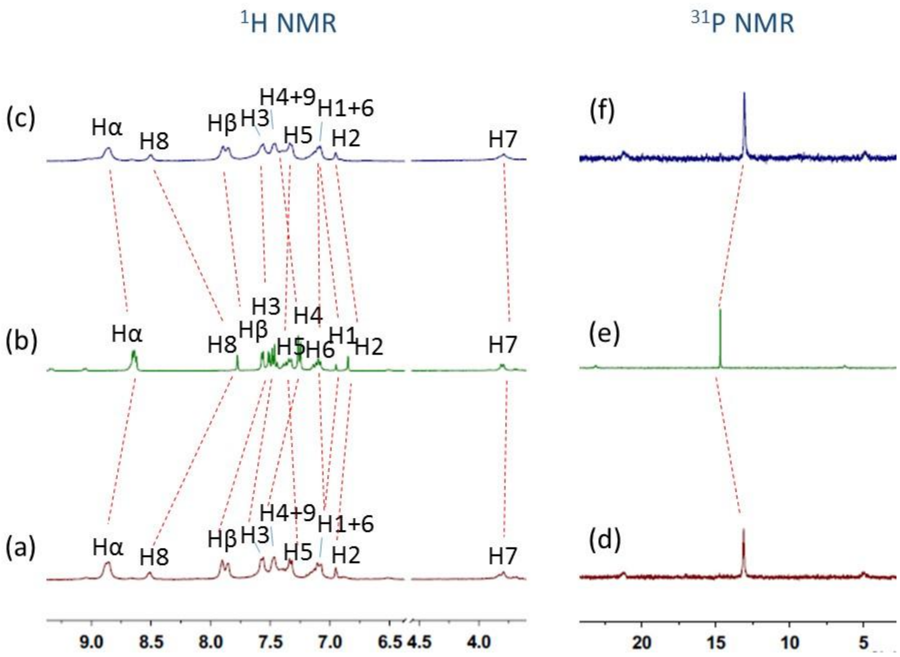

Figure S43. ^{1}H and ^{31}P NMR spectra showing the disassembly and reassembly of supramolecular polymer **5** (400 MHz, 295 K, DMSO-d_6): (a) and (d) supramolecular polymer **5**, (b) and (e) after the excess TBACl, (c) and (f) after subsequent addition AgOTf to the mixture.

2.4 Binding and Affinity Properties of Polymer 5 towards anions and PPs.

*2.4.1 Binding investigation of 2D supramolecular polymer **5** towards various anions.*

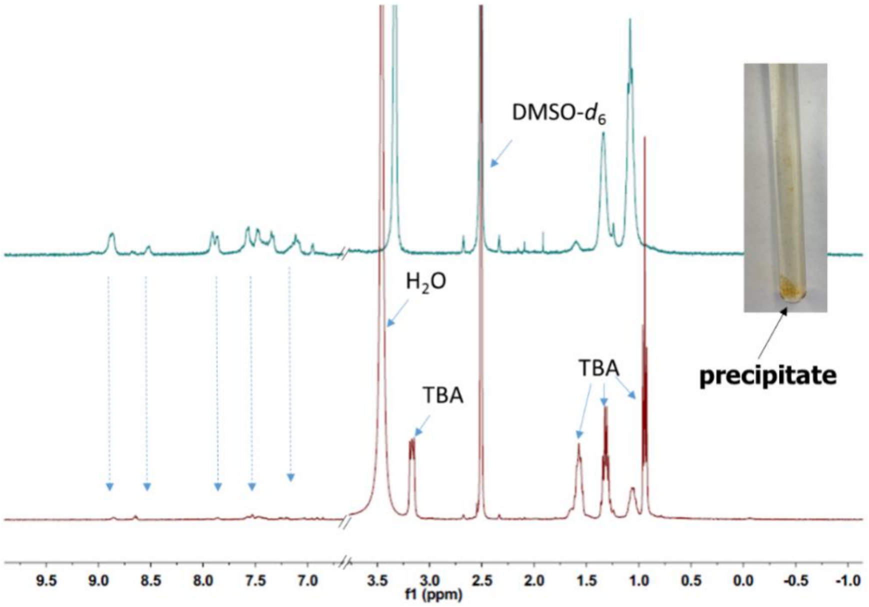

Figure S44. ^{1}H NMR spectra of supramolecular polymer **5** before (top) and after (bottom) interaction with different molar ratios of $H_2PO_4^-$ (Bu_4N^+ (TBA) as cation) in d_6-DMSO at 20 °C. Concentrations (based on the dipyridine units): 1.0 × 10^{-3} mol·L^{-1}. The molar ratios are 1:1. Precipitate were observed for the complexation between polymer **5** and hydrogen phosphate, as indicated in inset image.

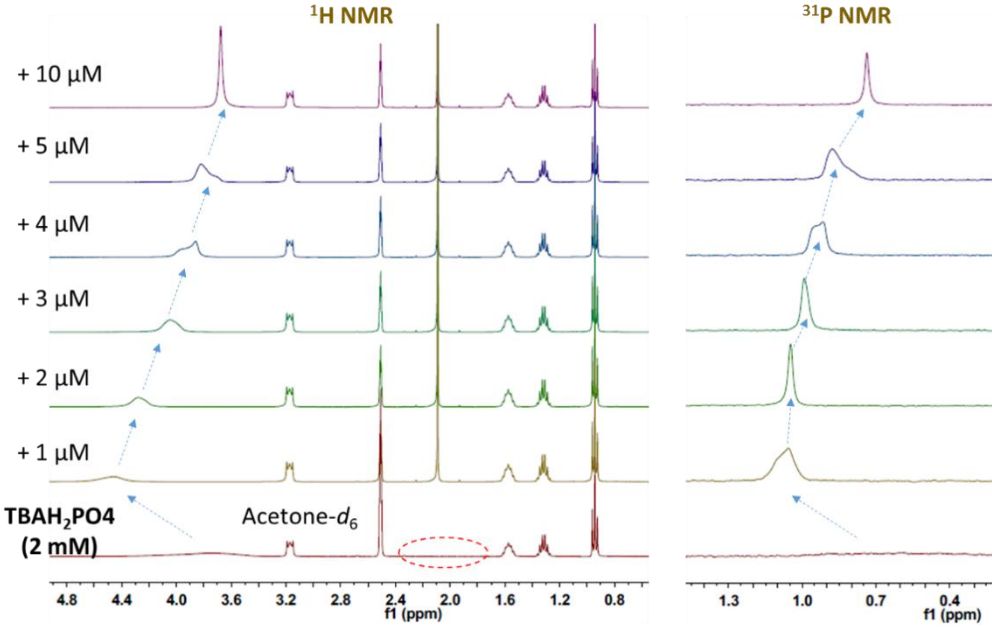

Figure S45. ^{1}H NMR spectra and ^{31}P NMR of $Bu_4NH_2PO_4$ (2 mM) upon the addition of polymer **5** (concentration was calculated based on the dipyridine units) in d_6-acetone at 20 °C.

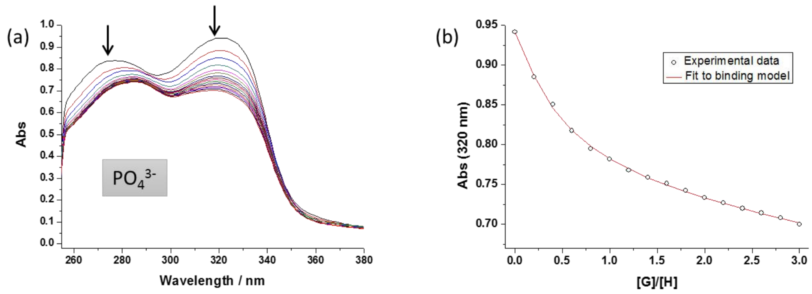

Figure S46. UV-vis titration of polymer **5** (1.0×10^{-5} mol·L^{-1}, concentration was calculated based on dipyridine unit) with PO_4^{3-} in DMSO solution at 20 °C. Tetrabutylammonium (Bu_4N^+) is the counter cation. The absorbance values from 310-330 nm were fitted globally to a 1:1 binding model assuming one dipyridine unit of polymer **5** as one host, giving an apparent binding constant of 4.4×10^5 M^{-1}.

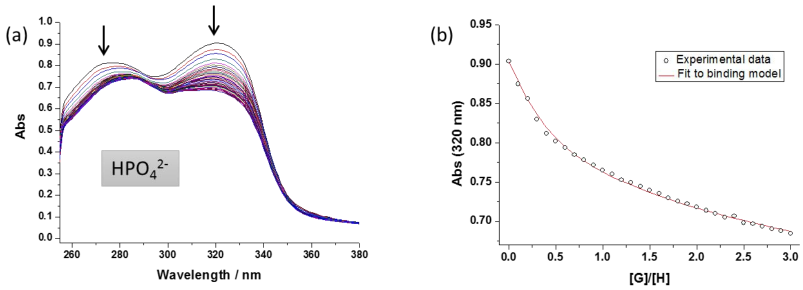

Figure S47. UV-vis titration of polymer **5** (1.0×10^{-5} mol·L^{-1}, concentration was calculated based on dipyridine unit) with HPO_4^{2-} in DMSO solution at 20 °C. Tetrabutylammonium (Bu_4N^+) is the counter cation. The absorbance values from 310-330 nm were fitted globally to a 1:1 binding model assuming one dipyridine unit of polymer **5** as one host, giving an apparent binding constant of 3.3×10^5 M^{-1}.

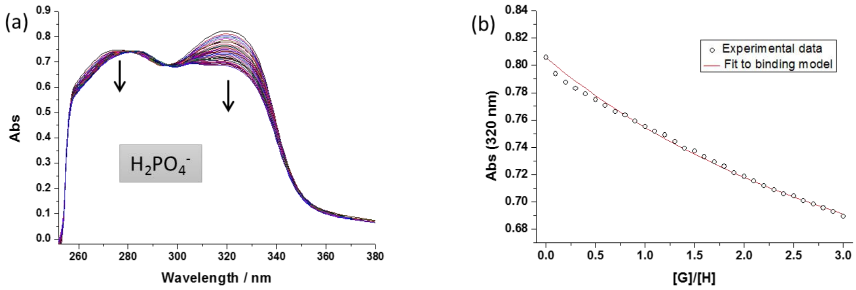

Figure S48. UV-vis titration of polymer **5** (1.0×10^{-5} mol·L^{-1}, concentration was calculated based on dipyridine unit) with $H_2PO_4^-$ in DMSO solution at 20 °C. Tetrabutylammonium (Bu_4N^+) is the counter cation. The absorbance values from 310-330 nm were fitted globally to a 1:1 binding model assuming one dipyridine unit of polymer **5** as one host, giving an apparent binding constant of 3.0×10^5 M^{-1}.

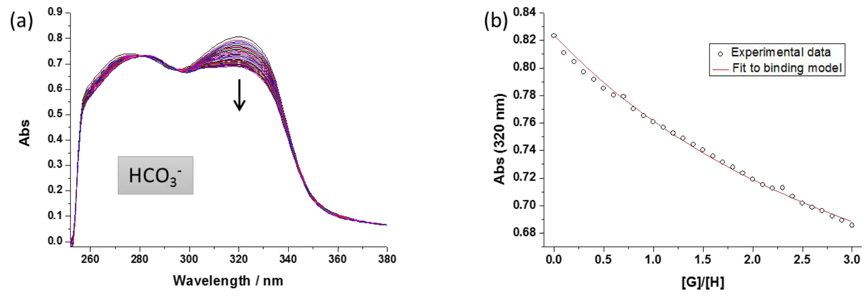

Figure S49. UV-vis titration of polymer **5** (1.0×10^{-5} mol·L^{-1}, concentration was calculated based on dipyridine unit) with HCO_3^- in DMSO solution at 20 °C. Tetrabutylammonium (Bu_4N^+) is the counter cation. The absorbance values from 310-330 nm were fitted globally to a 1:1 binding model assuming one dipyridine unit of polymer **5** as one host, giving an apparent binding constant of 3.5×10^4 M^{-1}.

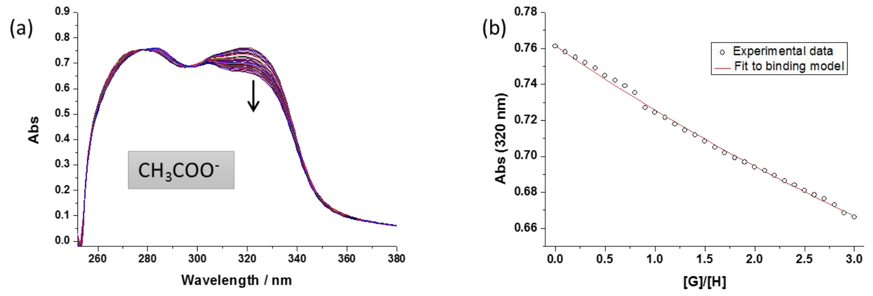

Figure S50. UV-vis titration of polymer **5** (1.0×10^{-5} mol·L^{-1}, concentration was calculated based on dipyridine unit) with CH_3COO^- in DMSO solution at 20 °C. Tetrabutylammonium (Bu_4N^+) is the counter cation. The absorbance values from 310-330 nm were fitted globally to a 1:1 binding model assuming one dipyridine unit of polymer **5** as one host, giving an apparent binding constant of 8.1×10^3 M^{-1}.

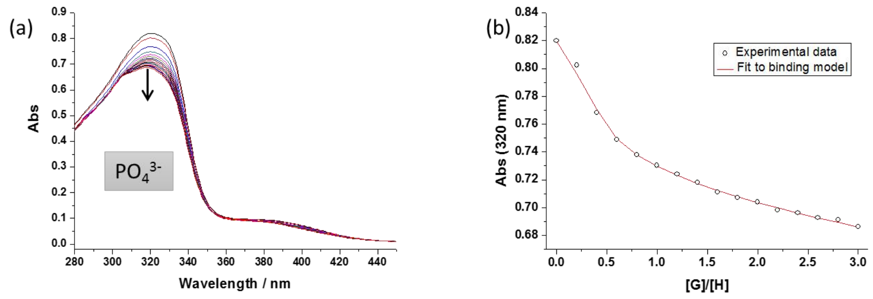

Figure S51. UV-vis titration of metallacycle **3** (1.0×10^{-5} mol·L^{-1}, concentration was calculated based on dipyridine unit) with PO_4^{3-} in DMSO solution at 20 °C. Tetrabutylammonium (Bu_4N^+) is the counter cation. The absorbance values from 310-330 nm were fitted globally to a 1:1 binding model assuming one dipyridine unit of metallacycle **3** as one host, giving an apparent binding constant of 4.6×10^5 M^{-1}.

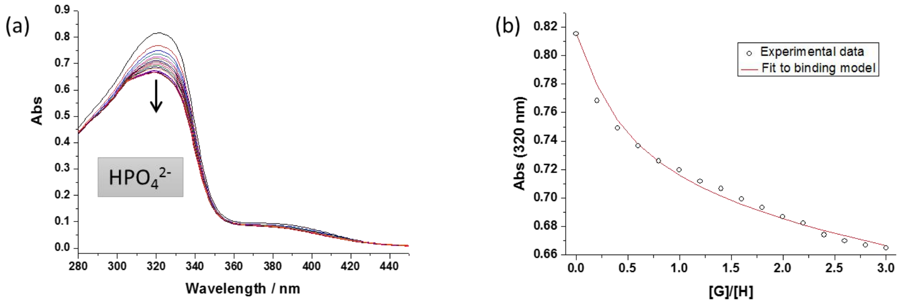

Figure S52. UV-vis titration of metallacycle **3** (1.0×10^{-5} mol·L^{-1}, concentration was calculated based on dipyridine unit) with HPO_4^{2-} in DMSO solution at 20 °C. The absorbance values from 310-330 nm were fitted globally to a 1:1 binding model assuming one dipyridine unit of metallacycle **3** as one host, giving an apparent binding constant of 3.5×10^5 M^{-1}.

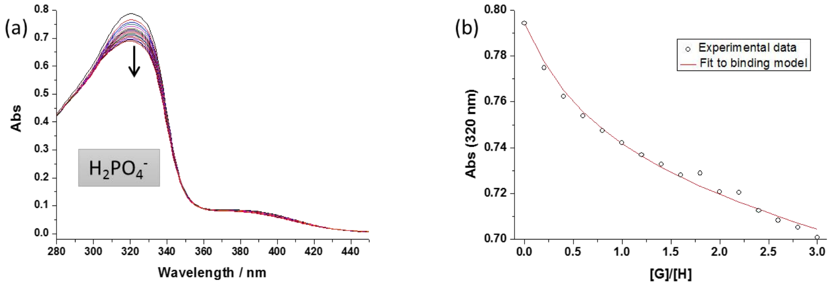

Figure S53. UV-vis titration of metallacycle **3** (1.0×10^{-5} mol·L^{-1}, concentration was calculated based on dipyridine unit) with $H_2PO_4^-$ in DMSO solution at 20 °C. Tetrabutylammonium (Bu_4N^+) is the counter cation. The absorbance values from 310-330 nm were fitted globally to a 1:1 binding model assuming one dipyridine unit of metallacycle **3** as one host, giving an apparent binding constant of 1.5×10^5 M^{-1}.

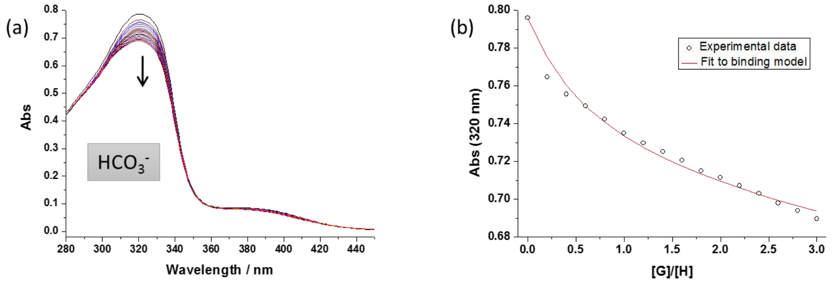

Figure S54. UV-vis titration of metallacycle **3** (1.0×10^{-5} mol·L^{-1}, concentration was calculated based on dipyridine unit) with HCO_3^- in DMSO solution at 20 °C. Tetrabutylammonium (Bu_4N^+) is the counter cation. The absorbance values from 310-330 nm were fitted globally to a 1:1 binding model assuming one dipyridine unit of metallacycle **3** as one host, giving an apparent binding constant of 1.2×10^5 M^{-1}

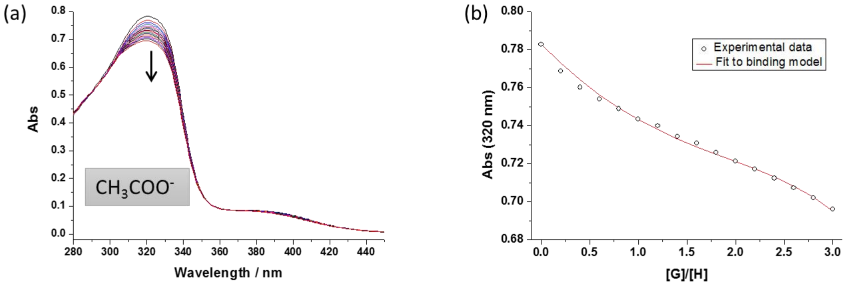

Figure S55. UV-vis titration of metallacycle **3** (1.0×10^{-5} mol·L^{-1}, concentration was calculated based on dipyridine unit) with CH_3COO^- in DMSO solution at 20 °C. Tetrabutylammonium (Bu_4N^+) is the counter cation. The absorbance values from 310-330 nm were fitted globally to a 1:1 binding model assuming one dipyridine unit of metallacycle **3** as one host, giving an apparent binding constant of 3.8×10^4 M^{-1}.

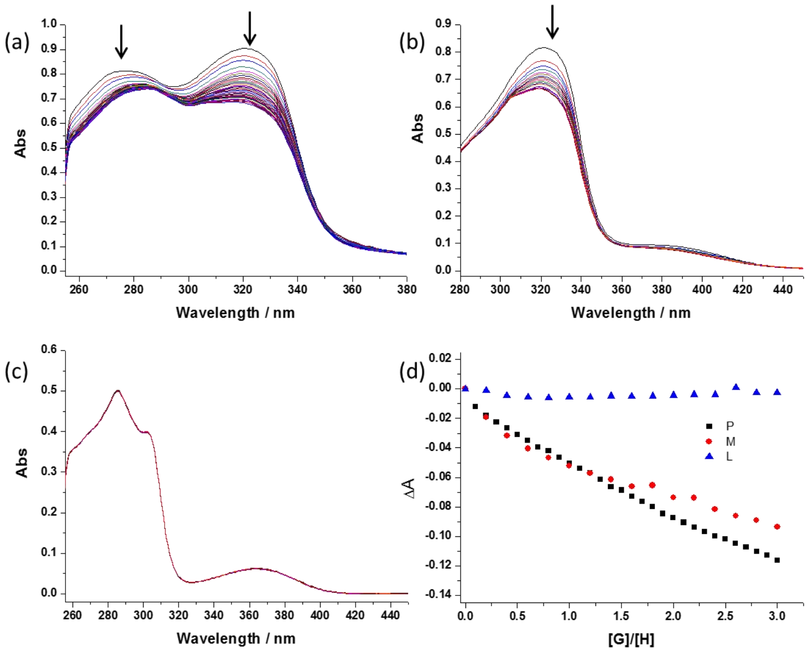

Figure S56. UV spectra of polymer **5** (1.0×10^{-5} mol·L^{-1}, concentration was calculated based on dipyridine unit, a), metallacycle **3** (1.0×10^{-5} mol·L^{-1}, concentration was calculated based on dipyridine unit, b), and dipyridine ligand **1** (1.0×10^{-5} mol·L^{-1}, c) upon addition of different equivalents of $H_2PO_4^-$ in DMSO solution at 20 °C. Tetrabutylammonium (Bu_4N^+) is the counter cation. The UV intensity changes (d) of different host upon the additions of anion guests, [G]/[H] is an abbreviation of the molar ratio of guest to host.

Table S1. Apparent association constants (K_a) of polymer **5** and metallacycle **3** interacted with various anions in dimethylsulfoxide (DMSO) at 20 °C.

Anions	K_a (L·mol^{-1})[a]	
	with polymer **5** [b]	with metallacycle **3**[c]
PO_4^{3-}	(4.4±0.2) ×10^5	(4.6±0.2) ×10^5
HPO_4^{2-}	(3.3±0.1) ×10^5	(3.5±0.3) ×10^5
$H_2PO_4^-$	(3.0±0.1) ×10^5	(1.5±0.1) ×10^5
HCO_3^-	(3.5±0.2) ×10^4	(1.2±0.3) ×10^5
CH_3COO^-	(8.1±0.1) ×10^3	(3.8±0.6) ×10^4

[a] K_a values were determined by fitting to 1:1 binding models using a custom written *python* program *BindFit* developed and deployed on the web www.supramolecular.org. Data are from n = 3 independent experiments and are presented as mean ± s.e.m [b] Values were obtained from UV-Vis titration experiments based on the absorption intensity changes at the maximal absorption peaks (approximately 320 nm). [c] Values were obtained from UV-Vis titration experiments based on the absorption intensity changes at the maximal absorption peaks (approximately 319 nm).

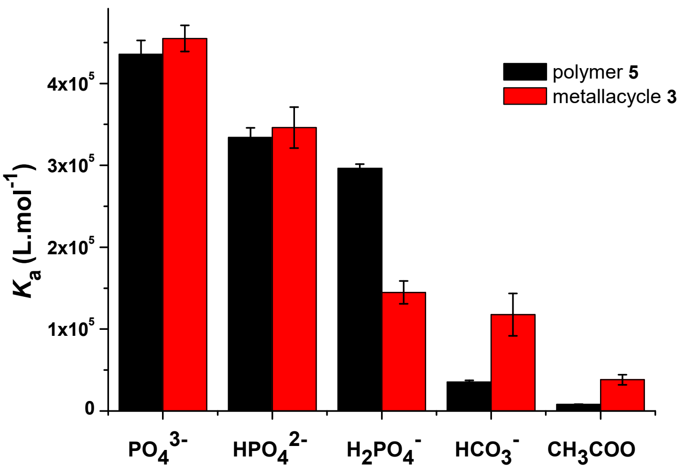

Figure S57. Comparison of apparent association constants K_a of polymer **5** and metallacycle **3** with various anions. Data are from n = 3 independent experiments and are presented as mean ± s.e.m.

*2.4.2 Binding and affinity investigation of 2D supramolecular polymer **5** towards model PPs.*

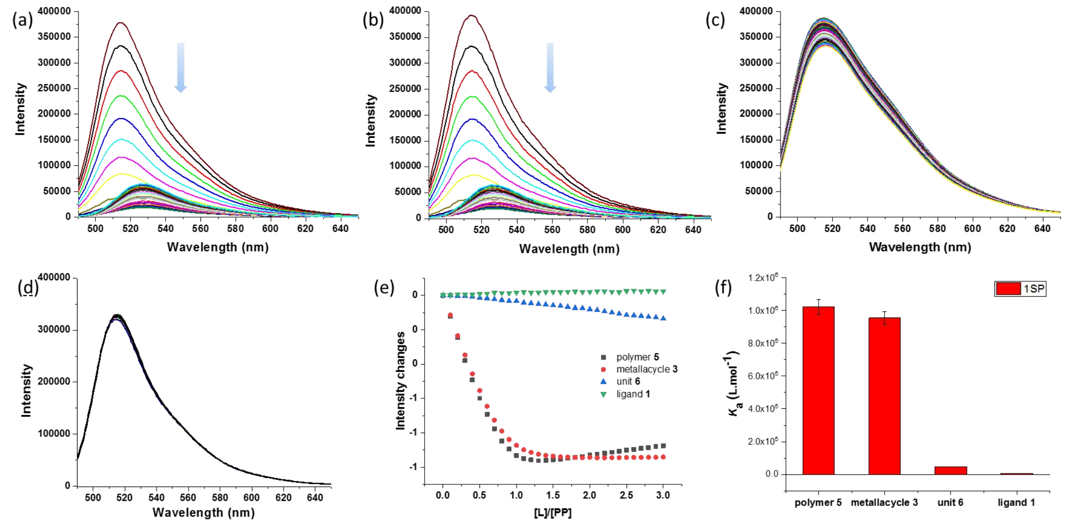

Figure S58. Typical fluorescence spectra of *N*-terminus fluorescein-labelled serinephosphorylated peptide 1SP (1.0 × 10^{-6} mol·L^{-1}) upon addition of different equivalents of polymer **5** (a), metallacycle **3** (b), acceptor unit **6** (c) or ligand **1** (d) guests in aqueous solution at 20 °C, and the fluorescent intensity changes (e) and comparison of apparent K_a (f) of peptide host 1SP upon the additions of guests (concentrations were calculated based on the dipyridine units). [L]/[PP] is an abbreviation of the molar ratio of guest to host.

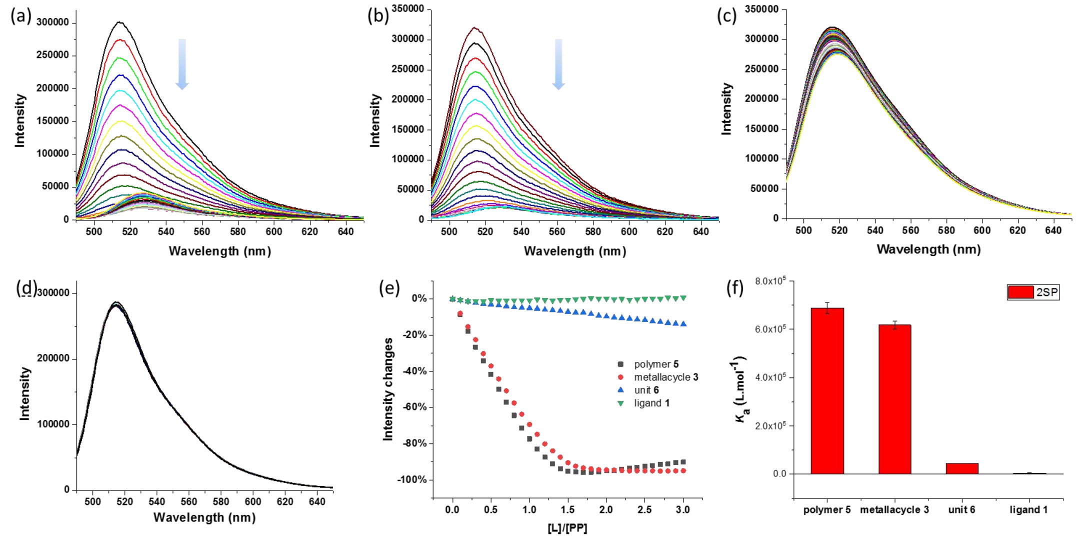

Figure S59. Typical fluorescence spectra of *N*-terminus fluorescein-labelled serinephosphorylated peptide 2SP (1.0 × 10^{-6} mol·L^{-1}) upon addition of different equivalents of polymer **5** (a), metallacycle **3** (b), acceptor unit **6** (c) or ligand **1** (d) guests in aqueous solution at 20 °C, and the fluorescent intensity changes (e) and comparison of apparent K_a (f) of peptide host 2SP upon the additions of guests (concentrations were calculated based on the dipyridine units). [L]/[PP] is an abbreviation of the molar ratio of guest to host.

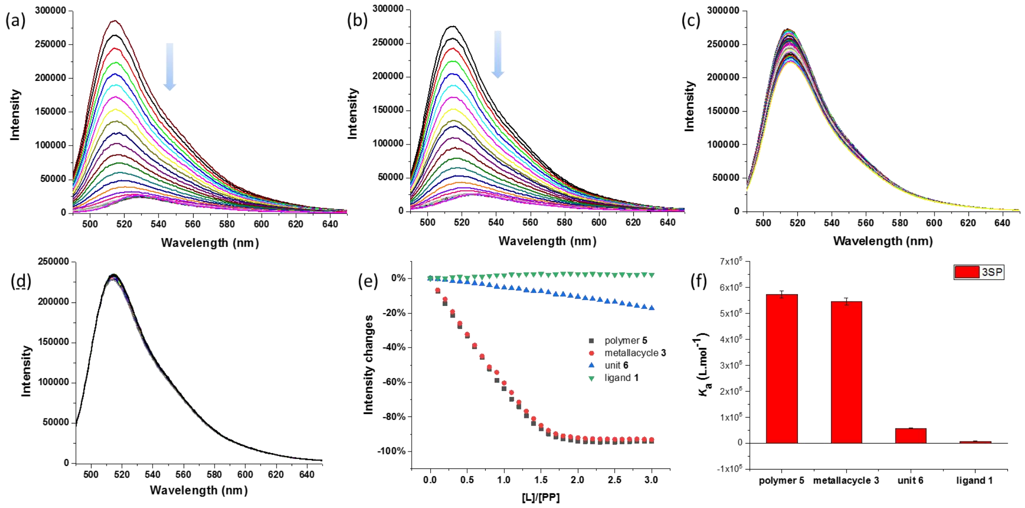

Figure S60. Typical fluorescence spectra of *N*-terminus fluorescein-labelled serinephosphorylated peptide 3SP (1.0 × 10^{-6} mol·L^{-1}) upon addition of different equivalents of polymer **5** (a), metallacycle **3** (b), acceptor unit **6** (c) or ligand **1** (d) guests in aqueous solution at 20 °C, and the fluorescent intensity changes (e) and comparison of apparent K_a (f) of peptide host 3SP upon the additions of guests (concentrations were calculated based on the dipyridine units). [L]/[PP] is an abbreviation of the molar ratio of guest to host.

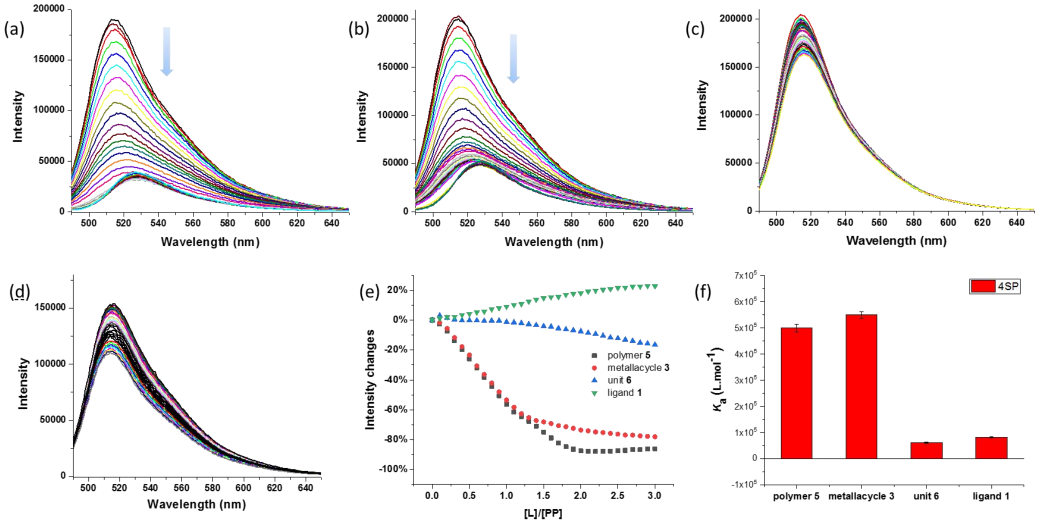

Figure S61. Ttypical fluorescence spectra of *N*-terminus fluorescein-labelled serinephosphorylated peptide 4SP (1.0 × 10^{-6} mol·L^{-1}) upon addition of different equivalents of polymer **5** (a), metallacycle **3** (b), acceptor unit **6** (c) or ligand **1** (d) guests in aqueous solution at 20 °C, and the fluorescent intensity changes (e) and comparison of apparent K_a (f) of peptide host 4SP upon the additions of guests (concentrations were calculated based on the dipyridine units). [L]/[PP] is an abbreviation of the molar ratio of guest to host.

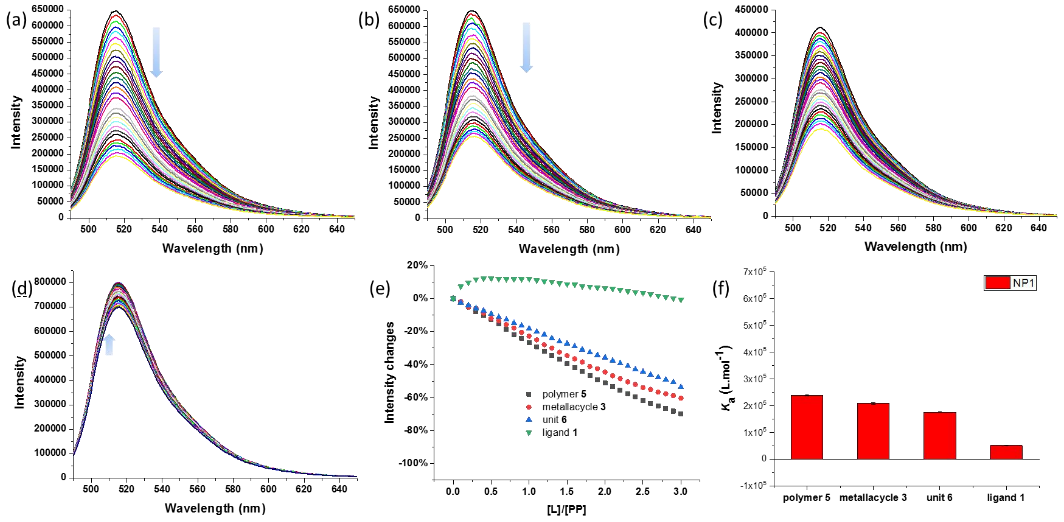

Figure S62. Typical fluorescence spectra of *N*-terminus fluorescein-labelled serinephosphorylated peptide NP1 (1.0 × 10^{-6} mol·L^{-1}) upon addition of different equivalents of polymer **5** (a), metallacycle **3** (b), acceptor unit **6** (c) or ligand **1** (d) guests in aqueous solution at 20 °C, and the fluorescent intensity changes (e) and comparison of apparent K_a (f) of peptide host NP1 upon the additions of guests (concentrations were calculated based on the dipyridine units). [L]/[PP] is an abbreviation of the molar ratio of guest to host.

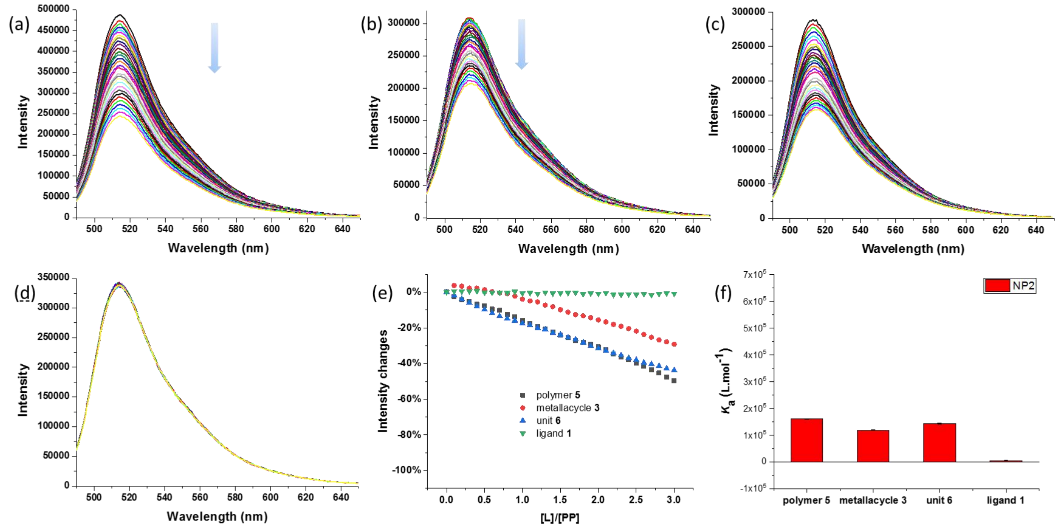

Figure S63. Typical fluorescence spectra of *N*-terminus fluorescein-labelled serinephosphorylated peptide NP2 (1.0 × 10^{-6} mol·L^{-1}) upon addition of different equivalents of polymer **5** (a), metallacycle **3** (b), acceptor unit **6** (c) or ligand **1** (d) guests in aqueous solution at 20 °C, and the fluorescent intensity changes (e) and comparison of apparent K_a (f) of peptide host NP2 upon the additions of guests (concentrations were calculated based on the dipyridine units). [L]/[PP] is an abbreviation of the molar ratio of guest to host.

Table S2. Apparent association constants (K_a) of N-terminus fluorescein-labelled serine mono-, di, tri, tetra-PPs (1SP—4SP) and non-phosphorylated peptide NP1-NP2 interacted with polymer **5**, metallacycle **3**, unit **6** and ligand **1** in aqueous solution at 20 °C.

Peptides	K_a (L·mol^{-1})[a]			
	with polymer **5**	with metallacycle **3**	with unit **6**[b]	with ligand **1**[b]
1SP	(1.1±0.1) ×10^6	(1.1±0.1) ×10^6	(4.7±0.7) ×10^4	(7.5±0.6) ×10^3
2SP	(7.7±0.2) ×10^5	(6.7±0.2) ×10^5	(4.4±0.5) ×10^4	(4.9±0.7) ×10^3
3SP	(6.3±0.1) ×10^5	(6.5±0.1) ×10^5	(5.7±0.7) ×10^4	(7.3±1.0) ×10^3
4SP	(5.8±0.2) ×10^5	(7.2±0.2) ×10^5	(6.1±0.3) ×10^4	(8.2±0.2) ×10^4
NP1	(2.6±0.1) ×10^5	(2.2±0.1) ×10^5	(1.7±0.7) ×10^5	(5.1±0.2) ×10^4
NP2	(1.6±0.1) ×10^5	(1.2±0.1) ×10^5	(1.4±0.2) ×10^5	(5.2±0.6) ×10^3

[a] apparent K_a values were obtained from fluorescent titration experiments based on the fluorescent intensity changes at the maximal emission peaks (approximately 518 nm); All error values were obtained by the results of linear curve fitting according to modified Stern-Volmer quenching equation,

$$(F_{max} - F)/(F_{max} - F_{min}) = K_a[G]$$

where F represents the fluorescent intensity, F_{max} and F_{min} are the initial and ultimate fluorescent intensity, respectively, and G are the corresponding concentrations of guests (concentration was calculated based on the dipyridine units). Data are from n = 3 independent experiments and are presented as mean ± s.e.m. Reliable K_a of ligand **1** with peptide could not be obtained due to too small change in fluorescence spectra. [b] The calculated K_a of unit **6** and ligand **1** with peptide may not be very accurate due to too small change in fluorescence spectra.

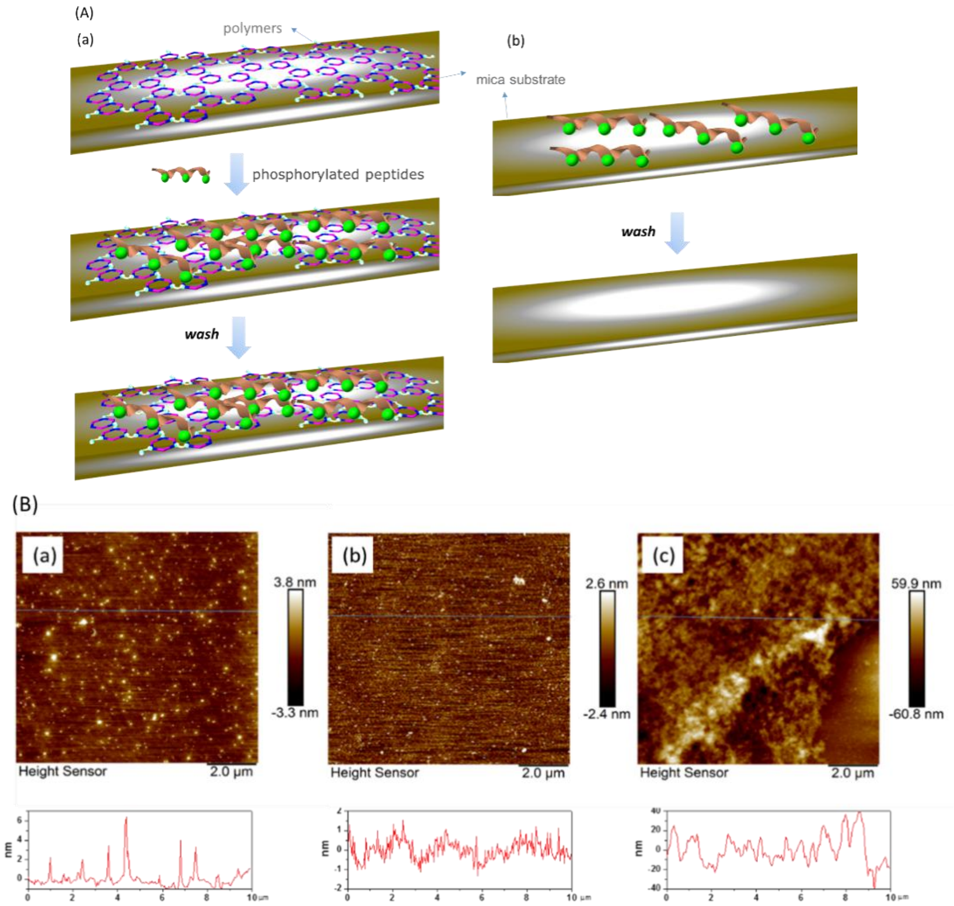

Figure S64. (A) Graphical illustration of capture phosphorylated peptides on the surface of polymeric film (a) and control experiment (b) on mica surface. (B) Typical atomic force microscopy (AFM) images of serinephosphorylated peptide 1SP (a) and after wash with pure water (b) on mica, and mica substrate coated with supramolecular polymer **5** before treatment with serinephosphorylated peptide 1SP, with subsequent wash with water (c), as well as the corresponding

section profiles along the blue lines in the AFM images. Scale bars: 2 μm. This data indicated that serinephosphorylated peptide 1SP can be captured on the substrate surface when it coated with supramolecular polymer **5**. Remarkable morphological change compared with the polymer was also observed when the polymeric film was treated with 1SP.

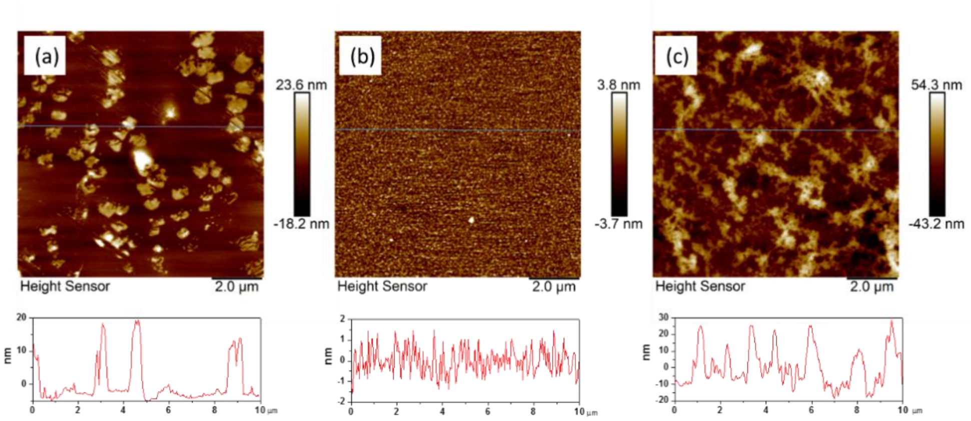

Figure S65. Typical atomic force microscopy (AFM) images of serinephosphorylated peptide 2SP (a) and after wash with pure water (b) on mica, and mica substrate coated with supramolecular polymer **5** before treatment with serinephosphorylated peptide 2SP, with subsequent wash with water (c), as well as the corresponding section profiles along the blue lines in the AFM images. Scale bars: 2 μm. This data indicated that serinephosphorylated peptide 2SP can be captured on the substrate surface when it coated with supramolecular polymer **5**. Remarkable morphological change compared with the polymer was also observed when the polymeric film was treated with 2SP.

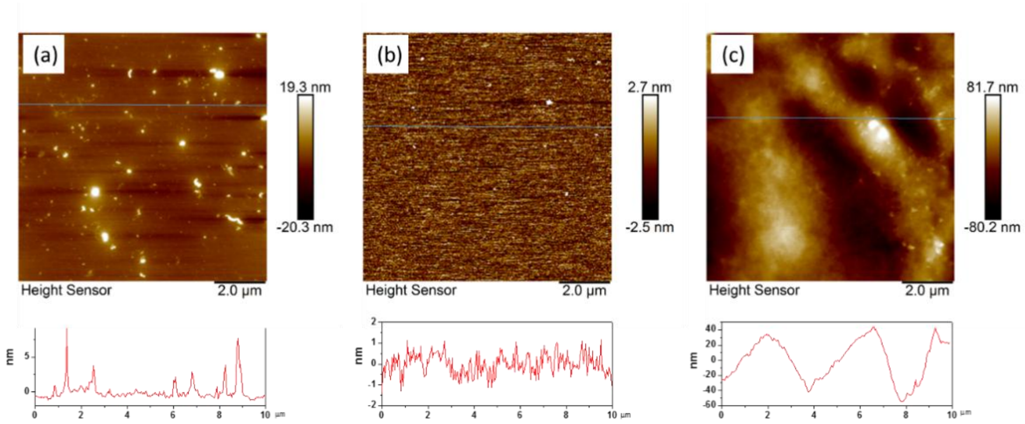

Figure S66. Typical atomic force microscopy (AFM) images of serinephosphorylated peptide 3SP (a) and after wash with pure water (b) on mica, and mica substrate coated with supramolecular polymer **5** before treatment with serinephosphorylated peptide 3SP, with subsequent wash with water (c), as well as the corresponding section profiles along the blue lines in the AFM images. Scale bars: 2 μm. This data indicated that serinephosphorylated peptide 3SP can be captured on the substrate surface when it coated with supramolecular polymer **5**. Remarkable morphological change compared with the polymer was also observed when the polymeric film was treated with 3SP.

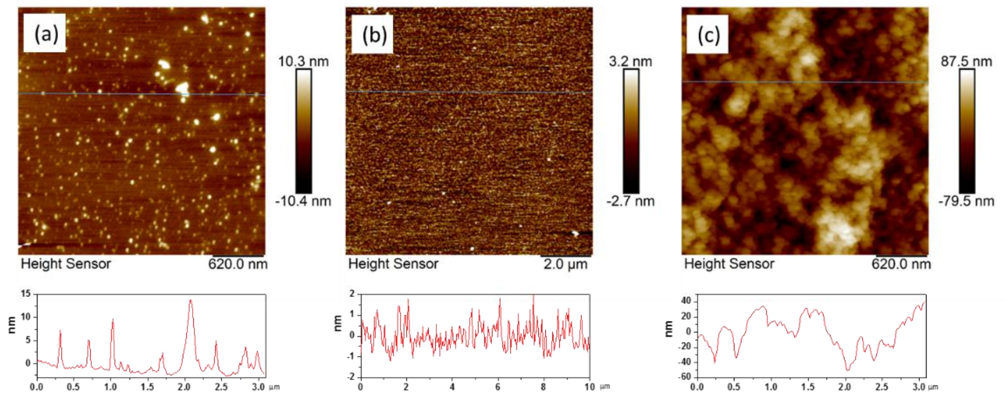

Figure S67. Typical atomic force microscopy (AFM) images of serinephosphorylated peptide 4SP (a) and after wash with pure water (b) on mica, and mica substrate coated with supramolecular polymer **5** before treatment with serinephosphorylated peptide 4SP, with subsequent wash with water (c), as well as the corresponding section profiles along the blue lines in the AFM images. Scale bars: 2 μm. This data indicated that serinephosphorylated peptide 4SP can be captured on the substrate surface when it coated with supramolecular polymer **5**. Remarkable morphological change compared with the polymer was also observed when the polymeric film was treated with 4SP.

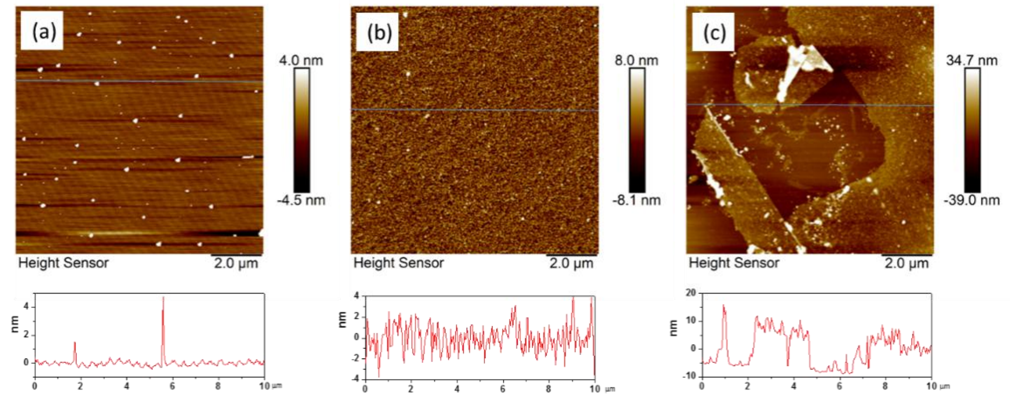

Figure S68. Typical atomic force microscopy (AFM) images of nonphosphorylated peptide NP1 (a) and after wash with pure water (b) on mica, and mica substrate coated with supramolecular polymer **5** before treatment with serinephosphorylated peptide NP1, with subsequent wash with water (c), as well as the corresponding section profiles along the blue lines in the AFM images. Scale bars: 2 μm. This data indicated that nonphosphorylated peptide NP1 can barely be captured on the substrate surface when it coated with supramolecular polymer **5**. No obvious morphological change compared with the polymer was also observed when the polymeric film was treated with NP1.

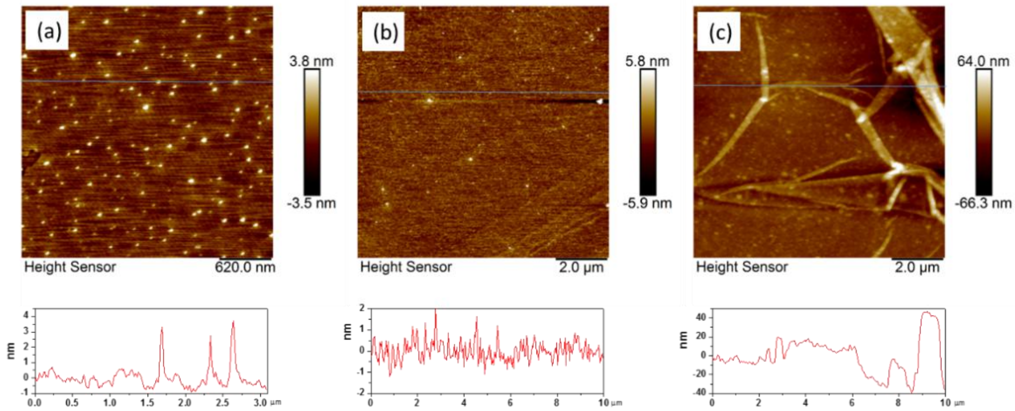

Figure S69. Typical atomic force microscopy (AFM) images of non-phosphorylated peptide NP2 (a) and after wash with pure water (b) on mica, and mica substrate coated with supramolecular polymer **5** before treatment with serinephosphorylated peptide NP2, with subsequent wash with water (c), as well as the corresponding section profiles along the blue lines in the AFM images. Scale bars: 2 μm. This data indicated that non-phosphorylated peptide NP2 can barely be captured on the substrate surface when it coated with supramolecular polymer **5**. No obvious morphological change compared with the polymer was also observed when the polymeric film was treated with NP2.

*2.4.3 Theoretical calculation of the intermolecular binding energies between metallacycle **3** and different model PPs.*

A semi-empirical method PM7 based on PM7-optimized geometries using the program MOPAC2016 was adopted to calculate the possible binding modes and binding energies between polymer **5** and different model peptides. Herein, metallacycle **3** was considered as the monomer unit of polymer 5 to simplify the calculation. Firstly, the binding energies of five typical possible binding modes between metallacycle **3** and 1SP were calculated (Figure 70). The results suggested that the skew-crossing mode (-3.4 eV, Table S3), and skew-clinging mode (-3.1 eV, Table R1) might be the dominant interaction manners between metallacycle 3 and 1SP. Two other model peptides Asp-P and Glu-P, in which the phosphorylated serine group of 1SP was replaced by other two common negatively charged amino acid groups (Asp or Glu, respectively), were calculated for comparison. As shown in Table S4, the calculated binding energy of 1SP was larger than those of Asp-P and Glu-P, although the energy difference was not very big in some cases. This result was consistent with the results obtained in the UV titration experiment in which the apparent association constant of **5** with phosphate was much higher than that with acetate. The theoretical calculation results combining with the UV titration results could provide an explanation for selectivity towards phosphopeptides.

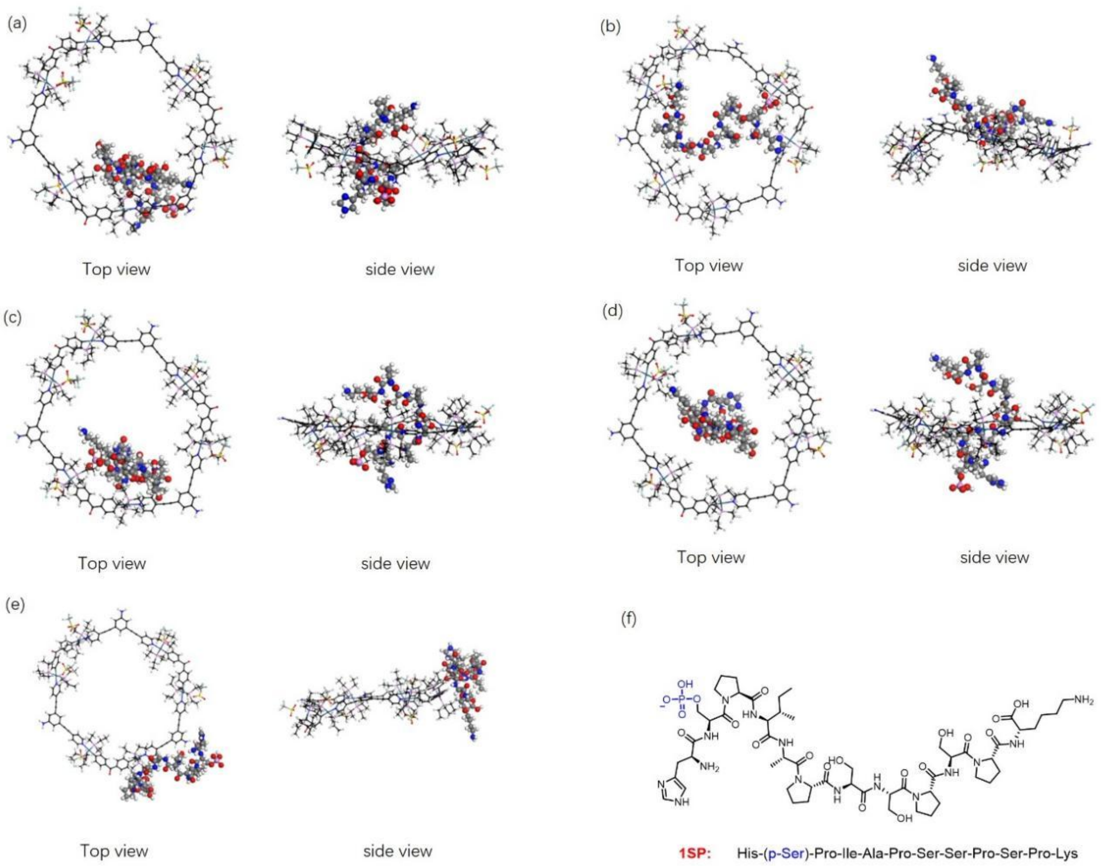

Figure 70. Simulated molecular models of possible binding modes between metallacycle **3** and peptide 1SP, (a) Skew-Crossing mode; (b) Skew-Clinging mode; (c) Side-Crossing mode; (d) Center-Crossing mode; (e) Side-Clinging mode optimized by PM7 semiempirical method, and (f) amino sequence of model peptide 1SP.

Table S3 Energy data of five possible binding modes between metallacycle 3 and peptide 1SP obtained by PM7 semiempirical molecular orbital method.

	Binding mode	E (eV)
1	Skew-Crossing mode	-3.4
2	Skew-Clinging mode	-3.1
3	Side-Crossing mode	-2.5
4	Center-Crossing mode	-1.4
5	Side-Clinging mode	-2.3

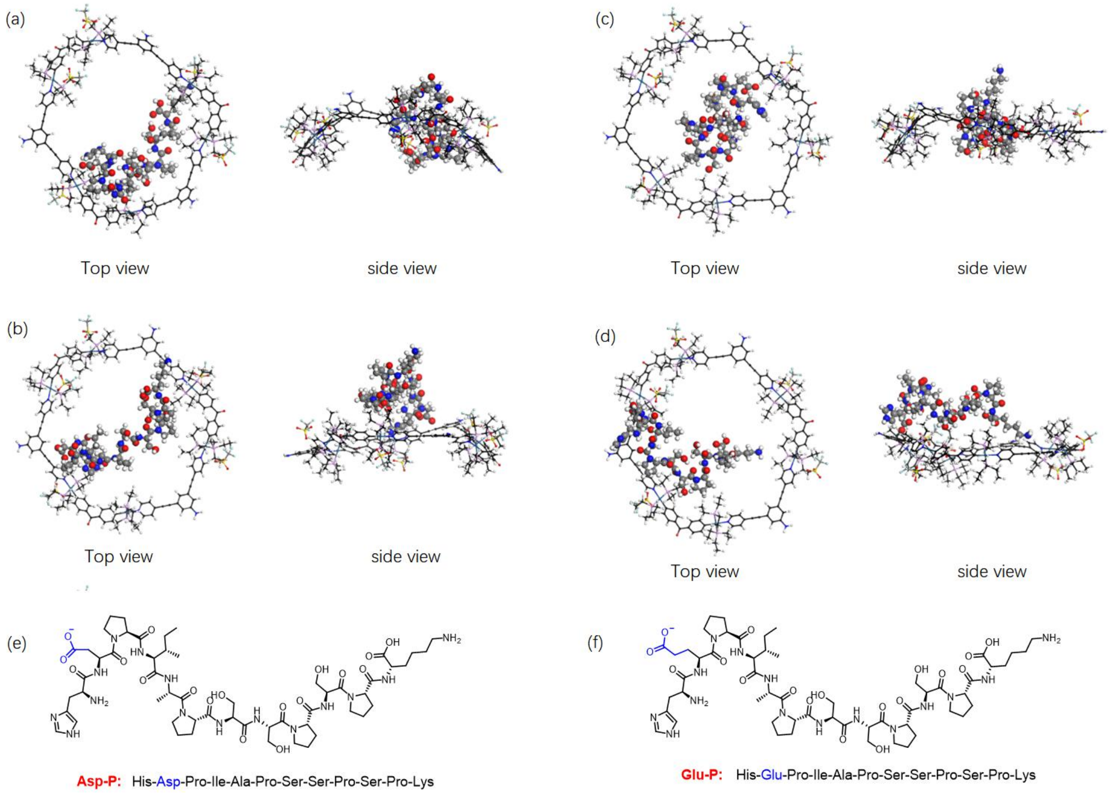

Figure 71. Simulated molecular models of possible binding modes between metallacycle **3** and peptide Asp-P and Glu-P, (a) Skew-Crossing mode of Asp-P; (b) Skew-Clinging mode of Asp-P; (d) Skew-Crossing mode of Glu-P; (e) Skew-Clinging mode of Glu-P optimized by PM7 semiempirical molecular orbital method, and amino sequence of model peptide (c) Asp-P and (f) Glu-P.

Table S4 Energy data of Skew-Crossing mode and Skew-Clinging mode between metallacycle **3** and peptide 1SP, compared with control model peptide Asp-P and Glu-P, obtained by PM7 semiempirical molecular orbital method.

	Skew-Crossing mode	Skew-Clinging mode
1SP	-3.4	-3.1
Asp-P	-3.1	-2.0
Glu-P	-2.0	-2.0

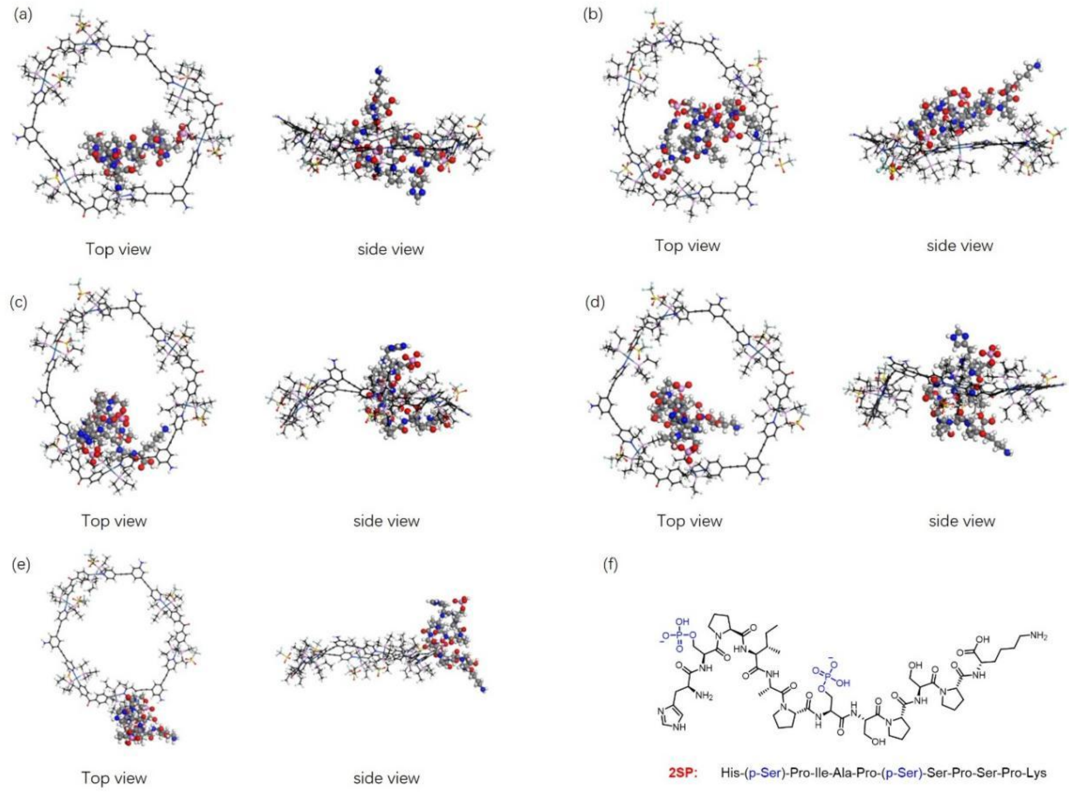

Figure S72. Simulated molecular models of possible binding modes between metallacycle **3** and peptide 2SP, (a) Skew-Crossing mode; (b) Skew-Clinging mode; (c) Side-Crossing mode; (d) Center-Crossing mode; (e) Side-Clinging mode optimized by PM7 semiempirical method, and (f) amino sequence of model peptide 2SP.

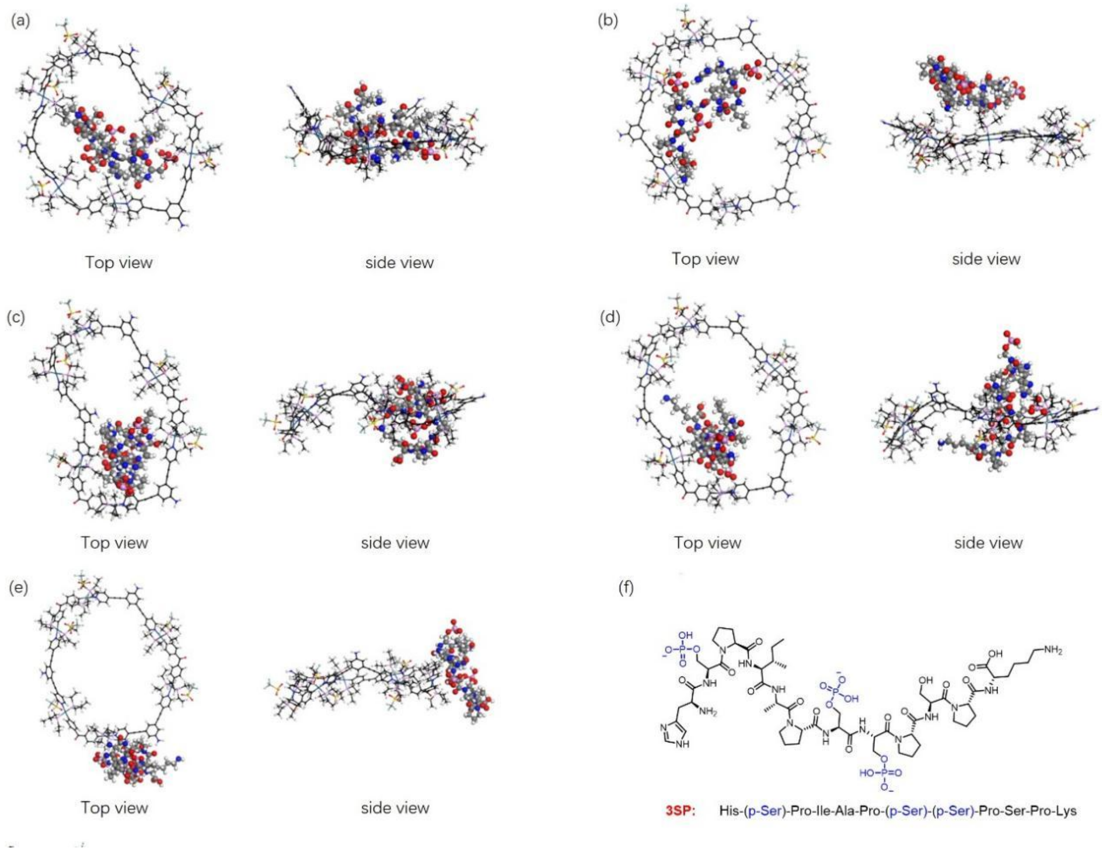

Figure S73. Simulated molecular models of possible binding modes between metallacycle **3** and peptide 3SP, (a) Skew-Crossing mode; (b) Skew-Clinging mode; (c) Side-Crossing mode; (d) Center-Crossing mode; (e) Side-Clinging mode optimized by PM7 semiempirical method, and (f) amino sequence of model peptide 3SP.

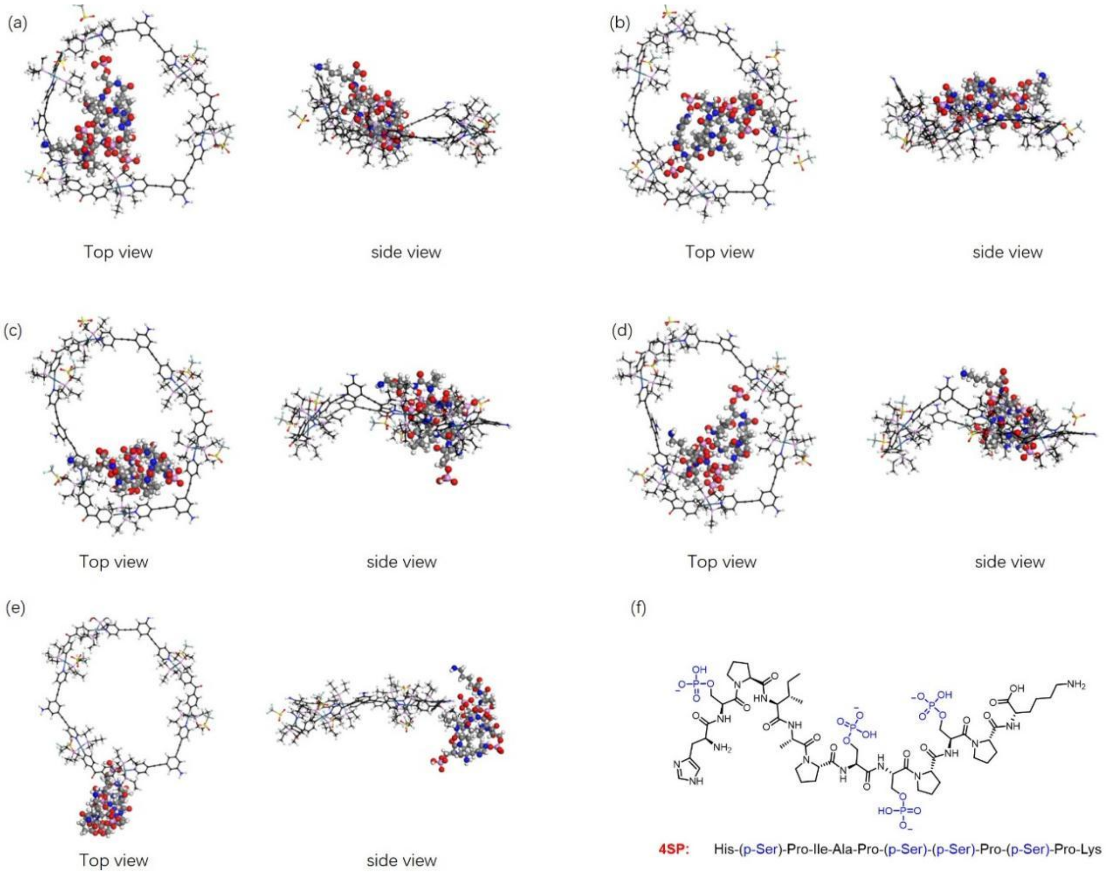

Figure S74. Simulated molecular models of possible binding modes between metallacycle **3** and peptide 4SP, (a) Skew-Crossing mode; (b) Skew-Clinging mode; (c) Side-Crossing mode; (d) Center-Crossing mode; (e) Side-Clinging mode optimized by PM7 semiempirical method, and (f) amino sequence of model peptide 4SP.

Table S5 Energy data of five possible binding modes between metallacycle **3** and peptide 1SP-4SP obtained by PM7 semiempirical method.

	skew-crossing	skew-clinging	side-crossing	centre-crossing	side-clinging
1SP	-3.4	-3.1	-2.5	-1.4	-2.3
2SP	-6.4	-3.8	-5.7	-5.0	-4.4
3SP	-8.5	-3.2	-9.1	-7.1	-6.8
4SP	-11.1	-10.6	-10.8	-12.3	-7.0

2.5. Application of polymer 5 in Selective Enrichment of Phosphopeptides.

2.5.1 Enrichment of phosphopeptides from model protein samples.

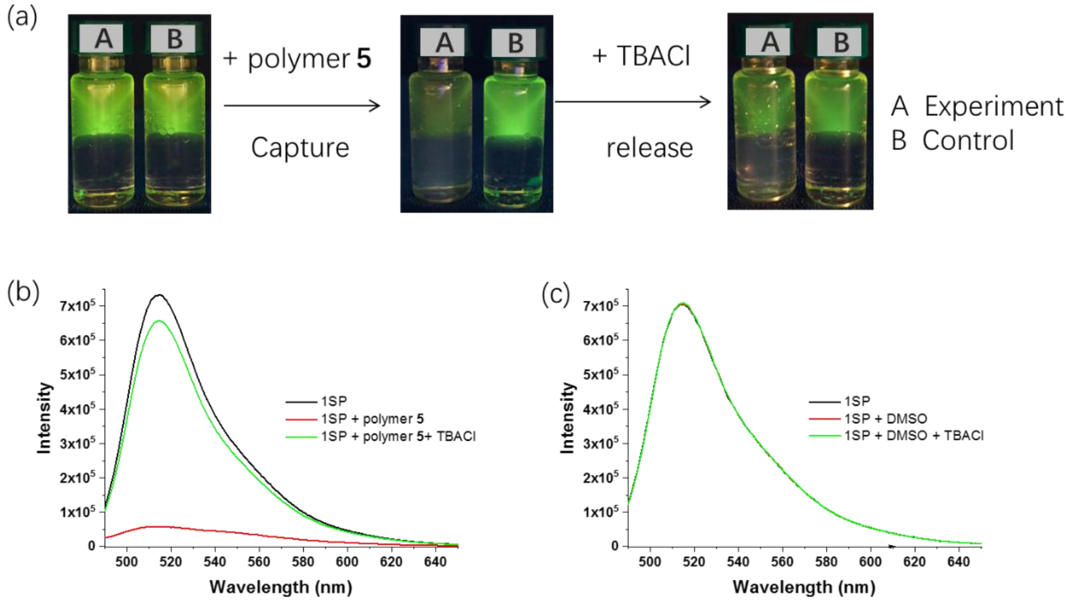

Figure S75. (a) Photographs of N-terminus fluorescein-labelled serinephosphorylated peptide 1SP solutions (1.0 × 10^{-5} mol·L^{-1}) (top layer: water; bottom layer: dichloromethane) under 365-nm irradiation, after addition of supramolecular polymer **5**, and subsequent addition of tetrabutylammonium chloride. (b) fluorescence spectra of 1SP solutions (1.0 × 10^{-6} mol·L^{-1}) after addition of supramolecular polymer **5** (1.0 × 10^{-6} mol·L^{-1}, concentrations were calculated based on the dipyridine units) or DMSO (c), and subsequent addition of tetrabutylammonium chloride.

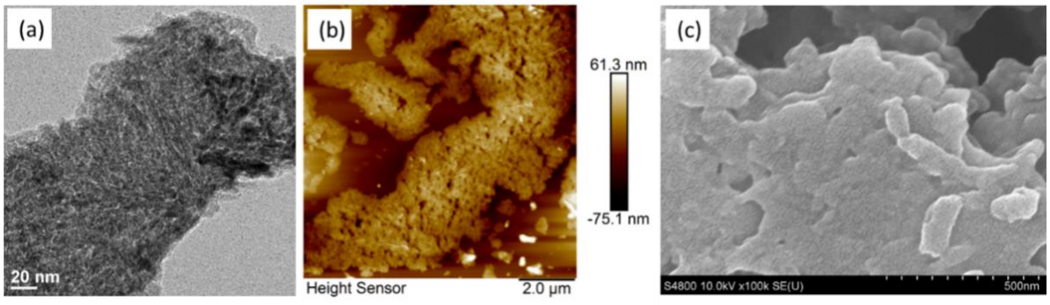

Figure S76. TEM image (a), AFM image (b) and SEM image (c) of precipitate of the supramolecular polymer **5** and serinephosphorylated peptide 1SP.

Table S6. Phosphorylated peptides identified from tryptic digest of bovine α-casein.

No.	Peptide sequence	Theoretical m/z	Found mass and charge states	Number of phosphoryl groups
1	L[pT]EEEKNR	1099.09	1099.22 (z = 1)	1
2	TVDME[pS]TEVF	1237.26	1237.45 (z = 1)	1
3	HIQKEDV[pS]ER	1337.68	1337.82 (z = 1)	1
4	EQL[pS]T[pS]EENSK	1411.53	706.24 (z = 2)	2
5	TVDME[pS]TEVFTK	1466.63	1466.73 (z = 1)<br>1385.88# (z = 1)	1
6	TVD[Mo]E[pS]TEVFTK	1482.63	741.68 (z = 2)	1
7	EQL[pS]T[pS]EENSKK	1539.63	1539.66 (z = 1)<br>1378.79# (z = 1)	2
8	VPQLEIVPN[pS]AEER	1660.81	1661.01 (z = 1)<br>830.67 (z = 2)<br>554.45 (z = 3)	1
9	YLGEYLIVPN[pS]AEER	1832.86	1832.99 (z = 1)	1
10	DIGSE[pS]TEDQAMEDIK	1847.74	1847.53 (z = 1)<br>1766.72# (z =1)	1
11	DIG[pS]E[pS]TEDQAMEDIK	1927.72	1927.89 (z = 1)<br>964.31 (z = 2)<br>1766.72# (z =1)	2
12	DIG[pS]E[pS]TEDQAMEDIK	1943.72	1943.78 (z = 1)<br>972.32 (z = 2)	2
13	YKVPQLEIVPN[pS]AEER	1951.97	1951.99 (z =1)<br>976.45 (z = 2)<br>651.29 (z = 3)	1
14	QMEAE[pS]I[pS][pS][pS]EEIVPN[pS]VEQ	2593.90	1296.91 (z = 2)<br>864.91 (z = 3) 2192.87#<br>(z = 1)	5
15	pyroQMEAE[pS]I[pS][pS][pS]EEIVPNSVEQK	2624.00	874.95 (z = 3) 2321.10#<br>(z = 1)	4
16	VNEL[pS]KDIG[pS]E[pS]TEDQAMEDIK	2678.07	2678.55 (z = 1)<br>1339.48 (z = 2)	3
17	QMEAE[pS]I[pS][pS][pS]EEIVPN[pS]VEAQ	2703.50	2703.85 (z =1)	5
18	QMEAE[pS]I[pS][pS][pS]EEIVPNPN[pS]VEQK	2720.91	2720.97 (z = 1)<br>1360.33 (z = 2)	5
19	EKVNEL[pS]KDIG[pS]E[pS]TEDQAMEDIK	2935.18	2935.01 (z = 1)<br>1468.08 (z = 2)<br>979.02 (z = 3)	3
20	FPQ[pY]LQ[pY]LYQGPIVLNPWDQVKR	3027.30	3027.53 (z = 1)	2
21	NANEEEY[pS]IG[pS][pS][pS]EE[pS]AEVATEEVK	3089.63	3090.02 (z =1)	5
22	KNTMEHV[pS][pS][pS]EE[pS]IISQETYKQEK	3132.20	3131.98 (z = 1)	4

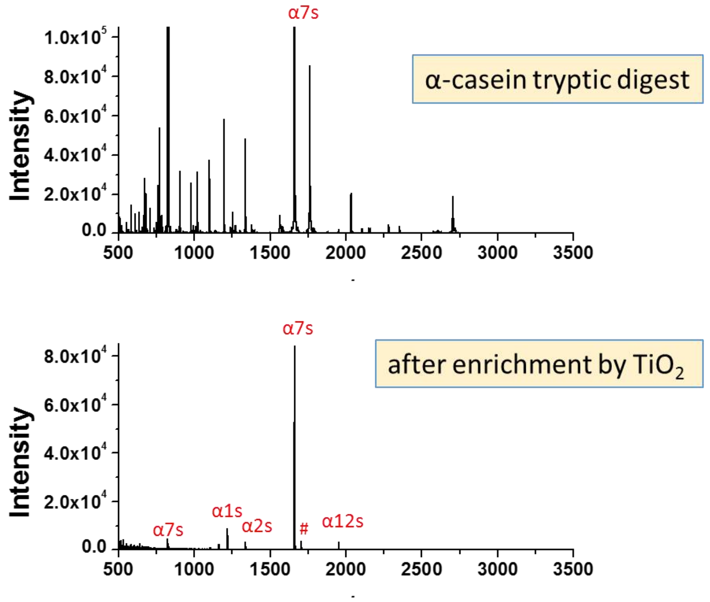

Figure S77. MALDI-TOF mass spectra of the α-casein (1 pmol) tryptic digest. Direct analysis (top), after enrichment by TiO_2 adsorbent (bottom). (s: mono-phosphopeptide; #: dephosphorylated peptide)

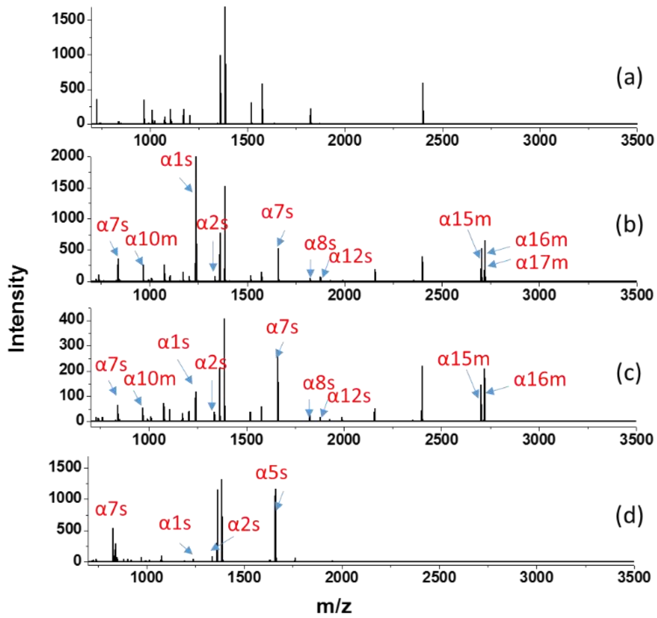

Figure S78. MALDI-TOF mass spectra of the mixture of α-casein tryptic digest (1 pmol) and BSA tryptic digest at different molar ratio enriched by polymer **5**. Direct analysis of BSA tryptic digest (a), with molar ratio of 1:20 (b), 1:100 (c) and 1:500 (d) (s, monophosphopeptide; m, multiphosphopeptide).

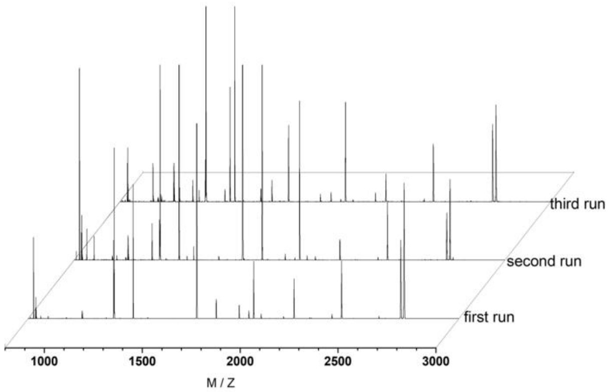

Figure S79. Three repeated results of MALDI-TOF mass spectra of α-casein tryptic digest (1 pmol) enriched by polymer **5**.

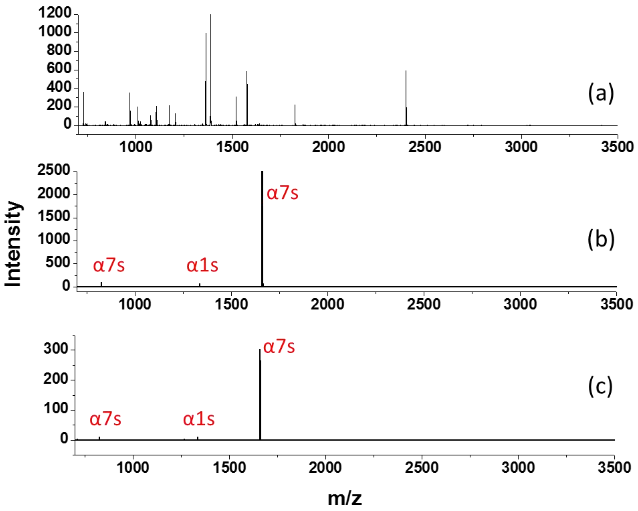

Figure S80. MALDI-TOF mass spectra of the mixture of α-casein tryptic digest (1 pmol) and BSA tryptic digest at different molar ratio enriched by TiO_2. Direct analysis of BSA tryptic digest (a), with molar ratio of 1:20 (b), and 1:100 (c) (s, monophosphopeptide).

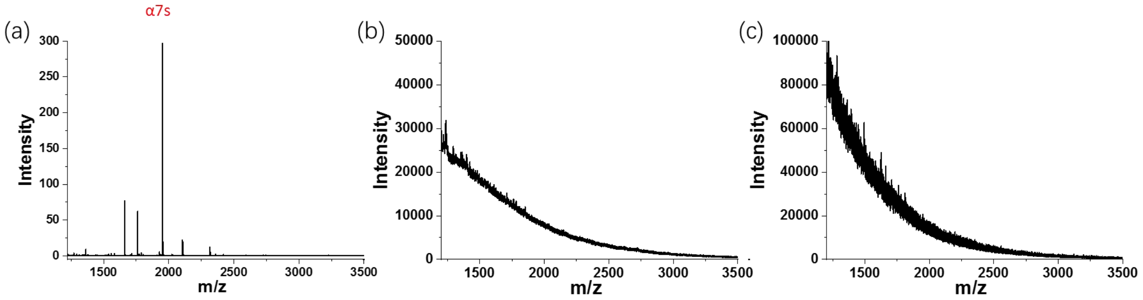

Figure 81. MALDI-TOF mass spectra of α-casein tryptic digest (1 pmol) after enrichment by (a) metallacycle **3** (s, monophosphopeptide), (b) unit **6**, (c) ligand **1**.

*2.5.2 Evaluation of the detection sensitivity, enrichment capacity, and enrichment recovery of polymer **5** in phosphopeptide enrichment.*

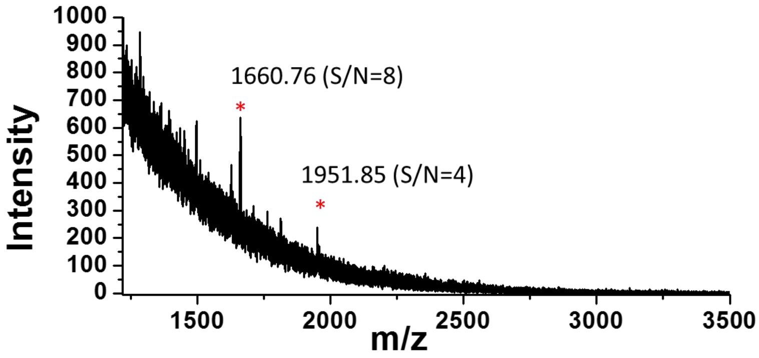

Figure S82. MALDI-TOF mass spectra of α-casein digest (2 fmol) after enrichment by polymer **5**.

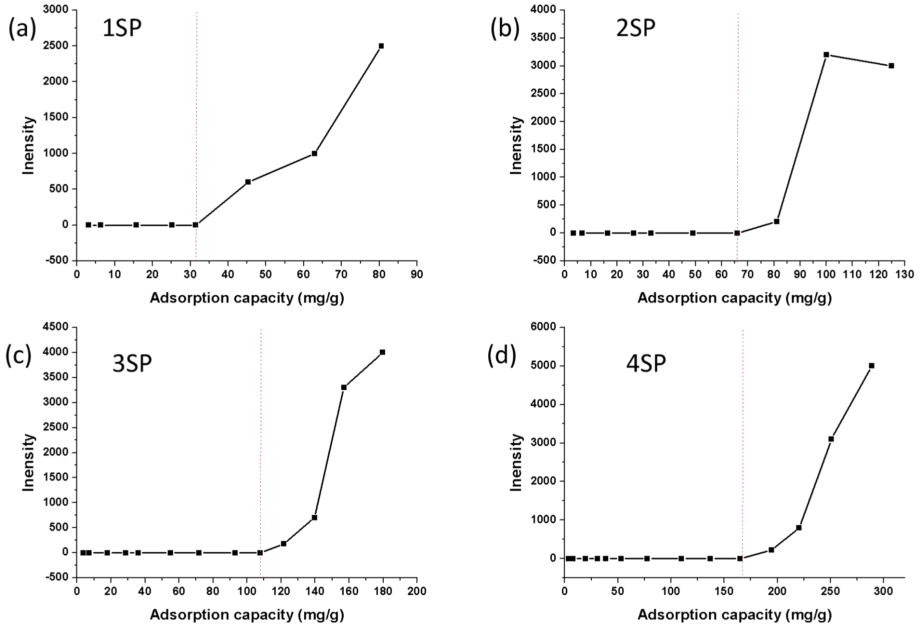

Figure S83. Determination of adsorption capacities of model serine mono-PP 1SP (a), di-PP 2SP (b), tri-PP 3SP (c) and tetra-PPs 4SP (d) with supramolecular polymer **5**. Detailed measurement method is described in Methods section. The adsorption capacity was measured as the highest PP to adsorbing material ratio at which no PP signals were observed in the MS spectra, as indicated by red dashed lines.

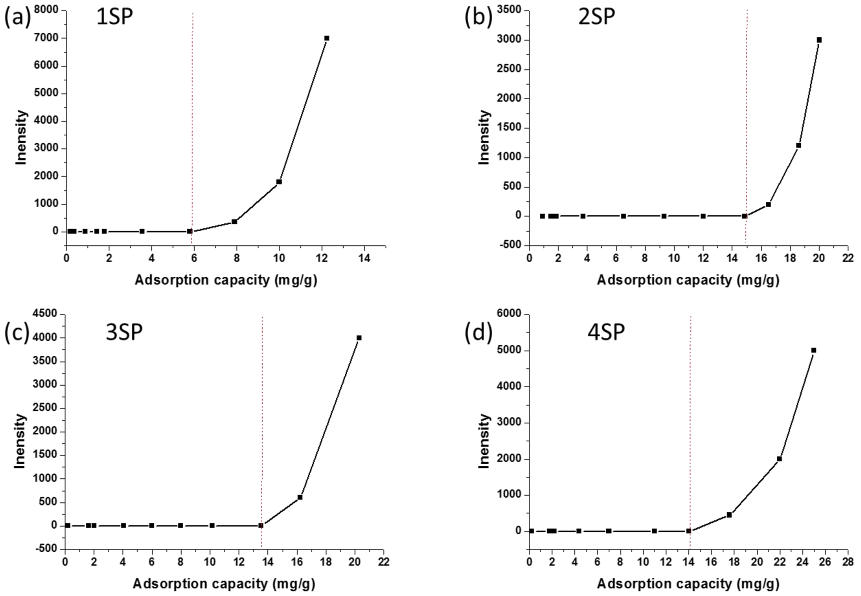

Figure S84. Determination of adsorption capacities of model serine mono-PP 1SP (a), di-PP 2SP (b), tri-PP 3SP (c) and tetra-PPs 4SP (d) on commercially available TiO_2. Detailed measurement method is described in Methods section. The adsorption capacity was measured as the highest PP to adsorbing material ratio at which no PP signals were observed in the MS spectra, as indicated by red dashed lines. The optimized PP loading conditions: 80% CH_3CN containing 1 M glycolic acid and 5% TFA.

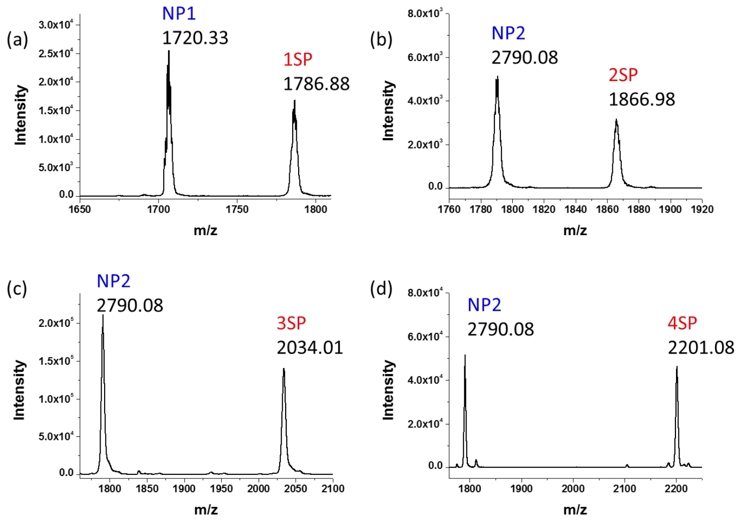

Figure S85. Determination of enrichment recovery of PPs of polymer **5** by using 1SP-4SP, NP1 and NP2 as model peptide sample. A certain amount of standard phosphopeptide (1SP-4SP, 0.5 pmol) was enriched with capture materials according to the procedure mentioned above. The eluted section was mixed with the same amount of nonphosphopeptide NP1 (0.5 pmol, a) or NP2 (0.5 pmol, c-d), and the mixed peptides were analyzed by MALDI-TOF MS. The recovery of standard phosphopeptide (1SP-4SP) was calculated by the MS intensity ratio of the peptide (1SP-4SP) divided by the nonphosphopeptide NP1 or NP2.

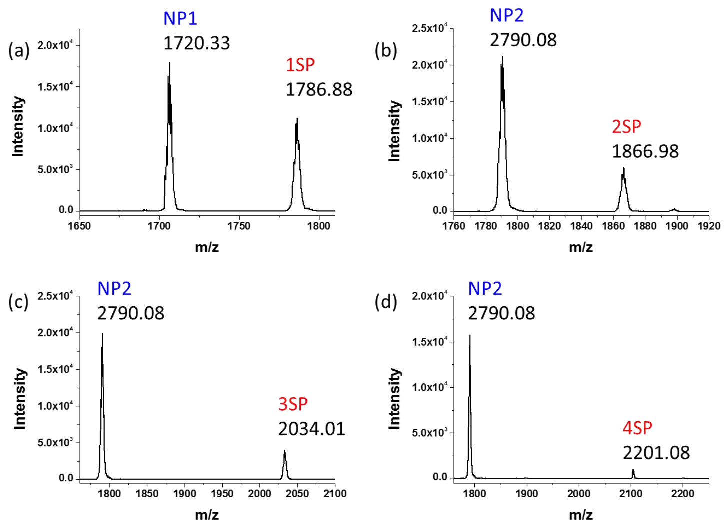

Figure S86. Determination of enrichment recovery of PPs of TiO_2 by using 1SP-4SP, NP1 and NP2 as model peptide sample. A certain amount of standard phosphopeptide (1SP-4SP, 0.5 pmol) was enriched with capture materials according to the procedure mentioned above. The eluted section was mixed with the same amount of nonphosphopeptide NP1 (0.5 pmol, a) or NP2 (0.5 pmol, c-d), and the mixed peptides were analyzed by MALDI-TOF MS. The recovery of standard phosphopeptide (1SP-4SP) was calculated by the MS intensity ratio of the peptide (1SP-4SP) divided by the nonphosphopeptide NP1 or NP2.

2.6 Application in highly specific enrichment of phosphopeptides from non-fat milk.

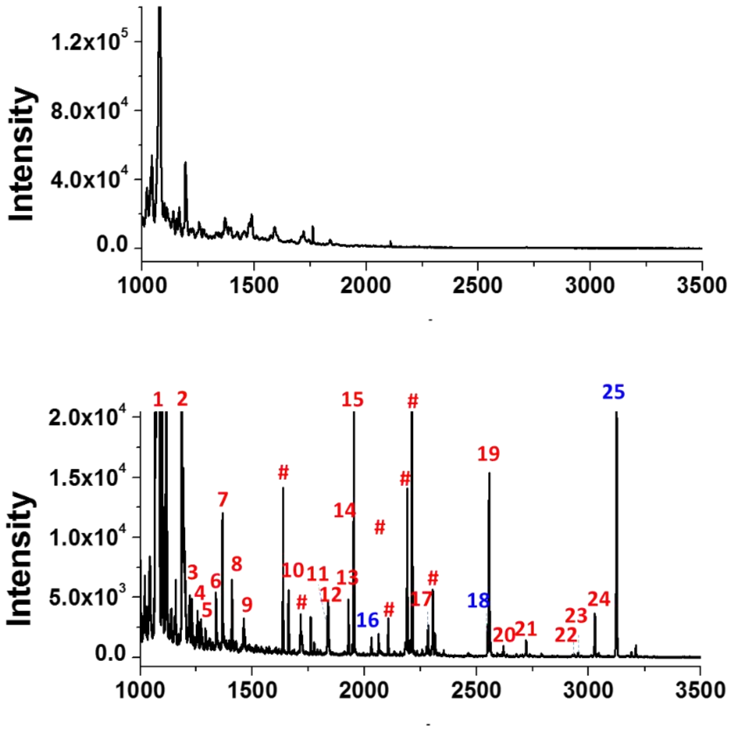

Figure S87. MALDI-TOF mass spectra of tryptic digest of the nonfat milk (top) and after enrichment by polymer **5** (bottom).

Table S7 Recovered phosphopeptides in the MALDI-MS spectrum (Fig. 5c) of tryptic low-fat milk digest after enrichment and fractionation with polymer **5**.

No.	$[M + H]^+$	No. of phosphorylation	Protein	Peptide sequence
1	1099.22	1	α-S1-casein	L[pT]EEEKNR
2	1197.21	1	α-S2-casein	KNMAINP[pS]KENL
3	1237.43	1	α-S1-casein	TVDME[pS]TEVF
4	1254.12	1	α-S2-casein	TVD[Mo]E[pS]TEVF
5	1332.23	1	α-S2-casein	EQL[pS]TSEENSK
6	1337.64	1	α-S1-casein	HIQKEDV[pS]ER
7	1411.53	2	α-S1-casein	EQL[pS]T[pS]EENSK
8	1466.28	1	α-S2-casein	TVDME[pS]TEVFTK
9	1482.67	1	α-S2-casein	TVD[Mo]E[pS]TEVFTK
10	1661.13	1	α-S1-casein	VPQLEIVPN[pS]AEER
11	1832.87	1	α-S1-casein	YLGEYLIVPN[pS]AEER
12	1847.62	1	α-S1-casein	DIGSE[pS]TEDQAMEDIK
13	1927.94	2	α-S1-casein	DIG[pS]E[pS]TEDQAMEDIK
14	1943.43	2	α-S1-casein	DIG[pS]E[pS]TEDQAMEDIK
15	1951.75	1	α-S1-casein	YKVPQLEIVPN[pS]AEER
16	2062.55	1	β-casein	FQ[pS]EEQQQTEDELQDK
17	2273.54	1	α-S1-casein	VPQLEIVPN[pS]AEERLHS[Mo]K
18	2556.22	1	β-casein	FQ[pS]EEQQQTEDELQDKIHPE
19	2564.67	1	α-S1-casein	YKVPQLEIVPN[pS]AEERLHS[Mo]K
20	2620.39	4	α-S2-casein	NTMEHV[pS][pS][pS]EE[pS]IISQETYK
21	2720.87	5	α-S1-casein	QMEAE[pS]I[pS][pS][pS]EEIVPNPN[pS]VEQK
22	2935.23	3	α-S1-casein	EKVNEL[pS]KDIG[pS]E[pS]TEDQAMEDIK
23	2952.08	3	α-S1-casein	EKVNEL[pS]KDIG[pS]E[pS]TEDQA[Mo]EDIK
24	3027.53	2	aS2-casein	FPQ[pY]LQ[pY]LYQGPIVLNPWDQVKR
25	3124.38	4	β-casein	RELEELNVPGEIVE[pS]L[pS][pS][pS]EESITR

Table S8. Comparison of phosphopeptide enrichment performance of different two-dimensional materials.

Affinity materials	Enrichment number			Ref.
	α-casein	β-casein	Non-fat milk	
rGR–TiO2–ZrO2 graphene oxides	23	8	20	S1
Fe3O4/CeO2–TiNbNS nanosheets	N/A	4	10	S2
Ti4+–SiO2 multilayer nanosheets	N/A	3	9	S3
MoO3/GO graphene oxides	N/A	5	12	S4
CaCu–Si4O10 nanosheet	N/A	3	4	S5
2-D Ti-based MOF nanosheets	6	4	5	S6
2-D Fe3O4/Cu/Ga double hydroxide	N/A	7	14	S7
2-D supramolecular polymer	22	N/A	25	This work

S1. Huang, X.; Wang, J.; Liu, C.; Guo, T.; Wang, S., A novel rGR-TiO_2-ZrO_2 composite nanosheet for capturing phosphopeptides from biosamples. *J. Mater. Chem. B* **2015,** *3*, 2505-2515.

S2. Min, Q.; Li, S.; Chen, X.; Abdel-Halim, E. S.; Jiang, L. P.; Zhu, J. J., Magnetite/Ceria-Codecorated Titanoniobate Nanosheet: A 2D Catalytic Nanoprobe for Efficient Enrichment and Programmed Dephosphorylation of Phosphopeptides. *ACS Appl. Mater. Interfaces* **2015,** *7*, 9563-9572.

S3. Xu, D.; Gao, M.; Deng, C.; Zhang, X., Ultrasensitive enrichment of phosphopeptides with Ti(4+) immobilized SiO2 graphene-like multilayer nanosheets. *Analyst* **2016,** *141* (11), 3421-3427.

S4. Sun, H.; Zhang, Q.; Zhang, L.; Zhang, W.; Zhang, L., Facile preparation of molybdenum (VI) oxide - Modified graphene oxide nanocomposite for specific enrichment of phosphopeptides. *J. Chromatogr. A* **2017,** *1521*, 36-43.

S5. Yang, S. S.; Yu, H. X.; Wang, Z. Z.; Liu, H. L.; Zhang, H.; Yu, X.; Shang, W.; Chen, G. Q.; Gu, Z. Y., An Exfoliated 2D Egyptian Blue Nanosheet for Highly Selective Enrichment of Multi-phosphorylated Peptides in Mass Spectrometric Analysis. *Chem.–Eur. J.* **2018,** *24*, 2109-2116.

S6. Yang, S. S.; Chang, Y. J.; Zhang, H.; Yu, X.; Shang, W.; Chen, G. Q.; Chen, D. D. Y.; Gu, Z. Y., Enrichment of Phosphorylated Peptides with Metal-Organic Framework Nanosheets for Serum Profiling of Diabetes and Phosphoproteomics Analysis. *Anal. Chem.* **2018,** *90,* 13796-13805.

S7. Jiang, D.; Li, X.; Jia, Q., Design of Two-Dimensional Layered Double Hydroxide Nanosheets Embedded with Fe3O4 for Highly Selective Enrichment and Isotope Labeling of Phosphopeptides. *ACS Sustain. Chem. Eng.* **2018**, *7*, 421-429.
